# Invertebrate Iridescent Viruses (*Iridoviridae*) from the Fall Armyworm, *Spodoptera frugiperda*

**DOI:** 10.3390/v18010031

**Published:** 2025-12-24

**Authors:** Birmania Rodríguez-Heredia, Jesús Alejandro Zamora-Briseño, Leonardo Velasco, Trevor Williams

**Affiliations:** 1 Instituto de Ecología AC, Xalapa 91073, Veracruz, Mexico; birmrhed@gmail.com; 2 Instituto Andaluz de Investigación y Formación Agraria (IFAPA), Churriana, 29140 Málaga, Spain; leonardo.velasco@juntadeandalucia.es

**Keywords:** *Iridovirus*, *Chloriridovirus*, infection prevalence, virus species, Mexico, Argentina, Lepidoptera, genome sequence, phylogenetic analysis

## Abstract

Invertebrate iridescent viruses (IIVs, family *Iridoviridae*) are icosahedral double-stranded DNA viruses that infect a wide range of invertebrates, particularly in humid and aquatic environments. During field trials in Chiapas, southern Mexico, larvae of the fall armyworm, *Spodoptera frugiperda* (Lepidoptera: Noctuidae), displayed an unexpected lavender iridescence, leading to the discovery of novel IIV isolates from this major agricultural pest. Restriction endonuclease analysis revealed evident diversity among isolates from individual infected larvae. Although one field experiment yielded inconclusive results, a second experiment revealed a positive association between IIV disease and SfMNPV infection, compared to a negative association with the prevalence of parasitoids, and no association with entomopathogenic nematodes (probably *Hexamermis* sp.). These findings require further investigation to determine the distinct ecological routes through which the virus may transmit across host species. IIV infection of *S. frugiperda* was also identified in Veracruz State, Mexico, and northern Argentina, revealing a previously unrecognized geographic and host range for these viruses. The genomic and evolutionary features of the three isolates from *S. frugiperda* were compared with those of two other lepidopteran isolates from *Helicoverpa zea* (IIV30C obtained from CSIRO) and *Anticarsia gemmatalis* (AgIIV). Genome sizes ranged between 196.1 and 205.4 kbp (~28% GC content), with several large inversions, and were rich in tandem repeats. The average amino acid identity of the complete genomes and phylogenetic analyses of 26 core gene sequences placed all five isolates within the genus *Chloriridovirus*, closely related to IIV22 and IV22a isolated from blackflies (Diptera) in Wales and a previously sequenced isolate of IIV30 from the USA. We conclude that these lepidopterans are all infected by closely related strains of the virus species *Chloriridovirus simulium1* across their native geographical range. These findings highlight the unexpected ecological breadth and evolutionary adaptability of IIVs.

## 1. Introduction

Invertebrate iridescent viruses (IIVs) are icosahedral dsDNA viruses (~130–185 nm diameter) that infect invertebrates, particularly in damp and aquatic habitats. IIVs from insects are classified into one of two genera (*Iridovirus*, *Chloriridovirus*) within the family *Iridoviridae* [1,2]. The genome of these viruses is a large (160–220 kbp) linear DNA molecule that is circularly permutated and terminally redundant, so that each molecule starts and terminates at a different position along the genome sequence [3].

IIVs can cause patent lethal disease characterized by a striking violet, blue or turquoise iridescence as a result of the regular packing of virus particles in the cytoplasm of infected cells. Patent disease is usually uncommon, although in some species inapparent or covert infection may be prevalent [4,5]. IIVs are not highly infectious per os but can be efficiently transmitted by wounding or injection of virus particles. As a result, laboratory transmission has been demonstrated by cannibalism and through infection by entomopathogenic nematodes. In addition, laboratory and field studies indicate that IIVs can be transmitted through oviposition by endoparasitoid wasps [6].

The fall armyworm, *Spodoptera frugiperda* (Lepidoptera: Noctuidae), is the most important pest of maize in the world. Although native to the Americas, this moth has spread across Africa and Asia over the past decade and is currently threatening Oceania and Europe [7]. During two field trials on the efficacy of Spodoptera frugiperda multiple nucleopolyhedrovirus (SfMNPV, *Baculoviridae*) in southern Mexico in 1999, it was noticed that some *S. frugiperda* larvae had an intense lavender–blue iridescence consistent with patent IIV disease. Although the SfMNPV trials gave poor results that were not published, the iridescent larvae were frozen and later used in studies to demonstrate virus transmission by cannibalism [8] and via oviposition by endoparasitoid wasps [9]. Six years later, patently infected *S. frugiperda* larvae were collected from a maize field in Veracruz state, near the Gulf coast of Mexico. IIV infection was previously reported in *S. frugiperda* larvae in northern Argentina [10], although neither the Mexican nor the Argentinian isolates have been subjected to characterization studies.

Because of the often low prevalence of lethal IIV disease in invertebrates of agricultural or medical importance, these viruses have attracted little interest as biological control agents and so studies on their diversity, biology and transmission cycles have been largely overlooked [11,12]. Furthermore, given the rapid global expansion of *S. frugiperda* over the past decade and the growing interest in the pathogens of this pest [13], we decided to reexamine the field collection data from the original collections and compare the Mexican and Argentinian IIV isolates with isolates from several other species of Lepidoptera to better understand the host–virus relationships in this charismatic group of pathogens.

## 2. Materials and Methods

### 2.1. Field Collection of IIV-Infected Larvae

IIV-infected larvae of *S. frugiperda* were collected at two different sites in Mexico in July 1999, during two field trials performed in fields of a local creole variety of maize planted close to the village of Buenos Aires, Chiapas state (14°53′27″ N, 92°28′45″ W, elevation 18 m), in southern Mexico. The trials were designed to test the efficacy of SfMNPV as a biological insecticide and treatments were applied at between 6 × 10^10^ and 6 × 10^12^ occlusion bodies/ha using a manual backpack sprayer when the plants were in the whorl stage. Control plots were not treated with occlusion bodies. The crop was divided into experimental plots of 4 × 4 m with a 3 m distance between plots. Sampling was performed at 2, 5 and 10 days post-application. In each case 10 plants from each plot were selected at random and examined to locate *S. frugiperda* larvae. The larvae were placed individually in 30 mL plastic pots with a piece of semi-synthetic diet and taken to the laboratory. Larvae were monitored daily until pupation. Deaths due to SfMNPV disease, IIV infection, parasitism by hymenopteran and dipteran parasitoids, and infection by entomopathogenic nematodes were recorded. This virus was named SfIIV-Chi given its origin in the state of Chiapas.

Six years later, two patently infected *S. frugiperda* larvae were collected by Maurilio López-Ortega (Inbioteca, Universidad Veracruzana, Mexico) in August 2005 in a maize field close to the village of Llano Grande, Veracruz state (21°30′40″ N, 98°21′31″ W, elevation 98 m), approximately 75 km inland from the coast of the Gulf of Mexico. The larvae were noticed during a field collection aimed at obtaining parasitized *S. frugiperda* larvae for a student project. This isolate was named SfIIV-Ver after the state of Veracruz.

Each IIV-infected larva was considered as a field isolate and was frozen individually at −80 °C in a microcentrifuge tube until required for this study.

### 2.2. Analysis of Field Experiments

The relationships between patent IIV infection and other natural enemies were analyzed using plot-level count data. For each experiment, observations were defined at the plot × sampling date level as the number of IIV-infected larvae out of the total larvae collected (infected/total). SfMNPV prevalence, parasitoid parasitism, and nematode infection prevalence in the same plot and sampling date were calculated from the corresponding counts and used as focal predictors in separate binomial GLMs (logit link), including treatment (virus formulations vs. controls) and sampling day (2, 5, and 10 days post-application) as fixed effects. As plots were sampled repeatedly over time, we used plot-clustered robust (sandwich) standard errors to account for within-plot correlation, which is equivalent to a population-averaged GEE approach. Models were fitted separately for each experiment in Python v.3.12 using the statsmodels module v.0.14.5 [14], and results are reported as log-odds slopes (β), odds ratios (OR), and their 95% confidence intervals for each of the three a priori ecological relationships (IIV–SfMNPV, IIV–parasitoids, IIV–nematodes).

### 2.3. Amplification of IIV Isolates

To amplify each isolate, the infected insect was homogenized in 500 µL sterile water and 8 µL of the homogenate was diluted in 300 µL sterile water and injected into groups of ten *Galleria mellonella* third instars using a disposable 1 mL insulin syringe and a microinjector. Virus amplification was performed in *G. mellonella* because it is highly susceptible to most IIVs and because SfMNPV does not replicate in this host, so any contamination by SfMNPV would be eliminated by a single passage in *G. mellonella*. Inoculated *G. mellonella* larvae were reared in 250 mL plastic tubs containing diet at 22 ± 1 °C and checked at 10 and 15 days post-inoculation for signs of patent IIV infection. Patently infected larvae were placed in 15 mL centrifuge tubes and stored at −80 °C until required.

Samples of five other lepidopteran IIVs, kindly gifted from other researchers, were also grown in *G. mellonella* and included in the study for comparative purposes (Table 1): (i) IIV6 was provided by Richard Webby and James Kalmakoff (University of Otago, New Zealand) who obtained the sample from Joe Tanada. The IIV6 genome sequence was published previously [15]. (ii) A strain of IIV9 originated from the Institute of Virology & Environmental Microbiology (IVEM, Oxford, UK) virus collection [16]. The IIV9 genome sequence was published by Wong et al. [17]. (iii) IIV30 was provided by Peter Christian (CSIRO, Canberra, Australia) from material originally stored by Adrian Gibbs (National University of Australia, Canberra) and was labeled IIV30C (from CSIRO) to differentiate it from the genome sequence from the laboratory of Yves Bigot, which we labeled IIV30B [18]. (iv) Anticarsia gemmatalis IIV (AgIIV) was obtained from Gary Kinard and Clyde B. Moore (Clemson University, USA) from material that remained after the study of Sieburth & Carner [19]. (v) An uncharacterized IIV from *S. frugiperda* isolated in Argentina [10] was kindly supplied by Maria Lucrecia Vera (PROIMI, Argentina).

### 2.4. Restriction Endonuclease Analysis

Restriction endonuclease analysis was performed as a rapid means of estimating variation among SfIIV-Chi isolates from 12 randomly selected patently infected *S. frugiperda* larvae from the field collections (labeled A–L), and to compare the restriction profile characteristics of the selected lepidopteran IIV isolates in Table 1. In each case, a patently infected *G. mellonella* larva from each isolate was homogenized in 1 mL ultrapure water, centrifuged at 700× *g* and 1100× *g* for 10 min to pellet debris and then centrifuged through a cushion of 40% (*v*/*v*) glycerol at 16,100× *g* for 30 min. The resulting pellet was washed once with ultrapure water and was resuspended in 500 µL ultrapure water. A 300 µL volume of the suspension was diluted to 1 mL in TE buffer (10 mM Tris-HCl, 1 mM EDTA, pH 8.0) with 20 µL of 10% sodium dodecyl sulfate (SDS) and 20 µL of proteinase K. The mixture was incubated overnight at 38 °C and then subjected to phenol-chloroform extraction and ethanol precipitation of DNA. A 1 µg sample of each DNA was digested overnight with HindIII or EcoRI (New England Biolabs, Ipswich, MA, USA), subjected to electrophoresis overnight in 0.7% agarose, stained with ethidium bromide and photographed on a transilluminator (UV ChemiDoc XRS + System with Image Lab Software v.6.1; Bio-Rad, Hercules, CA, USA). Replicate digests (HindIII and EcoRI) from three different insect larvae were also performed to ensure that the amplification host did not influence the genomic DNA restriction profile and that the amplification of each isolate was consistent among the inoculated larvae of *S. frugiperda* and *G. mellonella*, and showed no evidence of cross-contamination among isolates.

### 2.5. Genome Sequencing of Virus Isolates

Viral isolates from lepidopterans (SfIIV-Chi, SfIIV-Ver, SfIIV-Arg, AgIIV and IIV30C) were selected for genomic analysis. DNA was extracted from purified virus suspensions using the DNeasy Blood & Tissue Kit (Qiagen, Hilden, Germany) following the manufacturer’s instructions. All isolates were sequenced with Nanopore technology at the Secoya Laboratory (Mexico City, Mexico), using the wf-amplicon workflow on a MinION device (Oxford Nanopore Technologies, Oxford, UK), targeting amplicons larger than 600 bp. Read quality was assessed with FastQC v.0.12.1, and low-quality reads (Q < 10), adapter sequences and short sequences (<1000 bp) were removed using Filtlong v.0.2.0 [23]. Additionally, SfIIV-Chi was sequenced with Illumina technology (Novogene, Sacramento, CA, USA), using the NovaSeq 6000 platform (Illumina Inc., San Diego, CA, USA) and the TruSeq DNA PCR-Free Library Prep Kit (Illumina Inc., San Diego, CA, USA). Raw reads were processed with Trimmomatic v.0.39 [24], applying the parameters Illuminaclip v.2.30.10, Slidingwindow v.4.20, and a minimum length of 50 bp. Post-filtering quality was verified with FastQC v.0.12.1.

#### 2.5.1. Genome Assembly and Annotation

Genomes were assembled using a combination of long-read and hybrid strategies to maximize contiguity and accuracy. Nanopore reads were assembled using Flye v.2.9 [25] with the nano-corr option and 1% error threshold. For the SfIIV-Chi isolate with both Nanopore and Illumina data a hybrid assembly was generated using Unicycler v.0.4.8 [26] in bold mode to enhance structural resolution. Assembly quality was assessed with Quast v.5.2.0 [27] using five standard metrics: number of contigs, longest contig, total assembly length, N50 (≥1000 bp), and GC content. In silico digestions were performed in Python using BioPython v.1.86 (SeqIO, RestrictionBatch Analysis) and a set of custom scripts. Each genome was computationally cut using HindIII and EcoRI.

Initial functional annotation of the viral genomes was performed using Prokka v.1.14.5 [28]. The annotation was subsequently refined and functionally characterized through BLASTp searches against the UniProtKB/Swiss-Prot database to ensure high-quality annotations. In addition, orthology-based functional categories were assigned using eggNOG-mapper [29], facilitating broader functional classification and pathway inference.

As these pipelines are generalist, we additionally re-annotated all genomes with the virus-focused tool VIGA [30]. For a curated set of iridovirus hallmark genes, VIGA predictions were compared against Prokka annotations by BLASTp and by visual inspection of gene boundaries and synteny relative to IIV6. No internal stop codons, frameshifts or gene splits were detected. Proteins classified as hypothetical in Prokka were retained as such when they lacked significant similarity in BLASTp searches against UniProt/Swiss-Prot (E-value > 1 × 10^−6^, identity < 30%, or query coverage < 60%) and when eggNOG-mapper did not assign a known COG category or functional description.

Transmembrane motifs were predicted using DeepTMHMM ver 1.0.24 [31] on the amino acid sequences generated by Prokka for each genome. Entries identified as alpha-helices were extracted and counted per locus tag to determine the number of transmembrane helices associated with each coding sequence.

These procedures allowed for consistent identification of coding sequences and putative gene functions across all isolates. The annotation of circularly permutated genomes was visualized using CGView v.2.0.3 [32].

#### 2.5.2. Genome Similarity and Synteny Analyses

Genomic similarity among isolates was assessed using average amino acid identity (AAI). A curated dataset of 51 representative *Iridoviridae* genomes was compiled from the NCBI and ICTV databases (Supplementary Table S1), selected based on taxonomic classification, host range and annotation completeness. All genomes, including our five sequenced isolates, were re-annotated using Prokka v.1.14.5. AAI was estimated with CompareM v.0.0.23 [33], aligning predicted coding sequences (CDS). The resulting AAI matrix was filtered with a 50% similarity threshold to remove low-confidence comparisons and used as a basis for downstream evolutionary analyses. Similarly, pairwise average nucleotide identity (ANI) values were calculated using FastANI v1.33 with default parameters (fragment length = 3000 bp). For pairs where FastANI did not report an ANI estimate, or for which ANI values were <70%, the corresponding cells were labeled ≤70% and treated as low-confidence/distant comparisons.

Genomic synteny among isolates was examined using progressiveMauve [34] to identify locally collinear blocks (LCBs) and to assess structural conservation across genomes. For this, the AgIIV genome was specified as the initial reference genome.

### 2.6. Phylogenetic Reconstruction

Phylogenetic relationships were inferred using predicted protein sequences from 57 re-annotated iridovirid genomes. A core-gene tree was built using a curated set of conserved viral genes previously defined for *Iridoviridae* [35]. We compiled a set of 26 previously defined core genes and searched for homologs in our dataset using BLASTp (e-value < 1 × 10^−5^) against a custom protein database containing all predicted proteins from the 57 genomes and validated as single-copy orthologs using OrthoFinder v.2.5.5 [36]. Sequences were filtered with MMseqs2 [37] to ensure orthology and adequate length, aligned with Mafft v.7.467 [38], and trimmed with trimAl [39] to remove poorly aligned regions. The concatenated amino acid alignment was analyzed in IQ-Tree2 [40] under a maximum likelihood framework with 1000 ultrafast bootstrap replicates. The resulting tree was drawn using Chiplot [41].

In parallel, the same workflow was applied to four conserved core genes individually, namely the major capsid protein (MCP), myristylated membrane protein (456R), RNase II and DNA polymerase (DNApol) to construct gene-specific phylogenies and assess topological congruence among the generated trees. Maximum-likelihood trees were then inferred with IQ-TREE2 v2.2.6 under the LG + G4 substitution model, using 1000 ultrafast bootstrap (UFBoot) replicates and 1000 SH-aLRT replicates to assess branch support. Spearman’s rank correlation coefficient was calculated for each tree separately using Scipy v.1.11.1 [42].

## 3. Results

### 3.1. Field Collection of IIV-Infected Larvae

A total of 3954 *S. frugiperda* larvae were collected in the first field experiment and 2750 larvae in the second experiment in Chiapas state (Table 2, File S1).

**Figure 1:**
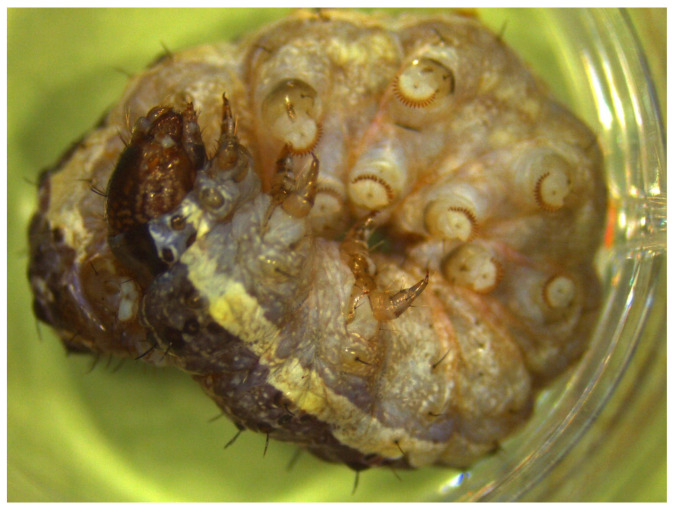
An infected *Spodoptera frugiperda* larva showing the bluish iridescence that is characteristic of patent IIV disease.

The number of larvae that exhibited patent IIV disease varied from 0 to 16 larvae collected on each sample date from SfMNPV-treated and control plots in the first experiment (overall 0.86% patent infection), compared to between 1 and 8 infected larvae across the SfMNPV-treated and control plots in the second experiment (overall 1.05% patent infection). This resulted in the collection of a total of 63 larvae with patent IIV disease (hereafter referred to as SfIIV-Chi). The presence of IIV infection was evident from the lavender-blue hue of larvae which is characteristic of patent disease in this group of viruses (Figure 1).

Although relatively high concentrations of SfMNPV OBs were applied to treatment plots, the prevalence of lethal polyhedrosis disease was moderate or low in both experiments (6.73–33.96%). The prevalence of natural SfMNPV disease was consistently low in control plots in both experiments (0–1.3%). Parasitism by hymenopteran and dipteran parasitoids was similar in both experiments and ranged from 5.19% to 31.97% across control and SfMNPV-treated plots (Table 2). Similarly, infection by nematodes was observed in treatment and control plots at a prevalence of 1.55–12.99%.

**Figure 2:**
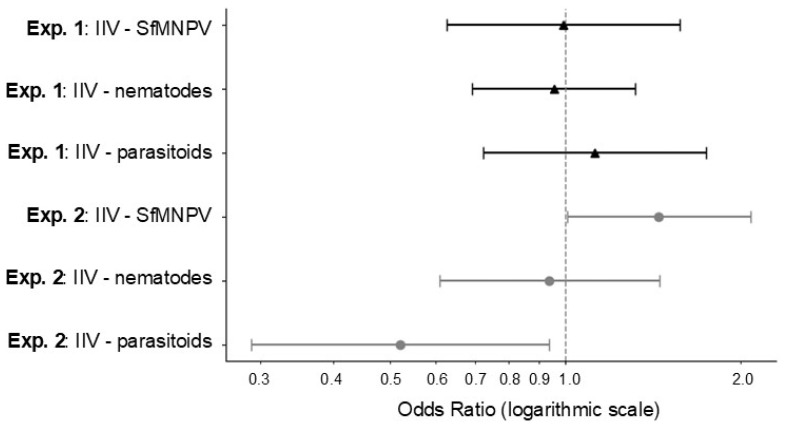
Odds ratio and 95% confidence interval for the prevalence of IIV infection in relation to SfMNPV infection, nematode infection and parasitism by parasitoids in *S. frugiperda* larvae from two field experiments (Exp. 1, black triangles and lines; Exp. 2, gray points and lines) in Chiapas State, Mexico.

To investigate the relationship between IIV disease and the other natural enemies, each experiment was analyzed separately. In the first experiment, none of the natural enemies had a significant association with the prevalence of IIV disease as in all cases, the 95% confidence intervals of the odds ratios overlapped 1.0 (Figure 2, Table S2). In contrast, in the second experiment, the prevalence of IIV infection was positively associated with SfMNPV disease (slope ± SE, β = 0.370 ± 0.185; odds ratio = 1.448, 95% CI 1.007–2.082) and negatively associated with parasitism by parasitoids (β = -0.652 ± 0.301; odds ratio = 0.521, 95% CI 0.289–0.940). There was no relationship between IIV infection and the prevalence of nematode infection (β = -0.047 ± 0.222, odds ratio = 0.939, 95% CI 0.608, 1.450) (Figure 2).

In the field collection performed in Veracruz state, Mexico, in 2005, two patently IIV-infected *S. frugiperda* larvae were collected (named SfIIV-Ver), but no information is available on the prevalence of parasitism or other natural enemies in the local *S. frugiperda* population.

### 3.2. Restriction Endonuclease Analyses

**Figure 3:**
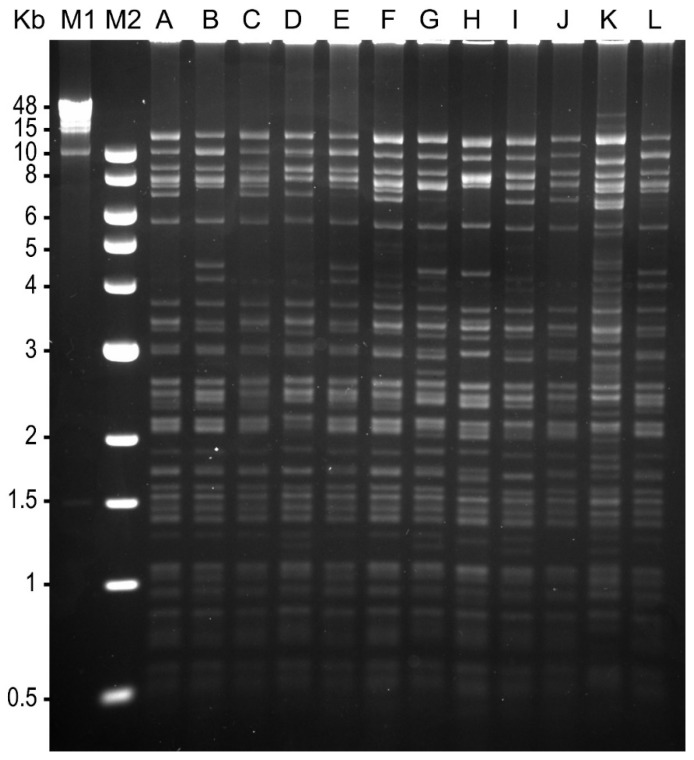
Restriction endonuclease fragment length polymorphism of SfIIV isolates from Chiapas (SfIIV-Chi), Mexico following digestion with HindIII. Twelve isolates from individual infected *Spodoptera frugiperda* larvae are labeled A–L. Molecular size markers are lambda phage-HindIII (M1) and New England Biolabs 1 Kb ladder (M2).

Analysis of a random selection of twelve (A–L) of the SfIIV-Chi isolates from individual patently infected larvae revealed a notable prevalence of polymorphic fragments, indicating that each patently infected *S. frugiperda* larva was infected by a shared but slightly different strain of this virus. Similar results were obtained with both HindIII (Figure 3) and EcoRI (Supplementary Figure S1) although the number of polymorphic fragments was lower in EcoRI compared to HindIII-treated DNA. Based on these results, a single SfIIV-Chi isolate (Figure 3, lane C) was selected for inclusion in the genomic sequencing study.

**Figure 4:**
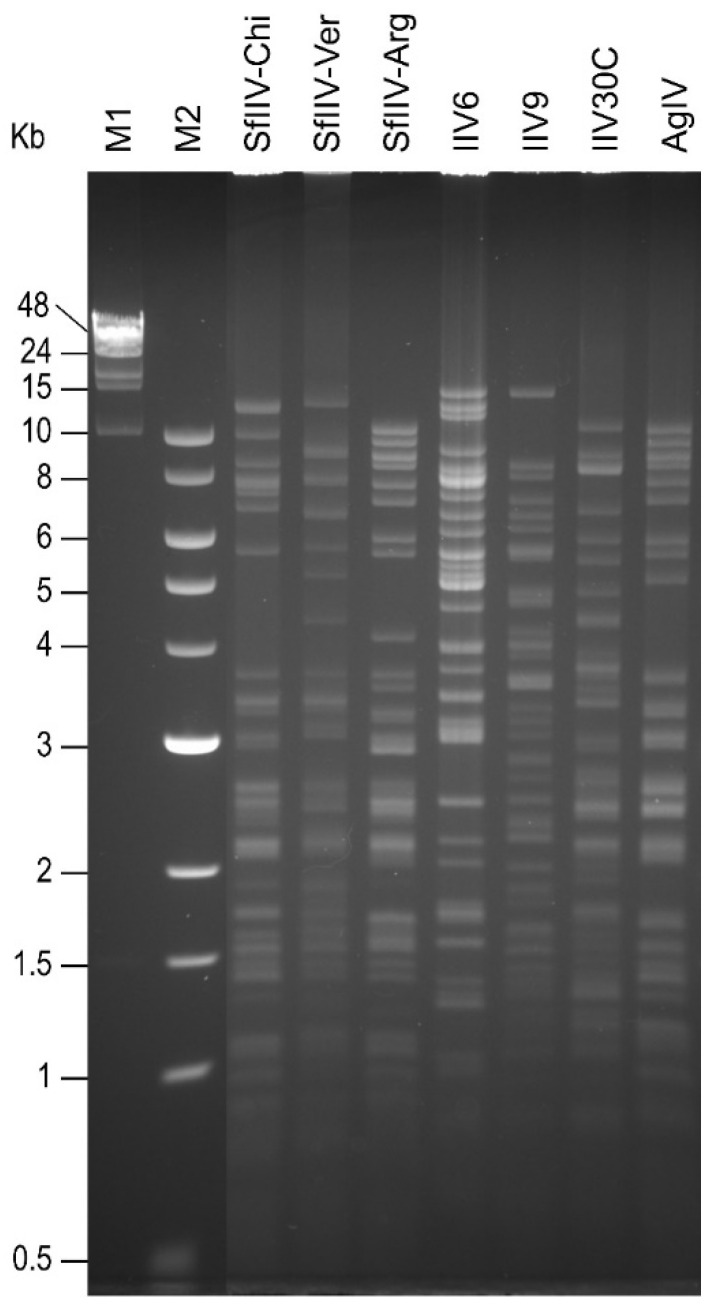
Restriction endonuclease fragment length polymorphism of seven IIV isolates from lepidopteran hosts following digestion with HindIII. Molecular size markers are lambda phage-HindIII (M1) and New England Biolabs 1 Kb ladder (M2).

Similarly, comparison of the HindIII restriction profiles of the isolates from *S. frugiperda* revealed that the isolates from Mexico (SfIIV-Chi and SfIIV-Ver) could be readily distinguished from one another and were clearly distinct from the isolate from Argentina (SfIIV-Arg), or from the other IIVs from lepidopteran larvae (IIV6, IIV9, IIV30C, or AgIIV) (Figure 4). A very similar picture emerged when the EcoRI restriction profiles were compared (Figure S2).

To validate these findings, replicate digests of genomic viral DNA extracted from isolates amplified in *S. frugiperda* and *G. mellonella* larvae demonstrated that for SfIIV-Chi, SfIIV-Ver and SfIIV-Arg, (i) the host species did not influence the restriction endonuclease profiles and (ii) the profiles from different individual insects were identical indicating that virus amplification was consistent across individual hosts for both HindIII and EcoRI (Figure S3).

In silico digestion using HindIII resulted in 97–107 fragments for the SfIIV isolates compared to 57–104 fragments for the IIVs isolated from other lepidopteran hosts (Figure S4; File S2). Similarly, EcoRI digestion resulted in 20–25 fragments for each of the SfIIV isolates compared to 23–46 fragments for the isolates from lepidopteran hosts. However, comparison with the empirical results was of limited value as are large fraction of the fragments were less than 1 kb in length and were not resolved on 0.7% agarose.

### 3.3. Genome Assembly and Annotation

**Figure 5:**
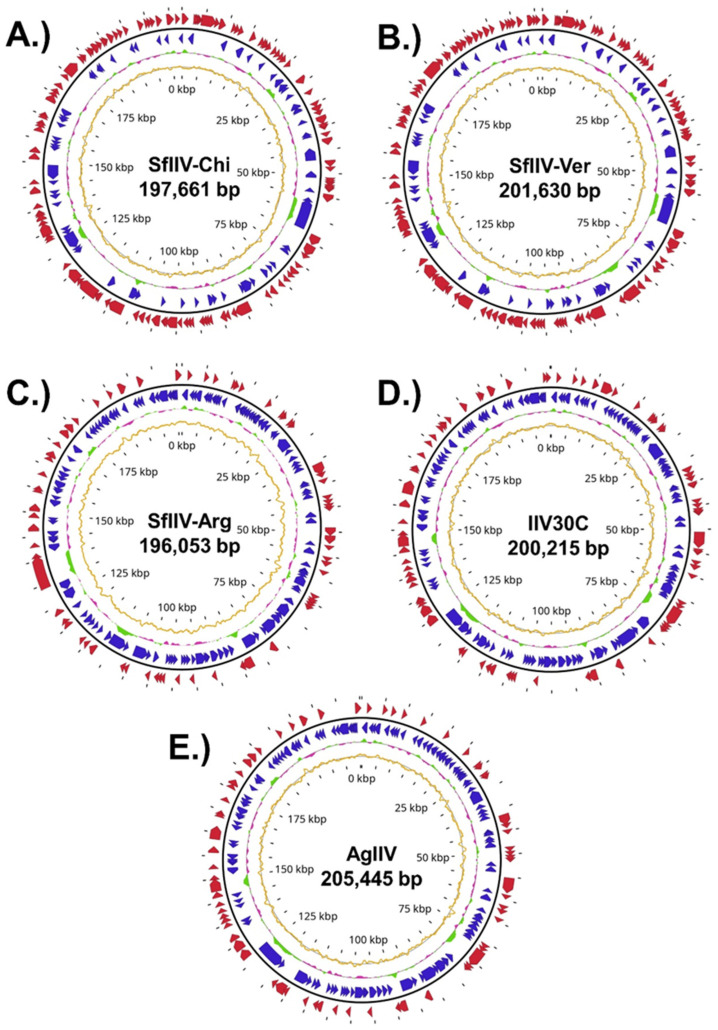
Circular genome maps of the five sequenced isolates from Lepidoptera. (**A**) SfIIV-Chi, (**B**) SfIIV-Ver, (**C**) SfIIV-Arg, (**D**) IIV30C, and (**E**) AgIIV. From the center outward: the inner track represents the GC skew (gold line). The second track displays the relative GC content (%), where green regions denote GC values above the genomic average and magenta regions indicate below-average GC content. The third track indicates genes encoded on the reverse strand (blue arrows), and the outermost track represents genes on the forward strand (red arrows). The numerical inner scale indicates genome position in kilobases (kbp) starting at the *mcp* gene. Detailed annotations are provided in Supplementary Tables S6–S10 and linearly annotated genomes are presented in Supplementary Figures S5–S9.

Assemblies for SfIIV-Chi, SfIIV-Ver, SfIIV-Arg, IIV30C, and AgIIV were consistently resolved into a single contig (Table S3). Flye produced contiguous assemblies with lengths that fell within the expected size range for these viruses with coverage values between 7× (IIV30C) and 32× (AgIIV) and mapping percentages consistently greater than 95%, with the exception of IIV30C (51.34%) (Table S3). Hybrid assembly (ONT + Illumina) of SfIIV-Chi using Unicycler produced a single contig comparable in length and structure to the ONT-only Flye assembly. No structural differences or improvements in contiguity were observed, as the long-read data already spanned all repetitive regions. For consistency, Flye assembly was retained as the final genome, and Illumina reads were used only for polishing and validation. Annotation of genomic features revealed that all five genomes shared a highly conserved architecture. Genome lengths ranged from 196.1 to 205.4 kbp, with a consistently low GC content (~28%) characteristic of iridoviruses (Table 3). In all cases, coding sequences were distributed evenly along the circularly permutated genome on both forward and reverse strands (Figure 5A–E).

The number of predicted ORFs ranged from 184 to 196 in the three isolates from *S. frugiperda* whereas the highest number of predicted ORFs (n = 205) was observed in the IIV30C isolate compared to 195 ORFs in AgIIV (Table 3). In all cases, a substantial fraction (43–53 per genome) of the ORFs was annotated as hypothetical proteins. All genomes also contained multiple tandem repeat regions (66–88 per genome), previously reported in IIV genomes.

Global GC skew analysis further supported these fine-scale distinctions. SfIIV-Arg, IIV30C, and AgIIV exhibited a modest bias toward cytosine content (–2.98% to –3.52%), whereas SfIIV-Chi and SfIIV-Ver displayed a slight guanine bias (~+3.5%). Strand asymmetry also differed, with IIV30C and AgIIV showing the lowest cumulative amplitude, whereas the three SfIIV isolates reached the highest values. Together, these observations indicate subtle, lineage-specific differences in nucleotide composition and strand bias, despite the overall conservation of genome architecture among these isolates (Figure 5).

Functional classification also revealed subtle variations in the proportion of ORFs assigned to replication, recombination, and repair (COG L-category), which was highest in IIV30C (2.9%) and lowest in SfIIV-Ver (1.6%), while AgIIV showed a slightly higher fraction of genes linked to transcription (COG K-category, 1.0%) compared to the other genomes (0.5%). Despite these differences, all genomes shared a marked prevalence (~33–34%) of S-category proteins of unknown function (Table S4).

We predicted transmembrane helices in 9.1–10.3% of the coding sequences (CDS) across the five genomes (18–20 proteins per genome) (Table S5). Among the hypothetical proteins, 11.3–14.9% contained at least one predicted transmembrane domain, indicating that a non-negligible subset of uncharacterized ORFs likely corresponds to membrane-associated or structural components.

### 3.4. Genome Similarity and Synteny Analyses

**Figure 6:**
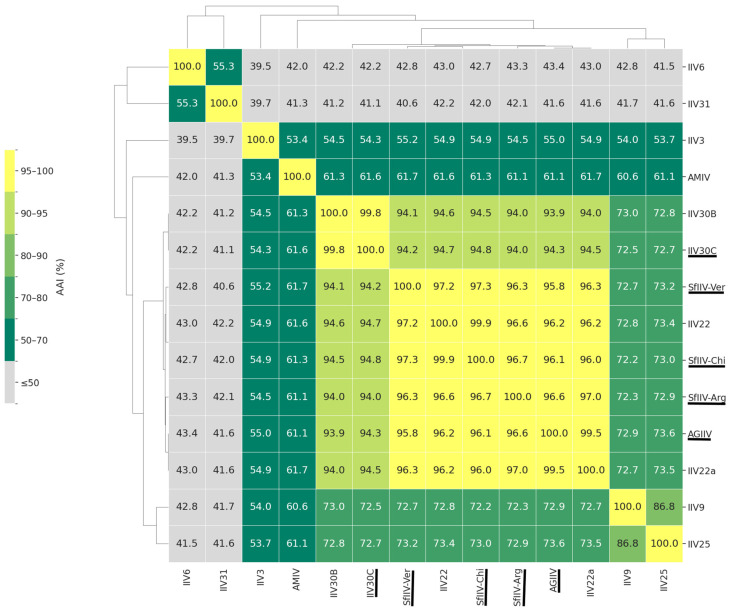
Heatmap of the pairwise average amino acid identity (AAI) percentage values for IIVs in the *Iridovirus* and *Chloriridovirus* genera calculated using CompareM on complete genomes. Viruses sequenced in the present study are underlined.

To assess protein-level relatedness across the *Iridoviridae* family, pairwise average amino acid identity (AAI) values were calculated for the complete genomes of five sequenced isolates from Lepidoptera and other known IIVs. The three isolates from *S. frugiperda* were nearly identical to one another (96.1–97.3% identity) and were strikingly similar to IIV22 (96.6–99.9% identity) originally isolated from *Simulium* sp. (Diptera: Simuliidae). IIV30C was near identical (99.8% identity) to the previously reported genome of this virus that we named IIV30B from the laboratory of Yves Bigot [18]. IIV30C presented 94.0–94.8% identity to the other lepidopteran IIVs that we sequenced, whereas other members of the *Chloriridovirus* genus had identities of ~73% (IIV9, IIV25) or ~55–61% for the viruses of mosquitoes (IIV3, AMIV). The members of the *Iridovirus* genus (IIV6, IIV31) were well delineated with identity values of ~39–43% (Figure 6). These findings are consistent with the AAI values specified by the ICTV for demarcation of genera and species in which members of the same genus share >50% sequence identity and members of the same species share >95% identity across the 26 core genes [43].

The pairwise ANI matrix provided a similar pattern of relationships among viruses in the *Chloroiridovirus* genus (Figure S10). The closest pairs formed a compact high-identity cluster comprising the isolates from lepidopteran hosts and IIV22/IIV22a (≥95%) confirming their close relationship at the nucleotide level. Comparisons with more distant IIVs (e.g., IIV25, IIV9) yielded ANI values around 80–81% whereas viruses in other genera all had values of ≤70%.

To clarify the structural relationships among the genomic sequences of our five isolates and nine reference IIV genomes, progressiveMauve [34] was used to identify 457 locally collinear blocks (LCBs). Of these, we recovered 54 core LCBs representing the conserved regions among our five sequenced isolates. Using AgIIV as a reference genome, these LCBs had a median size of 3028 bp and an N50 of 4520 bp (range: 1–9431 bp). Restricting the comparison to more closely related lineages resulted in blocks that tended to merge into longer segments, suggesting high collinearity.

The retention of these LCBs decreased with evolutionary distance. The SfIIV isolates (SfIIV-Arg, SfIIV-Ver and SfIIV-Chi), together with IIV22, IIV22a, IIV30B and IIV30C conserved 100% of the LCBs, while IIV25 and IIV9 retained 96.3%. More distant lineages, such as AMIV from an anopheline mosquito [44], retained 74.1%, and the most divergent ones (IIV3) and the IIVs from the *Iridovirus* genus (IIV6, and IIV31) retained only between 53.7% and 57.4%, indicating a loss of conserved genomic regions over time.

**Figure 7:**
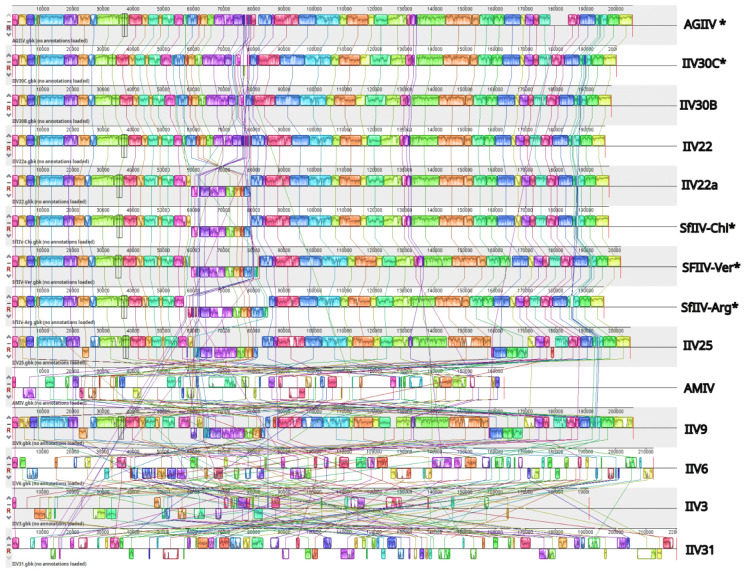
Whole genome alignment of genomic sequences of IIVs. Each genome displays several locally collinear blocks (LCBs) shown as different colored blocks. Lines of different colors connect related blocks with similar colors and patterns. An asterisk indicates the viruses sequenced in the present study.

The relative orientation of the LCBs compared to AGIIV revealed that the most closely related isolates, IIV30C and IIV30B, showed no inversions in their LCBs. However, other isolates exhibited higher inversion rates, with SfIIV-Chi and SfIIV-Ver having a 12.96% inversion rate and SfIIV-Arg showing a 16.7% inversion rate. The frequency of inversions in the reference genomes was proportional to their evolutionary distance. IIV25, AMIV, and IIV9 had inversion rates ranging from 21% to 23%, while the most divergent lineages (IIV31, IIV3, and IIV6) showed rates between 30% and 42% (Figure 7).

### 3.5. Phylogenetic Reconstruction

**Figure 8:**
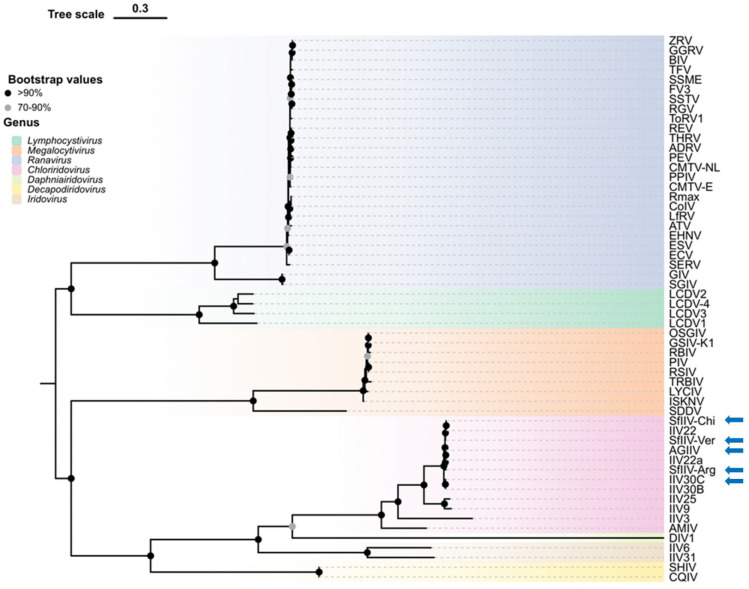
Phylogenetic analysis of the *Iridoviridae* family indicating the placement of the sequenced lepidopteran IIVs in the *Chloriridovirus* genus (pink hue). The analysis was based on the concatenated amino acid sequences of 26 core genes using the maximum likelihood method (ML-Tree). Bootstrap values from 1000 ultrafast replicates are shown at nodes; values ≥ 70% indicate statistically reliable relationships. Blue arrows indicate isolates that were sequenced in the present study. Virus name abbreviations are listed in Table S1. Core genes of the sequenced isolates from lepidopteran hosts are listed in Table S11.

The maximum-likelihood phylogeny inferred from the concatenation of amino acid sequences of 26 core genes across a broad reference panel of *Iridoviridae*, including representatives that infect vertebrates (*Alphairidovirinae*) and invertebrates (*Betairidovirinae*), revealed the clear subdivision of the family and all the recognized genera (Figure 8). Within this framework, all five lepidopteran isolates that we sequenced fall unambiguously within the *Chloriridovirus* genus with strong bootstrap support (≥99%), and with the members of the *Iridovirus* genus (IIV6, IIV31) and the other invertebrate genera, *Decapodiridovirus* (SHIV, CQIV) and *Daphniairidovirus* (DIV1), clearly separated.

**Figure 9:**
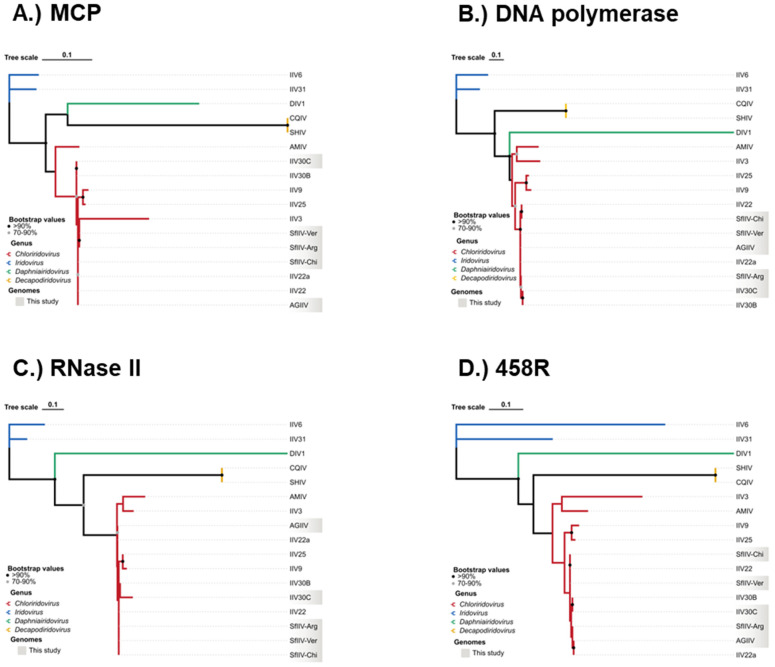
Phylogenetic trees obtained with amino acid sequence alignments corresponding to orthologs of the (**A**) major capsid protein (MCP), (**B**) DNA polymerase (DNApol), (**C**) RNase II and (**D**) 458R myristoylated membrane protein. Isolates sequenced in the present study are shaded gray.

Analysis of the four individual gene trees generated with orthologs of MCP, DNApol, RNase II, and the myristoylated membrane protein 458R essentially reproduced the monophyly of the *Chloriridovirus* genus and the isolates’ placement within this clade (Figure 9A–D). In all four markers, the closest neighbor of each of our isolates was consistently another chloriridovirus (e.g., IIV22, IIV22a, IIV30B/C, or a sister SfIIV isolate). Although the overall topological structure remained consistent, support values for terminal nodes varied between individual genes, ranging from ~30–100%, likely due to gene-specific rates of evolution and variation in the phylogenetic signal. However, the separation from more distant lineages was consistently maintained across all genes, with the most distant genomes typically being CQIV, DIV1, and IIV6 from the *Decapodiridovirus*, *Daphniairidovirus* and *Iridovirus* genera, respectively. These results support the value of these four markers for phylogenetic analyses. RNase II showed the highest congruence with DNApol (Spearman’s ρ = 0.970), whereas 458R was the marker with the highest relative variability compared to MCP (Spearman’s ρ = 0.876), although it remained globally congruent (Figure 9A–D). Minor topological discrepancies were observed at the “closest neighbor” level, which is expected given the modest genomic rearrangements and distribution of repetitive regions present in these genomes.

## 4. Discussion

In two field experiments in Chiapas, southern Mexico the prevalence of patent IIV disease was 0.86–1.05% and resulted in the collection of 63 patently infected *S. frugiperda* larvae. Patent IIV infection had not been observed in previous field experiments on SfMNPV efficacy as a biological insecticide in this region [45,46], and IIV infections were not observed in field studies that we conducted after the 1999 trials reported here. Despite the numerous researchers working on *S. frugiperda* as a pest of maize in North, Central and South America, there are no other reports of IIV infection of *S. frugiperda* except for a single report from Argentina [10]. This indicates that patent infection of this pest is rare across both continents.

Analysis of the first field experiment did not reveal any significant relationships between the prevalence of IIV infection and any of the other natural enemies infecting or parasitizing *S. frugiperda* larvae, perhaps due to the lower prevalence of IIV disease in the first experiment. In contrast, in the second field experiment, IIV disease was positively associated with the prevalence of SfMNPV infection. This may have resulted from a suppressed immune response in larvae from nucleopolyhedrovirus-treated plots, which might have favored IIV replication in individuals with sublethal nucleopolyhedrovirus infection. This idea is supported by the observation that sublethally infected insects suffer altered immune responses [47,48] and are more susceptible to superinfection than their healthy conspecifics [49]. A few *S. frugiperda* larvae were observed with clear signs of IIV-SfMNPV coinfection, i.e., iridescent blue larvae that were in the process of liquefaction, a known sign of lethal nucleopolyhedrovirus disease. Reports of replication of IIV in the presence of nucleopolyhedrovirus or cypovirus (*Reoviridae*) in dually infected cells have established that productive coinfection is possible in these viruses despite the nucleocytoplasmic and nuclear replication of each type of virus [50,51]. For this reason all IIV isolates were passaged in *G. mellonella* to eliminate possible contamination by SfMNPV in field-collected larvae.

The prevalence of IIV infection was negatively associated with parasitism by parasitoids despite clear evidence for parasitoid-mediated transmission of SfIIV-Chi in laboratory and field studies [9]. The reasons for this are unclear given that parasitism was common (5–32%, Table 2) in our experimental plots. However, tachinids, ectoparasitoids and egg-larval parasitoids such as *Chelonus insularis* are not believed to be involved in IIV transmission, which mainly involves larval endoparasitoids such as *Eiphosoma vitticolle* and *Ophion flavidus* (Ichneumonidae) that commonly attack *S. frugiperda* and other noctuids in this region [52,53].

No association was detected between IIV disease and nematode infection of *S. frugiperda* larvae. The species of nematode was not formally identified, but visual inspection was consistent with *Hexamermis* sp. (Nematoda: Mermithidae) which is known to infect *S. frugiperda* in Chiapas and elsewhere in Mexico and Central America [54,55]. *Hexamermis* sp. was also reported to be present in populations of *S. frugiperda* that were infected by the Argentinian isolate SfIIV-Arg [10]. This could be of interest as mermithid nematodes can transmit IIV in laboratory populations of dipteran larvae and terrestrial isopods [56,57]. However, the lack of evidence of nematode involvement in IIV disease in our field experiments underscores the scarcity of information on IIV transmission routes and the paucity of studies investigating the potential involvement of natural enemies as viral vectors.

Restriction endonuclease analysis is a rapid method for comparison of genetic diversity present across different virus isolates, although the popularity of this technique has fallen as modern sequencing techniques now provide genomic sequence information rapidly and at relatively low cost. In the present study, restriction enzyme analysis revealed that each of the patently infected *S. frugiperda* larvae was infected by a different strain of SfIIV-Chi evidenced by the presence of polymorphic restriction fragments in the HindIII and EcoRI profiles (Figure 2 and Figure S1). A very similar pattern of genetic diversity in IIV22/IIV22a isolates from infected individuals was observed previously in both patently and covertly infected larvae of the blackfly *Simulium variegatum* in the River Ystwyth, Wales, UK [4,58,59]. IIV25 was also present in blackfly larvae in this ecosystem [4,60]. IIV22, IIV22a and IIV30 are now considered strains of the virus species *Chloriridovirus simulium1*, whereas IIV25 is assigned to a separate virus species, *Chloriridovirus simulium2* [43]. Incidentally, the use of the IIV25 moniker was incorrectly assigned to the *Simulium* isolate from Wales by Piégu et al. [60], as IIV25 was originally isolated from a tipulid (Diptera) larva [61], although no sample remains available for testing as far as we are aware. Nonetheless, the adaptive significance of the strain-specific genetic variation observed in individuals infected by SfIIV-Chi and the mechanisms through which it is generated have yet to be clarified. Importantly, the examination of replicate restriction endonuclease profiles from SfIIV-Chi amplified in *S. frugiperda* and *G. mellonella* revealed that host species did not affect the distribution of HindIII or EcoRI restriction sites across the viral genome. Similarly, replicate digests of DNA amplified in different individual insects were identical and consistent across *G. mellonella* larvae indicating accurate replication of the original field-collected material and no evidence of viral cross-contamination among inoculated insects (Figure S3). This greatly increased our confidence in the use of the standard laboratory host, *G. mellonella*, for the amplification of the IIV isolates used in this study.

The five IIV genomes sequenced from lepidopteran hosts (Table 1) spanned 196.1 to 205.4 kbp, similar in length to the currently recognized IIVs (163.0–220.2 kbp, or exceptionally 288.8 kbp in the case of an IIV from *Daphnia*, DIV1) (Table S7). The GC content of the sequenced isolates (28.1–28.2%) was also typical of that observed in other IIVs (28.0–39.0% or exceptionally 47.9% in IIV3) [43]. ORF counts in our sequenced isolates (184–205 ORFs) were also consistent with that observed in other IIVs, which range from 150 ORFs in IIV-3 to 281 ORFs in DIV1 (Table S7), although variation in ORF number is likely a consequence of study-specific variation in the annotation strategy rather than biological divergence [18,62]. The observed variations in GC global skew, from a cytosine bias (SfIIV-Arg, IIV30C, AgIIV) to a guanine bias (SfIIV-Chi, SfIIV-Ver), are likely the results of shifts in mutational equilibrium, strand-specific selection pressure, or localized recombination events, rather than large-scale genomic inversions given the high structural collinearity observed across these genomes (Figure 7).

A salient feature of the sequenced genomes was the high prevalence of tandem repeat regions (66–88 per genome), a hallmark of iridoviruses that points to pronounced genomic plasticity [63,64]. Such repetitive architecture may facilitate rapid evolution and host adaptation across diverse invertebrate hosts, an issue of clear relevance given the broad host range of some IIV species. In parallel, the persistence of a large cohort of hypothetical proteins (43–52 per genome) highlights ongoing limitations in functional annotation for this family. Bridging this gap will require comparative genomics and functional characterization studies to resolve the range of the conserved and accessory functions of the IIV genic repertoire.

The synteny analysis identified a total of 457 LCBs across the five sequenced genomes, 54 of which were conserved among all isolates, indicating the presence of highly stable genomic regions. The strong positional conservation of these LCBs suggests that the overall genomic framework of this group of viruses was remarkably collinear but with clear examples of inversions in particular sections of the genome. Similar phenomena have been reported previously, such as the presence of inverted regions in IIV22 and IIV22a, or when comparing IIV9 with IIV22a or IIV30 [18,65]. The conserved LCBs detected in our assemblies are likely to harbor the core loci involved in virulence, replication, and gene regulation [66].

The AAI similarity analysis provided the first clear evidence that the IIV isolates from *S. frugiperda* and *A. gemmatalis* were highly similar to known members of the *Chloriridovirus* genus that also contains IIV30 (Figure 6). While our analysis used the ICTV genus demarcation threshold of 50% AAI for the *Iridoviridae*, recent comparative genomic analysis of 179 iridovirids suggested that a 70% threshold applied to the analysis of whole genomes may be more suitable for genus demarcation in this family [67]. Consequently, future taxonomic revisions may need to review these criteria as the diversity of IIVs continues to increase. AAI similarity values also indicated that the three SfIIV isolates and AgIIV were very closely related to IIV22 and IIV22a (>96% identity) and only slightly less similar to IIV30B and IIV30C (93.0–94.8% identity), leading us to conclude that the SfIIV isolates and AgIIV are closely related to the currently recognized species *Chloriridovirus simulium1*. This coincides with the 95% identity threshold specified by the ICTV for iridovirid species demarcation, although additional factors such as host range, G + C content, phylogenetic relatedness, genome co-linearity, disease manifestations are also employed to assign species status within each genus [43].

The maximum-likelihood phylogeny inferred from the concatenation of 26 core genes across a broad *Iridoviridae* panel comprising vertebrate and invertebrate representatives confirmed the family’s clear subdivision into *Alphairidovirinae* and *Betairidovirinae* [66], and resolved the major genera in line with current ICTV guidance [1,35,68]. Recent re-annotations have refined the number of core genes to 21–23 as the number of sequenced members of this family continues to increase [69], although the ICTV taxonomy of species currently remains based on the 26 core genes defined by Eaton et al. [35].

The consistently high bootstrap values (≥99%) across our isolates in the core gene analyses, together with the stable and congruent topologies recovered from the individual gene analyses, provide strong support for the taxonomic placement of our sequenced isolates (SfIIV-Chi, SfIIV-Arg, SfIIV-Ver, AgIIV and IIV30C) within the *Cloriridovirus* genus. Consistent with previous observations that *Chloriridovirus* forms a lineage distinct from members of the *Iridovirus* genus such as IIV6 and IIV31 [70,71], our results confirm the independent evolutionary position of this genus. Overall, the concordance among AAI similarity, core-gene phylogeny, and individual gene trees identified the SfIIV isolates, AgIIV and IIV30C as strains of *Chloriridovirus simulium1*.

Structural variations in IIV genomes can be considered intra- and inter-specific polymorphisms that reflect their evolutionary history. This genomic structure allows for the rearrangement of the genome without inactivating essential ORFs, which can lead to the establishment of viral lineages with distinct gene orders and host preferences [72]. In this respect, we were surprised to find that all our sequenced isolates were variants of the same virus species given the geographical separation and the differences in lifestyle and phylogenetic divergence of the host insects. As mentioned above, *Chloriridovirus simulium1* is known to infect aquatic *Simulium* larvae in the UK (IIV22, IIV22a) and a lepidopteran pest of field crops in the USA (IIV30B) [73], but this study extends the host range to include two additional lepidopteran hosts from Mexico and Argentina. The clear variation in gene order within the *Chloriridovirus* genus also echos that of the *Ranavirus* genus, although gene order tends to be more conserved among the strains of each species compared to genus-level variation [64,74]. Indeed, the members of the *Iridoviridae* are unusual in having such marked variation in genomic organization, which exceeds that of any other nucleocytoplasmic large DNA viruses [64].

These findings raise a series of questions as to why the virus infecting European blackflies has not diverged more strongly from strains that infect moths in the Americas. Also, why is it that a near-identical set of genes is required to achieve replication and transmission in aquatic dipteran larvae and terrestrial lepidopterans, given the markedly different physiology and environment of each type of host? Our findings also raise the possibility that strains of *Chloriridovirus simulium1* are freely circulating between lepidopterans and populations of blackflies or other dipteran hosts in the Americas or elsewhere. Uncharacterized IIV infections have been reported from blackfly larvae in southern Mexico [75] but these did not replicate in *G. mellonella* larvae so they have remained unstudied. If the large diversity of genes present in IIVs allows frequent multi-species infection across divergent invertebrate hosts, then studies on soil or aquatic invertebrate communities might reveal additional cases of broad host range relationships in other species of IIVs. This echoes with the extended host range of certain vertebrate iridovirid species such as *Ranavirus rana1*, typified by frog virus 3, that infects various species of fish, amphibians and reptiles across a range of different ecosystems [76]. Indeed, the major species within the *Ranavirus* genus all comprise multiple strains from a diversity of hosts so that host range is one of the key criteria used to define species staus in this genus [64].

The mechanisms that IIVs exploit to transmit across a variety of host species are also unclear. It is worth noting that many ichneumonid parasitoids parasitize a range of lepidopteran hosts, including soil-dwelling species such as *Agrotis* spp., which may offer a means to translocate virus from the soil invertebrate community to plant-feeding caterpillars. Whether and how IIV achieves translocation between aquatic and terrestrial hosts is less clear, although rare cases of parasitism of immature blackflies have been reported involving braconid and pteromalid endoparasitoids [77]. Similarly, nematodes such as *Hexamermis* sp. can infect a range of lepidopteran species [55] and may have the capacity to vector IIV particles among multiple host species, although we found no support for this idea from our field experiments.

With the exception of the decapod iridescent viruses SHIV and CQIV (species name *Decapodiridovirus litopenaeus1*), which infect multiple species of decapods of economic importance [78,79], current ongoing research on IIVs is generally stalled. Our findings highlight the somewhat fluid boundaries that define viral species and genera in the family *Iridoviridae*. Evident diversity was detected by restriction endonuclease analysis, AAI, and LCB analysis in SfIIV-Chi isolates collected at a single site over a short time period and across different hosts and geographic regions. Defining species boundaries in way that makes sense taxonomically and from an ecological perspective is likely to continue to be a challenge for these viruses. Notwithstanding such challenges, we envisage that finding IIV infection in a major agricultural pest, such as *S. frugiperda*, has the potential to inspire studies on the diversity, host–virus relationships and ecology of this charismatic but neglected group of pathogens.

## Figures and Tables

**Table 1:** Original host, geographic origin and source of IIV isolates used in the study.

Virus	Original Host	Origen	Source	Reference
IIV6	*Chilo suppressalis*	Japan	R. Webby, J. Kalmakoff	[15]
IIV9	*Wiseana cervinata*	New Zealand	IVEM Collection	[17]
IIV30C	*Helicoverpa zea*	USA	P. Christian	[20]
AgIIV	*Anticarsia gemmatalis*	Argentina	G. Kinard, C. Moore	[21,22]
SfIIV-Arg	*Spodoptera frugiperda*	Argentina	M.L. Vera	[10]
SfIIV-Chi	*Spodoptera frugiperda*	Chiapas, Mexico	T. Williams	This study
SfIIV-Ver	*Spodoptera frugiperda*	Veracruz, Mexico	M. López-Ortega	This study

**Table 2:** Field sampling of *Spodoptera frugiperda* larvae in two field experiments performed in Chiapas state, Mexico.

Treatment	Number of Maize Plots Sampled	Sample Day	Total Number of Larvae Collected	IIV Infected ± SE (%) [n]	SfMNPV Infected ± SE (%) [n]	Parasitism by Parasitoids ± SE (%) [n]	Infection by Nematodes ± SE (%) [n]
Experiment 1							
Virus plots	53	2	1273	0.24 ± 0.14 [3]	10.05 ± 0.84 [128]	24.98 ± 1.21 [318]	6.36 ± 0.68 [81]
	54	5	874	0.23 ± 0.16 [2]	9.84 ± 1.01 [86]	31.46 ± 1.57 [275]	3.66 ± 0.64 [32]
	54	10	662	2.42 ± 0.60 [16]	7.55 ± 1.03 [50]	16.46 ± 1.44 [109]	8.91 ± 1.11 [59]
Control plots	18	2	456	0.00 [0]	0.00 [0]	30.92 ± 2.16 [141]	2.63 ± 0.75 [12]
	18	5	441	0.00 [0]	0.00 [0]	31.97 ± 2.22 [141]	7.71 ± 1.27 [34]
	18	10	248	5.24 ± 1.42 [13]	0.40 ± 0.40 [1]	15.73 ± 2.31 [39]	10.08 ± 1.91 [25]
							
Experiment 2							
Virus plots	54	2	748	1.07 ± 0.38 [8]	33.96 ± 1.73 [254]	26.34 ± 1.61 [197]	2.01 ± 0.51 [15]
	54	5	761	1.05 ± 0.37 [8]	22.08 ± 1.50 [168]	18.79 ± 1.42 [143]	9.59 ± 1.07 [73]
	54	10	312	0.64 ± 0.45 [2]	6.73 ±1.42 [21]	12.18 ± 1.85 [38]	10.90 ± 1.76 [34]
Control plots	27	2	451	0.22 ± 0.22 [1]	0.44 ± 0.31 [2]	25.28 ± 2.05 [114]	1.55 ± 0.58 [7]
	27	5	324	0.93 ± 0.53 [3]	0.31 ± 0.31 [1]	19.14 ± 2.19 [62]	5.86 ± 1.31 [19]
	27	10	154	4.55 ± 1.68 [7]	1.30 ± 0.91 [2]	5.19 ±1.79 [8]	12.99 ± 2.71 [20]

Means (±binomial SE) are based on larvae sampled from 18 to 54 plots of whorl-stage maize. Plots were sampled at 2, 5 or 10 days after the application of SfMNPV occlusion body suspensions or water (control). *Spodoptera frugiperda* larvae found on 10 maize plants per plot were taken to the laboratory, individually placed in cups with semi-synthetic diet and monitored daily for infection. Value [n] indicates the number of affected larvae observed in each class. Binomial standard errors SE were calculated as √[p(1-p)/t], where p = proportion of infected insects and t = total number of larvae sampled.

**Table 3:** Comparison of IIV genome attributes.

Genome	Length (bp)	GC (%)	ORFs	Hypothetical Proteins	Repeats	GC Skew (%)	Cumulative GC Skew Amplitude (%)	ENA Accession no.
SfIIV-Chi	197,661	28.1	185	43	87	3.43	714.09	ERZ28593071
SfIIV-Ver	201,630	28.2	184	44	66	3.52	729.18	ERZ28593070
SfIIV-Arg	196,053	28.1	196	50	80	−3.52	701.61	ERZ28593069
IIV30C	200,215	28.2	209	53	83	−2.98	621.89	ERZ28593068
AgIIV	205,445	28.1	195	47	85	−3.10	674.81	ERZ28593072

GC skew ((G − C)/(G + C)) × 100 and cumulative GC skew amplitude (1 kb window, 500 bp step) are included as indicators of strand-specific compositional asymmetry.

## Data Availability

The original contributions presented in this study are included in the article and Supplementary Material Files. Further inquiries can be directed to the corresponding authors.

## Supplementary Materials

The following supporting information can be downloaded at: https://www.mdpi.com/article/10.3390/v18010031/s1, File_S1.xlsx Original data from field experiments; File_S2.xlsx In silico digestion fragment lengths with HindIII and EcoRI; File_S3.zip Genome sequence data files in fasta and gbk format; Figure S1. EcoRI restriction fragment length polymorphism of SfIIV isolates from Chiapas; Figure S2. EcoRI restriction fragment length polymorphism of IIV isolates from lepidopteran hosts; Figure S3. Replicate restriction endonuclease digests of IIV isolates; Figure S4. In silico digestion of IIV genomes from lepidopteran hosts; Figure S5. Annotation of linear genome of SfIIV-Chi; Figure S6. Annotation of linear genome of SfIIV-Ver; Figure S7. Annotation of linear genome of SfIIV-Arg; Figure S8. Annotation of linear genome of AgIIV; Figure S9. Annotation of linear genome of IIV30C; Figure S10. Average Nucleotide Identity (ANI) heatmap and hierarchical clustering of IIV genomes; Table S1. Genomic characteristics of species in the family Iridoviridae with their common name and abbreviation; Table S2. Slope and odds ratio for IIV infection in relation to SfMNPV infection, nematode infection and parasitism by parasitoids in field experiments performed in Chiapas state, Mexico; Table S3. Sequencing statistics and assembly metrics of IIV genomes; Table S4. ORF functional categories summary for IIVs from lepidopteran hosts; Table S5. Hypothetical proteins and transmembrane domains (TM) present in IIV genomes; Table S6. SfIIV-Chi genome annotation; Table S7. SfIIV-Ver genome annotation; Table S8. SfIIV-Arg genome annotation; Table S9. AgIIV genome annotation; Table S10. IIV30C genome annotation; Table S11. Core genes among the sequenced IIVs from lepidopteran hosts.

## References

[1] Chinchar V.G., Hick P., Ince I.A., Jancovich J.K., Marschang R., Qin Q., Subramaniam K., Waltzek T.B., Whittington R., Williams T. (2017). ICTV Report Consortium. ICTV Virus Taxonomy Profile: *Iridoviridae*. J. Gen. Virol..

[2] İnce I.A. (2021). Iridoviruses of invertebrates (*Iridoviridae*). Encyclopedia of Virology.

[3] Schnitzler P., Soltau J.B., Fischer M., Reisner H., Scholz J., Delius H., Darai G. (1987). Molecular cloning and physical mapping of the genome of insect iridescent virus type 6: Further evidence for circular permutation of the viral genome. Virology.

[4] Williams T. (1995). Patterns of covert infection by invertebrate pathogens: Iridescent viruses of blackflies. Mol. Ecol..

[5] Duffield K.R., Hunt J., Sadd B.M., Sakaluk S.K., Oppert B., Rosario K., Behle R.W., Ramirez J.L. (2021). Active and covert infections of cricket iridovirus and Acheta domesticus densovirus in reared *Gryllodes sigillatus* crickets. Front. Microbiol..

[6] Chinchar V.G., Hyatt A., Miyazaki T., Williams T. (2009). Iridoviridae: Poor viral relations no longer. Curr. Top. Microbiol. Immunol..

[7] Tay W.T., Meagher R.L., Czepak C., Groot A.T. (2023). *Spodoptera frugiperda*: Ecology, evolution, and management options of an invasive species. Annu. Rev. Entomol..

[8] Williams T., Hernández O. (2006). Costs of cannibalism in the presence of an iridovirus pathogen of *Spodoptera frugiperda*. Ecol. Entomol..

[9] López M., Rojas J.C., Vandame R., Williams T. (2002). Parasitoid-mediated transmission of an iridescent virus. J. Invertebr. Pathol..

[10] Vera M.L., Valverde L., Popich S.B., Ajmat de Toledo Z.D. (1995). Evaluación preliminar de los enemigos naturales de *Spodoptera frugiperda* (J.E. Smith) (Lepidoptera: Noctuidae) en Tucumán, Argentina. Acta Entomol. Chilena.

[11] Williams T., Miller L.K., Ball L.A. (1998). Invertebrate iridescent viruses. The Insect Viruses.

[12] İnce İ.A., Özcan O., Ilter-Akulke A.Z., Scully E.D., Özgen A. (2018). Invertebrate iridoviruses: A glance over the last decade. Viruses.

[13] Kenis M., Benelli G., Biondi A., Calatayud P.A., Day R., Desneux N., Harrison R.D., Kriticos D., Rwomushana I., van den Berg J. (2023). Invasiveness, biology, ecology, and management of the fall armyworm, *Spodoptera frugiperda*. Entomol. Gen..

[14] Seabold S., Perktold J. Statsmodels: Econometric and statistical modeling with python. Proceedings of the 9th Python in Science Conference.

[15] Jakob N.J., Muller K., Bahr U., Darai G. (2001). Analysis of the first complete DNA sequence of an invertebrate iridovirus: Coding strategy of the genome of Chilo iridescent virus. Virology.

[16] Williams T., Cory J.S. (1994). Proposals for a new classification of iridescent viruses. J. Gen. Virol..

[17] Wong C.K., Young V.L., Kleffmann T., Ward V.K. (2011). Genomic and proteomic analysis of invertebrate iridovirus type 9. J. Virol..

[18] Piégu B., Guizard S., Spears T., Cruaud C., Couloux A., Bideshi D.K., Federici B.A., Bigot Y. (2014). Complete genome sequence of invertebrate iridovirus IIV30 isolated from the corn earworm, *Helicoverpa zea*. J. Invertebr. Pathol..

[19] Sieburth P.J., Carner G.R. (1987). Infectivity of an iridescent virus for larvae of *Anticarsia gemmatalis* (Lepidoptera: Noctuidae). J. Invertebr. Pathol..

[20] Stadelbacher E.A., Adams J.R., Faust R.M., Tompkins G.J. (1978). An iridescent virus of the bollworm *Heliothis zea* (Lepidoptera: Noctuidae). J. Invertebr. Pathol..

[21] Kinard G.R., Barnett O.W., Carner G.R. (1995). Characterization of an iridescent virus isolated from the velvetbean caterpillar, *Anticarsia gemmatalis*. J. Invertebr. Pathol..

[22] Williams T. (1994). Comparative studies of iridoviruses: Further support for a new classification. Virus Res..

[23] Wick R. (2017). Filtlong. https://github.com/rrwick/Filtlong.

[24] Bolger A.M., Lohse M., Usadel B. (2014). Trimmomatic: A flexible trimmer for Illumina sequence data. Bioinformatics.

[25] Kolmogorov M., Yuan J., Lin Y., Pevzner P.A. (2019). Assembly of long, error-prone reads using repeat graphs. Nature Biotechnol..

[26] Wick R.R., Judd L.M., Gorrie C.L., Holt K.E. (2017). Unicycler: Resolving bacterial genome assemblies from short and long sequencing reads. PLoS Comput. Biol..

[27] Gurevich A., Saveliev V., Vyahhi N., Tesler G. (2013). QUAST: Quality assessment tool for genome assemblies. Bioinformatics.

[28] Seemann T. (2014). Prokka: Rapid prokaryotic genome annotation. Bioinformatics.

[29] Cantalapiedra C.P., Hernández-Plaza A., Letunic I., Bork P., Huerta-Cepas J. (2021). EggNOG-mapper v2: Functional annotation, orthology assignments, and domain prediction at the metagenomic scale. Mol. Biol. Evol..

[30] Fu P., Wu Y., Zhang Z., Qiu Y., Wang Y., Peng Y. (2024). VIGA: A one-stop tool for eukaryotic virus identification and genome assembly from next-generation-sequencing data. Brief. Bioinform..

[31] Hallgren J., Tsirigos K.D., Pedersen M.D., Almagro Armenteros J.J., Marcatili P., Nielsen H., Krogh A., Winther O. (2022). DeepTMHMM predicts alpha and beta transmembrane proteins using deep neural networks. bioRxiv.

[32] Stothard P., Wishart D.S. (2005). Circular genome visualization and exploration using CGView. Bioinformatics.

[33] Parks D.H. CompareM v.0.0.23. https://github.com/donovan-h-parks/CompareM.

[34] Darling A.E., Mau B., Perna N.T. (2010). ProgressiveMauve: Multiple genome alignment with gene gain, loss and rearrangement. PLoS ONE.

[35] Eaton H.E., Metcalf J., Penny E., Tcherepanov V., Upton C., Brunetti C.R. (2007). Comparative genomic analysis of the family Iridoviridae: Re-annotating and defining the core set of iridovirus genes. Virol. J..

[36] Emms D.M., Kelly S. (2019). OrthoFinder: Phylogenetic orthology inference for comparative genomics. Genome Biol..

[37] Steinegger M., Söding J. (2017). MMseqs2 enables sensitive protein sequence searching for the analysis of massive data sets. Nat. Biotechnol..

[38] Katoh K., Standley D.M. (2013). MAFFT multiple sequence alignment software version 7: Improvements in performance and usability. Mol. Biol. Evol..

[39] Capella-Gutiérrez S., Silla-Martínez J.M., Gabaldón T. (2009). t TrimAl: A tool for automated alignment trimming in large-scale phylogenetic analyses. Bioinformatics.

[40] Minh B.Q., Schmidt H.A., Chernomor O., Schrempf D., Woodhams M.D., Von Haeseler A., Lanfear R. (2020). IQ-TREE 2: New models and efficient methods for phylogenetic inference in the genomic era. Mol. Biol. Evol..

[41] (2025). Chiplot Online Graphical Software. https://www.chiplot.online.

[42] Scipy 2023. Scipy v. 1.11.1 Online Algorithms for Scientific Computing in Python. https://pypi.org/project/scipy/1.11.1/.

[43] ICTV 2025. International Committee on Virus Taxonomy. Family *Iridoviridae*. https://ictv.global/report/chapter/iridoviridae/iridoviridae.

[44] Huang Y., Li S., Zhao Q., Pei G., An X., Guo X., Zhou H., Zhang Z., Zhang J., Tong Y. (2015). Isolation and characterization of a novel invertebrate iridovirus from adult *Anopheles minimus* (AMIV) in China. J. Invertebr. Pathol..

[45] Williams T., Goulson D., Caballero P., Cisneros J., Martínez A.M., Chapman J.W., Roman D.X., Cave R.D. (1999). Evaluation of a baculovirus bioinsecticide for small scale maize growers in Latin America. Biol. Control.

[46] Martínez A.M., Goulson D., Chapman J.W., Caballero P., Cave R.D., Williams T. (2000). Is it feasible to use optical brightener technology with a baculovirus bioinsecticide for resource-poor maize farmers in Mesoamerica?. Biol. Control.

[47] Jakubowska A.K., Vogel H., Herrero S. (2013). Increase in gut microbiota after immune suppression in baculovirus-infected larvae. PLoS Pathog..

[48] Cabodevilla O., Villar E., Virto C., Murillo R., Williams T., Caballero P. (2011). Intra- and intergenerational persistence of an insect nucleopolyhedrovirus: Adverse effects of sublethal disease on host development, reproduction, and susceptibility to superinfection. Appl. Environ. Microbiol..

[49] Williams T., Virto C., Murillo R., Caballero P. (2017). Covert infection of insects by baculoviruses. Front. Microbiol..

[50] Kimura M., McIntosh A.H., Kurstak E., Maramorosch K. (1976). Dual infection of the *Trichoplusia ni* cell line with the Chilo iridescent virus (CIV) and Autographa californica nuclear polyhedrosis virus. Invertebrate Tissue Culture: Applications in Medicine, Biology, and Agriculture.

[51] Arella M., Devauchelle G., Belloncik S. (1983). Dual infection of a lepidopterean cell line with the cytoplasmic polyhedrosis virus (CPV) and the Chilo iridescent virus (CIV). Ann. L’institut Pasteur/Virol..

[52] Armenta R., Martínez A.M., Chapman J.W., Magallanes R., Goulson D., Caballero P., Cave R.D., Cisneros J., Valle J., Castillejos V. (2003). Impact of a nucleopolyhedrovirus bioinsecticide and selected synthetic insecticides on the abundance of insect natural enemies on maize in Southern Mexico. J. Econ. Entomol..

[53] Molina-Ochoa J., Carpenter J.E., Lezama-Gutiérrez R., Foster J.E., González-Ramírez M., Angel-Sahagún C.A., Farías-Larios J. (2004). Natural distribution of hymenopteran parasitoids of *Spodoptera frugiperda* (Lepidoptera: Noctuidae) larvae in Mexico. Fl. Entomol..

[54] Molina-Ochoa J., Lezama-Gutierrez R., Gonzalez-Ramirez M., Lopez-Edwards M., Rodriguez-Vega M.A., Arceo-Palacios F. (2003). Pathogens and parasitic nematodes associated with populations of fall armyworm (Lepidoptera: Noctuidae) larvae in Mexico. Fl. Entomol..

[55] Ruiz-Nájera R.E., Ruiz-Estudillo R.A., Sánchez-Yáñez J.M., Molina-Ochoa J., Skoda S.R., Coutiño-Ruiz R., Pinto-Ruiz R., Guevara-Hernández F., Foster J.E. (2013). Occurrence of entomopathogenic fungi and parasitic nematodes on *Spodoptera frugiperda* (Lepidoptera: Noctuidae) larvae collected in central Chiapas, México. Fl. Entomol..

[56] Mullens B.A., Velten R.K., Federici B.A. (1999). Iridescent virus infection in *Culicoides variipennis sonorensis* and interactions with the mermithid parasite *Heleidomermis magnapapula*. J. Invertebr. Pathol..

[57] Hess R.T., Poinar G.O. (1985). Iridoviruses infecting terrestrial isopods and nematodes. Curr. Top. Microbiol. Immunol..

[58] Williams T., Cory J.S. (1993). DNA restriction fragment polymorphism in iridovirus isolates from individual blackflies (Diptera: Simuliidae). Med. Vet. Entomol..

[59] Williams T. (1993). Covert iridovirus infection of blackfly larvae. Proc. R. Soc. B.

[60] Piégu B., Guizard S., Spears T., Cruaud C., Couloux A., Bideshi D.K., Federici B.A., Bigot Y. (2014). Complete genome sequence of invertebrate iridovirus IIV-25 isolated from a blackfly larva. Arch. Virol..

[61] Elliott R.M., Lescott T., Kelly D.C. (1977). Serological relationships of an iridescent virus (type 25) recently isolated from *Tipula* sp. with two other iridescent viruses (types 2 and 22). Virology.

[62] Yesilyurt A., Demirbag Z., van Oers M.M., Nalcacioglu R. (2020). Conserved motifs in the invertebrate iridescent virus 6 (IIV6) genome regulate virus transcription. J. Invertebr. Pathol..

[63] Toenshoff E.R., Fields P.D., Bourgeois Y.X., Ebert D. (2018). The end of a 60-year riddle: Identification and genomic characterization of an iridovirus, the causative agent of white fat cell disease in zooplankton. G3 Gen. Genom. Genet..

[64] Waltzek T.B., Subramaniam K., Jancovich J.K., Gray M.J., Chinchar V.G. (2024). Ranavirus taxonomy and phylogeny. Ranaviruses: Emerging Pathogens of Ectothermic Vertebrates.

[65] Piégu B., Guizard S., Yeping T., Cruaud C., Couloux A., Bideshi D.K., Federici B.A., Bigot Y. (2014). Complete genome sequence of invertebrate iridovirus IIV22A, a variant of IIV22, isolated originally from a blackfly larva. Stand. Gen. Sci..

[66] Deng Z., Wang J., Zhang W., Geng Y., Zhao M., Gu C., Fu L., He M., Xiao Q., Xiao W. (2021). The insights of genomic synteny and codon usage preference on genera demarcation of Iridoviridae family. Front. Microbiol..

[67] Zhao R., Gu C., Zou X., Zhao M., Xiao W., He M., He L., Yang Q., Geng Y., Yu Z. (2022). Comparative genomic analysis reveals new evidence of genus boundary for family *Iridoviridae* and explores qualified hallmark genes. Comput. Struct. Biotechnol. J..

[68] Ballard D.R., Davis A.J., Fuller R.B., Garner A.R., Mileham A.D., Serna J.D., Brue D.E., Harding C.M., Dodgen C.D., Culpepper W. (2020). An examination of the Iridovirus core genes for reconstructing Ranavirus phylogenies. Facets.

[69] Canuti M., Large G., Verhoeven J.T.P., Dufour S.C. (2022). A novel iridovirus discovered in deep-sea carnivorous sponges. Viruses.

[70] Piégu B., Guizard S., Yeping T., Cruaud C., Asgari S., Bideshi D.K., Federici B.A., Bigot Y. (2014). Genome sequence of a crustacean iridovirus, IIV31, isolated from the pill bug, *Armadillidium vulgare*. J. Gen. Virol..

[71] Piégu B., Asgari S., Bideshi D., Federici B.A., Bigot Y. (2015). Evolutionary relationships of iridoviruses and divergence of ascoviruses from invertebrate iridoviruses in the superfamily Megavirales. Mol. Phylog. Evol..

[72] Williams T., Barbosa-Solomieu V., Chinchar V.G. (2005). A decade of advances in iridovirus research. Adv. Virus Res..

[73] Williams T. (2008). Natural invertebrate hosts to iridoviruses (Iridoviridae). Neotrop. Entomol..

[74] Jancovich J.K., Zhang Q.Y., Chinchar V.G., Gray M.J., Chinchar V.G. (2024). Ranavirus replication: New studies provide answers to old questions. Ranaviruses: Emerging Pathogens of Ectothermic Vertebrates.

[75] Hernández O., Maldonado G., Williams T. (2000). An epizootic of patent iridescent virus disease in multiple species of blackflies in Chiapas, Mexico. Med. Vet. Entomol..

[76] Marschang R.E., Meddings J.I., Waltzek T.B., Hick P., Allender M.C., Wirth W., Duffus A.L.J., Gray M.J., Chinchar V.G. (2024). Ranavirus distribution and host range. Ranaviruses: Emerging Pathogens of Ectothermic Vertebrates.

[77] Williams T., Cory J.S. (1991). A new record of hymenopterous parasitism of an immature blackfly (Diptera: Simuliidae). Entomol. Gaz..

[78] Liao X., He J., Li C. (2022). Decapod iridescent virus 1: An emerging viral pathogen in aquaculture. Rev. Aquacult..

[79] Hao J., Jie Y., Lu Z., Ye T., Meng J., Liu C., Yan J., Zheng Y., Dong Z., Gu Z. (2025). Genomic characterization of Decapod iridescent virus 1 (DIV1) and its host immune responses in *Macrobrachium rosenbergii*. Dev. Comp. Immunol..

