# Supplementary material for: Invertebrate Iridescent Viruses (Iridoviridae) from the Fall Armyworm, Spodoptera frugiperda
Source: Viruses. 2025 Dec 24;18(1):31. doi: 10.3390/v18010031 (PMC12846554; doi:10.3390/v18010031)

Figure S1. EcoRI restriction fragment length polymorphism of SfIIV isolates from Chiapas. Twelve isolates from individual infected *Spodoptera frugiperda* larvae are labeled A-L. The position of weak bands (<1.5 kb) is indicated with asterisks. Molecular size markers are lambda phage-HindIII (M1) and New England Biolabs 1 Kb ladder (M2).

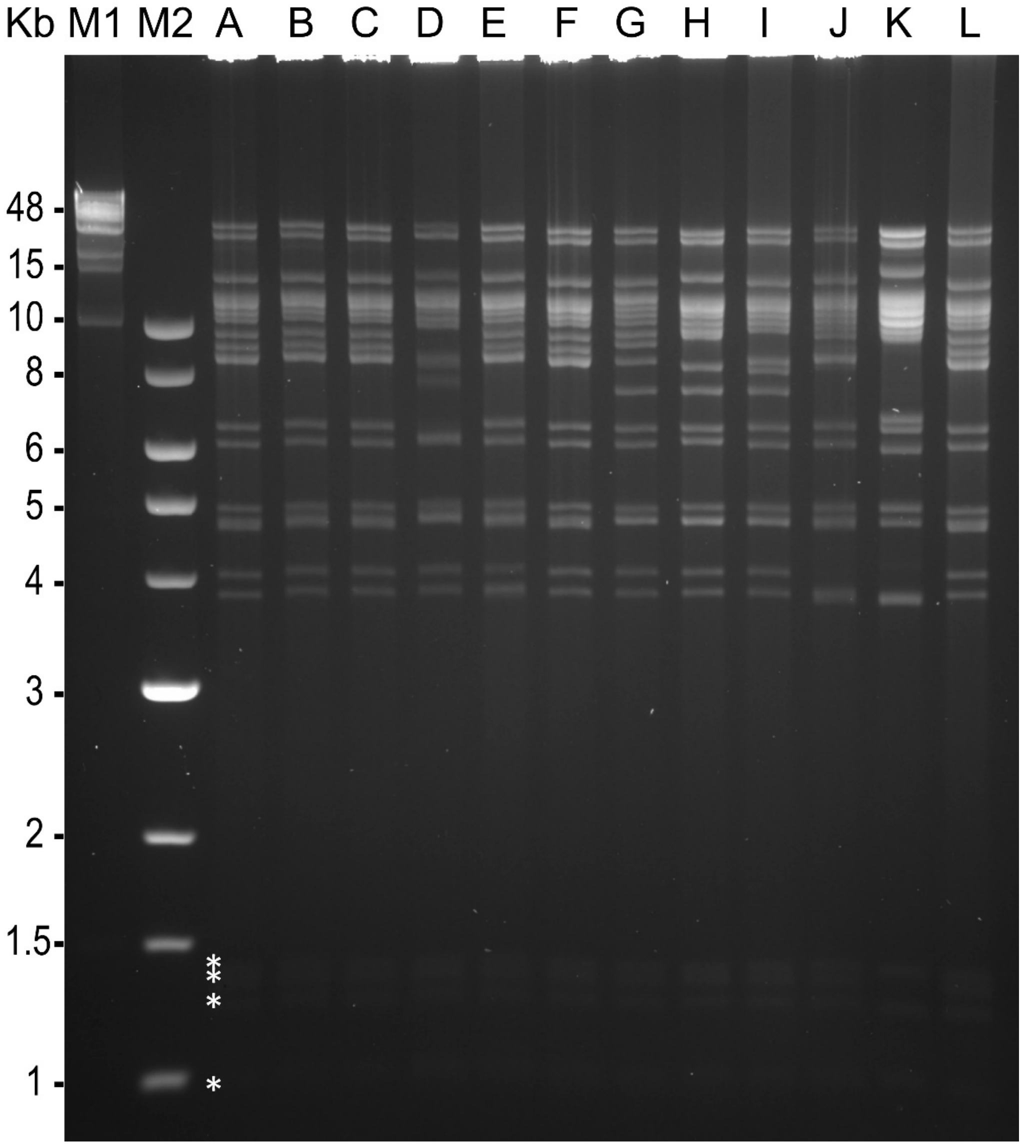

Supplement: Supplementary file 1 [file viruses-18-00031-s001.zip › Fig_S1.pdf]
