# Supplementary material for: Invertebrate Iridescent Viruses (Iridoviridae) from the Fall Armyworm, Spodoptera frugiperda
Source: Viruses. 2025 Dec 24;18(1):31. doi: 10.3390/v18010031 (PMC12846554; doi:10.3390/v18010031)
Supplement: Supplementary file 1 [file viruses-18-00031-s001.zip › Fig_S10.pdf]

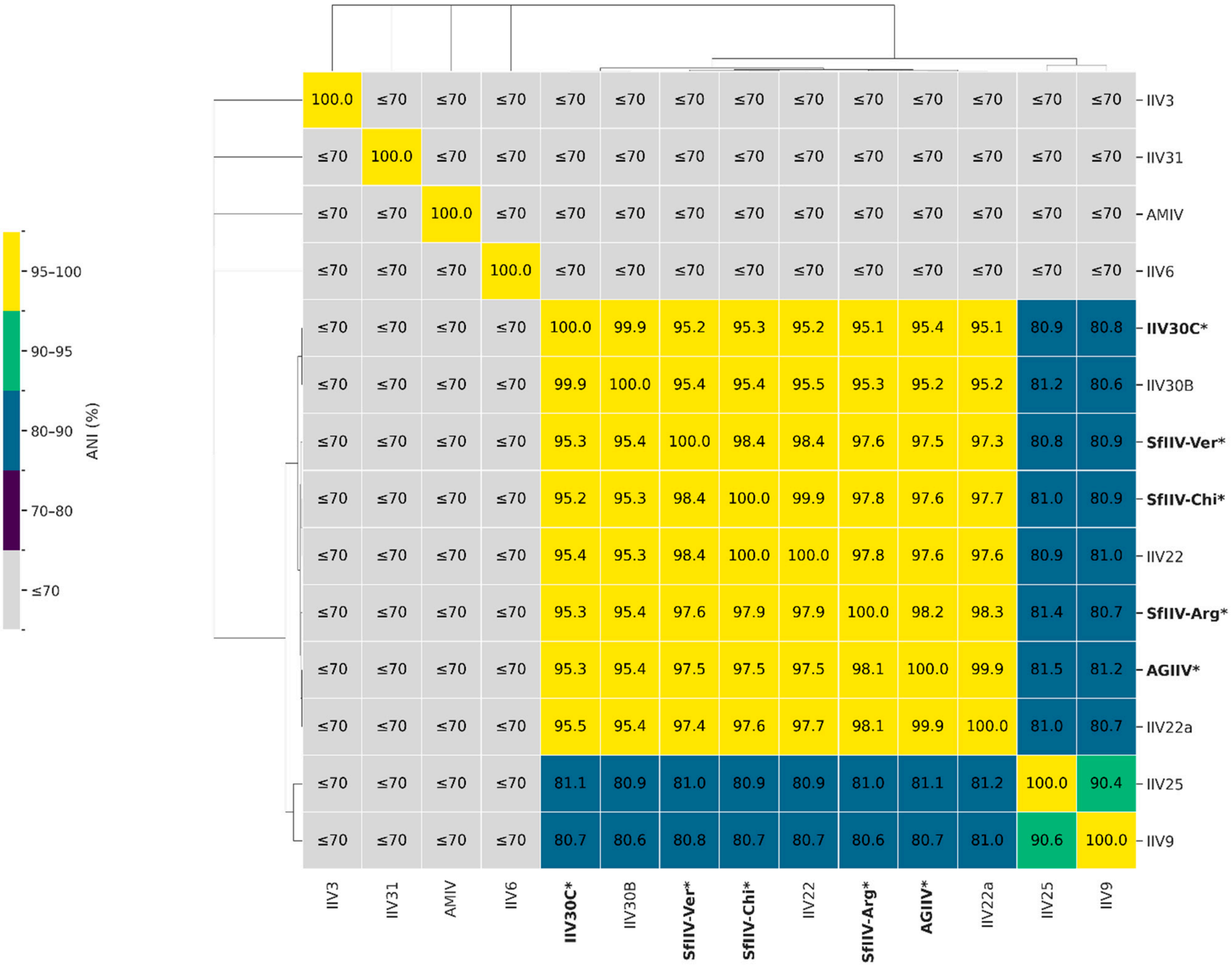

Figure S10. Average Nucleotide Identity (ANI) heatmap and hierarchical clustering of IIV genomes. Values shown in gray (<70%) are viruses assigned to genera other than the genus *Chloriridovirus* (colored squares). Viruses sequenced in the present study are shown in bold type with an asterisk.
