# Supplementary material for: Invertebrate Iridescent Viruses (Iridoviridae) from the Fall Armyworm, Spodoptera frugiperda
Source: Viruses. 2025 Dec 24;18(1):31. doi: 10.3390/v18010031 (PMC12846554; doi:10.3390/v18010031)

Figure S2. EcoRI restriction fragment length polymorphism of IIV isolates from lepidopteran hosts. Molecular size markers are lambda phage-HindIII (M1) and New England Biolabs 1 Kb ladder (M2).

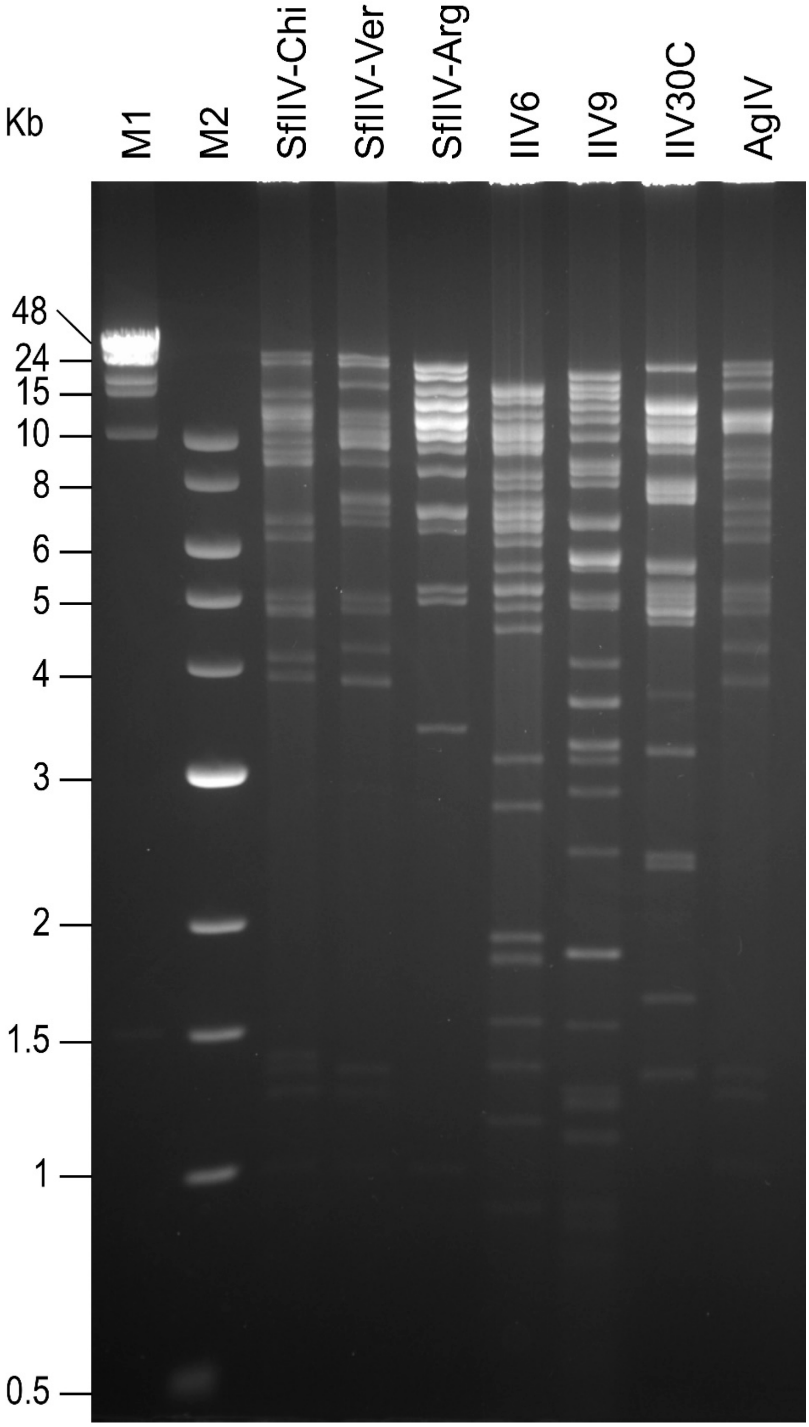

Supplement: Supplementary file 1 [file viruses-18-00031-s001.zip › Fig_S2.pdf]
