# Supplementary material for: Invertebrate Iridescent Viruses (Iridoviridae) from the Fall Armyworm, Spodoptera frugiperda
Source: Viruses. 2025 Dec 24;18(1):31. doi: 10.3390/v18010031 (PMC12846554; doi:10.3390/v18010031)

Figure S3. Replicate restriction endonuclease digests of IIV isolates. Genomic DNAs were treated with (A) HindIII or (B) EcoRI. Lanes A-C, SfIIV-Chi amplified in *S. frugiperda* larvae; Lanes D-F, SfIIV-Chi amplified in *G. mellonella* larvae; lanes G-I, SfIIV-Ver amplified in *G. mellonella* larvae; SfIIV-Arg amplified in *G. mellonella* larvae. In all cases, the three replicate samples were purified from three different infected larvae.

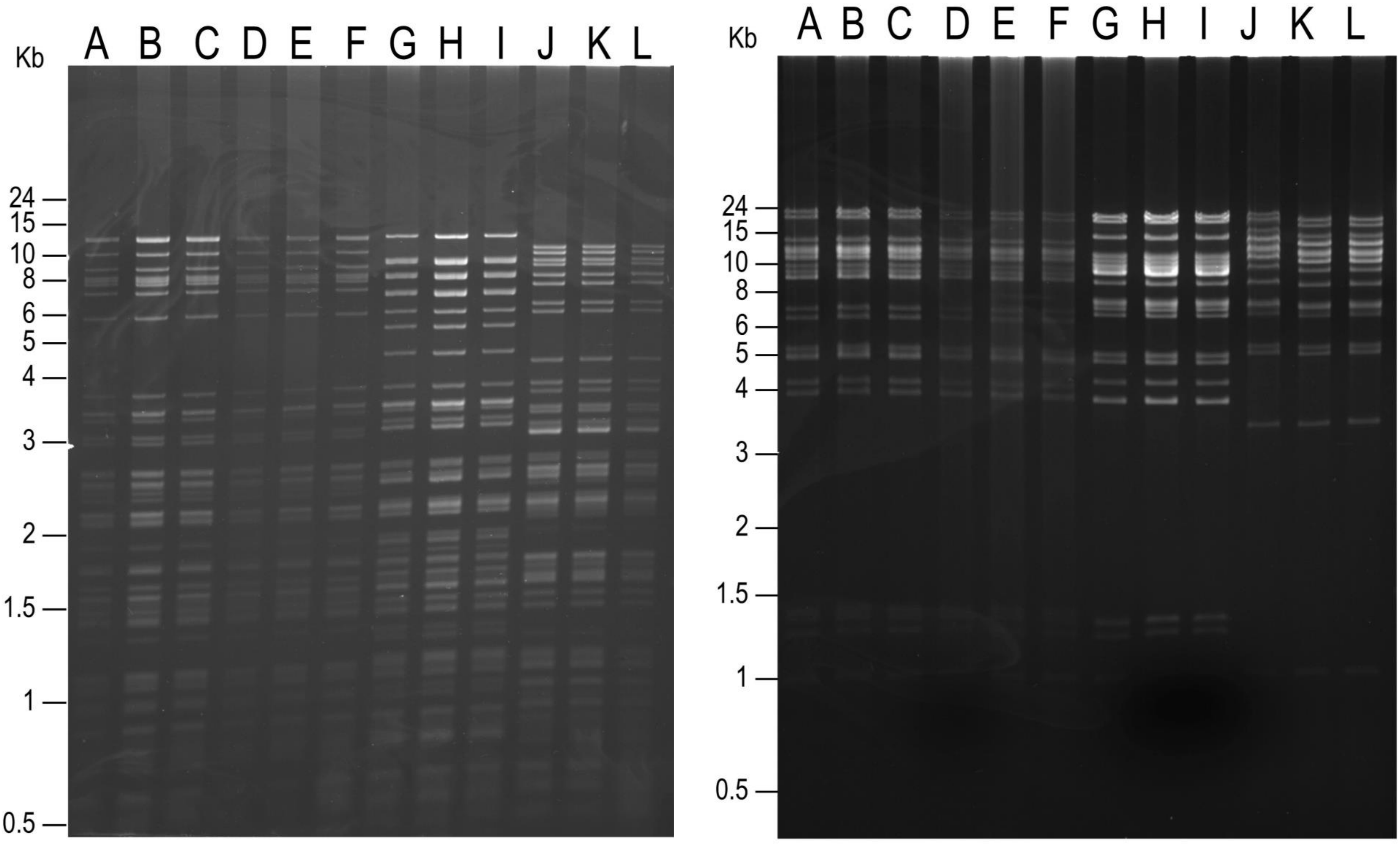

Supplement: Supplementary file 1 [file viruses-18-00031-s001.zip › Fig_S3.pdf]
