# Supplementary material for: Invertebrate Iridescent Viruses (Iridoviridae) from the Fall Armyworm, Spodoptera frugiperda
Source: Viruses. 2025 Dec 24;18(1):31. doi: 10.3390/v18010031 (PMC12846554; doi:10.3390/v18010031)

Figure S4. In silico digestion of IIV genomes from lepidopteran hosts. Genomes were treated with (A) HindIII or (B) EcoRI. Values on left indicate molecular marker sizes (Kb).

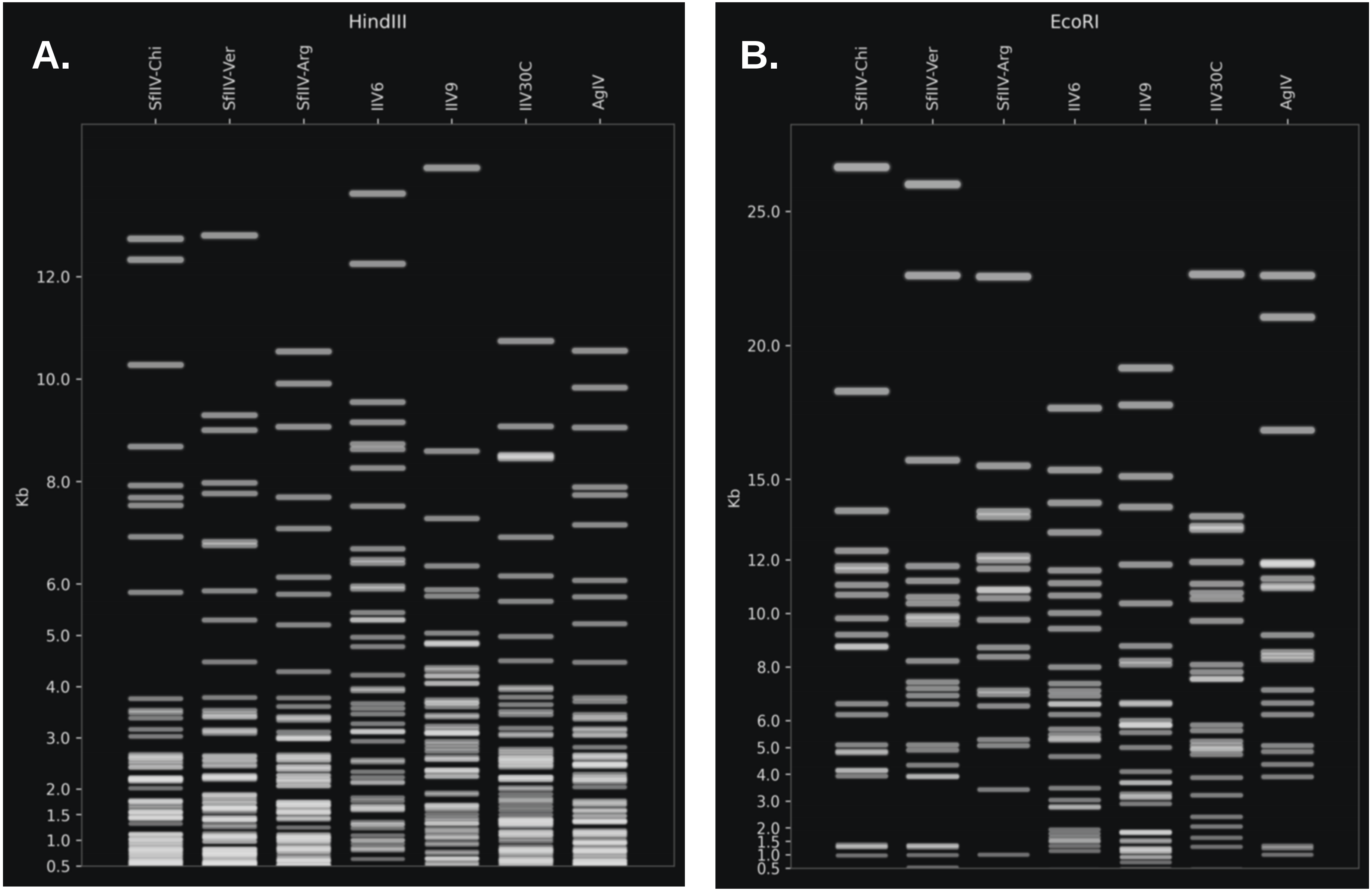

Supplement: Supplementary file 1 [file viruses-18-00031-s001.zip › Fig_S4.pdf]
