# Supplementary material for: Invertebrate Iridescent Viruses (Iridoviridae) from the Fall Armyworm, Spodoptera frugiperda
Source: Viruses. 2025 Dec 24;18(1):31. doi: 10.3390/v18010031 (PMC12846554; doi:10.3390/v18010031)
Supplement: Supplementary file 1 [file viruses-18-00031-s001.zip › Table_S1.pdf]

Table S1. Genomic characteristics of species in the family *Iridoviridae* with their common name and abbreviation

Genus	Species	Virus common name	Genome length (bp)	GC%	ORFs	Accession	Abbreviation
Lymphocystivirus	*Lymphocystivirus micropogonias1*	Lymphocystis disease virus 4	211086	26.03	145	MN803438	LCDV-4
Lymphocystivirus	*Lymphocystivirus paralichthys1*	Lymphocystis disease virus 2	186250	27.23	144	AY380826	LCDV2
Lymphocystivirus	*Lymphocystivirus platichthys1*	Lymphocystis disease virus 1	102653	29.07	110	L63545	LCDV1
Lymphocystivirus	*Lymphocystivirus sparus1*	Lymphocystis disease virus 3	208501	33.01	152	KX643370	LCDV3
Megalocytivirus	*Megalocytivirus lates1*	Scale drop disease virus	124244	36.86	135	KR139659	SDDV
Megalocytivirus	*Megalocytivirus pagrus1*	Infectious spleen and kidney necrosis virus	111362	54.78	123	AF371960	ISKNV
Megalocytivirus	*Megalocytivirus pagrus1*	Giant sea perch iridovirus - K1	112565	53.02	121	KT804738	GSIV-K1
Megalocytivirus	*Megalocytivirus pagrus1*	Large yellow croaker iridovirus	111760	53.92	131	AY779031	LYCIV
Megalocytivirus	*Megalocytivirus pagrus1*	Orange spotted grouper iridovirus	112636	53.03	125	AY894343	OSGIV
Megalocytivirus	*Megalocytivirus pagrus1*	Pompano iridovirus	112321	53.4	124	MK098185	PIV
Megalocytivirus	*Megalocytivirus pagrus1*	Red sea bream iridovirus	112414	53.41	123	BD143114	RSIV
Megalocytivirus	*Megalocytivirus pagrus1*	Rock bream iridovirus	112080	53.0	127	AY532606	RBIV
Megalocytivirus	*Megalocytivirus pagrus1*	Turbot reddish body iridovirus	110104	54.99	120	GQ273492	TRBIV
Ranavirus	*Ranavirus alytes1*	Common midwife toad virus-NL	107772	55.27	94	KP056312	CMTV-NL
Ranavirus	*Ranavirus alytes1*	Andrias davidianus ranavirus	106734	55.02	97	KC865735	ADRV
Ranavirus	*Ranavirus alytes1*	Common midwife toad virus -E	106878	55.25	90	JQ231222	CMTV-E
Ranavirus	*Ranavirus alytes1*	Pelophylax esculentus virus	107469	55.18	93	MF538627	PEV
Ranavirus	*Ranavirus alytes1*	Pike-perch iridovirus	108041	55.29	91	KX574341	PPIV
Ranavirus	*Ranavirus alytes1*	Rana esculenta virus	107444	55.2	92	MF538628	REV
Ranavirus	*Ranavirus alytes1*	Testudo hermanni ranavirus	105811	55.37	93	KP266741	THRV
Ranavirus	*Ranavirus ambystoma1*	Ambystoma tigrinum virus	106332	54.02	92	AY150217	ATV
Ranavirus	*Ranavirus epinephelus1*	Singapore grouper iridovirus	140131	48.64	141	AY521625	SGIV
Ranavirus	*Ranavirus epinephelus1*	Grouper iridovirus	139793	48.61	139	AY666015	GIV
Ranavirus	*Ranavirus gadus1*	Lumpfish ranavirus	116726	54.63	100	MH665358	LfRV
Ranavirus	*Ranavirus perca1*	Epizootic haematopoietic necrosis virus	127011	54.05	108	FJ433873	EHNV
Ranavirus	*Ranavirus perca1*	European catfish virus	127549	54.26	103	KT989885	ECV
Ranavirus	*Ranavirus perca1*	European sheatfish virus	127732	54.23	103	JQ724856	ESV
Ranavirus	*Ranavirus rana1*	Frog virus 3	105903	55.05	97	AY548484	FV3
Ranavirus	*Ranavirus rana1*	Bohle iridovirus	103531	55.16	91	KX185156	BIV
Ranavirus	*Ranavirus rana1*	Cod iridovirus	114865	54.92	100	KX574342	CoIV
Ranavirus	*Ranavirus rana1*	Frog virus 3	105070	55.01	92	KJ175144	SSME
Ranavirus	*Ranavirus rana1*	German gecko ranavirus	103681	55.06	90	KP266742	GGRV
Ranavirus	*Ranavirus rana1*	Rana grylio virus	105791	55.07	94	JQ654586	RGV
Ranavirus	*Ranavirus rana1*	Ranavirus maximus	115510	54.94	102	KX574343	Rmax

Ranavirus	*Ranavirus rana1*	Short-finned eel virus	126965	54.71	109	KX353311	SERV
Ranavirus	*Ranavirus rana1*	Soft-shelled turtle iridovirus	105890	55.06	95	EU627010	SSTV
Ranavirus	*Ranavirus rana1*	Tiger frog virus	105057	55.01	92	AF389451	TFV
Ranavirus	*Ranavirus rana1*	Tortoise ranavirus 1	103876	55.22	92	KP266743	ToRV1
Ranavirus	*Ranavirus rana1*	Zoo ranavirus	103266	55.05	91	MK227779	ZRV
Chloriridovirus	*Chloriridovirus aedes1*	Invertebrate iridescent virus 3	191100	47.89	150	DQ643392	IIV3
Chloriridovirus	*Chloriridovirus anopheles1*	Anopheles minimus iridovirus	163023	39.00	158	KF938901	AMIV
Chloriridovirus	*Chloriridovirus simulium1*	Invertebrate iridescent virus 22	197693	28.05	178	HF920633	IIV22
Chloriridovirus	*Chloriridovirus simulium1*	Invertebrate iridescent virus 30	198533	28.11	185	HF920636	IIV30[1]
Chloriridovirus	*Chloriridovirus simulium1*	Invertebrate iridescent virus 22a	196456	28.01	183	HF920634	IIV22a
Chloriridovirus	*Chloriridovirus simulium2*	Invertebrate iridescent virus 25	204815	30.32	191	HF920635	IIV25
Chloriridovirus	*Chloriridovirus wiseana1*	Invertebrate iridescent virus 9	205791	30.91	191	GQ918152	IIV9
Daphniairidovirus	*Daphniairidovirus daphnia1*	Daphnia iridescent virus 1	288858	38.75	281	LS484712	DIV1
Decapodiridovirus	*Decapodiridovirus litopenaeus1*	Shrimp hemocyte iridescent virus	165809	34.58	175	MF599468	SHIV
Decapodiridovirus	*Decapodiridovirus litopenaeus1*	Cherax quadricarnatus iridovirus	165695	34.58	172	MF197913	CQIV
Iridovirus	*Iridovirus armadillidium1*	Invertebrate iridescent virus 31	220222	35.09	221	HF920637	IIV31
Iridovirus	*Iridovirus chilo1*	Invertebrate iridescent virus 6	212482	28.63	204	AF303741	IIV6

[1]The IIV30 sequence reported by Piégu et al. [18] was labeled IIV30B in the present study to differentiate it from the IIV30C isolate from CSIRO.
