# Supplementary material for: Invertebrate Iridescent Viruses (Iridoviridae) from the Fall Armyworm, Spodoptera frugiperda
Source: Viruses. 2025 Dec 24;18(1):31. doi: 10.3390/v18010031 (PMC12846554; doi:10.3390/v18010031)
Supplement: Supplementary file 1 [file viruses-18-00031-s001.zip › Table_S10.pdf]

Table S10. IIV30C genome annotation

ORF notation	Locus tag	Product	Start	End	Strand	Identity (%)	Alignment length	E-Value	TM domain count
ORF001R*	GJGGGDMN_00097	Major capsid protein	1	1386	-	95.6	454.0	0.0	0
ORF002L	GJGGGDMN_00098	Uncharacterized 15.9 kDa protein MSP 5' region	1497	1910	+	51.9	106.0	3.99e-28	0
ORF003R	GJGGGDMN_00099	Uncharacterized protein 404L of IIV6	1942	2673	-	36.0	211.0	7.20e-29	0
ORF004R	GJGGGDMN_00100	hypothetical protein	2741	3088	-			**3.12e-18**	2
ORF005R*	GJGGGDMN_00101	Putative myristoylated protein 006R of IIV3	3106	4647	-	56.8	493.0	6.39e-194	3
ORF006L	GJGGGDMN_00102	hypothetical protein	4856	5638	+				0
ORF007R*	GJGGGDMN_00103	Uncharacterized protein 004R of IIV3	5660	6859	-	54.9	450.0	4.25e-139	0
ORF008L	GJGGGDMN_00104	hypothetical protein	6919	7326	+				0
ORF009R	GJGGGDMN_00105	Putative RING finger protein 027R of IIV3	7390	7839	-	29.9	154.0	7.76e-13	0
ORF010R	GJGGGDMN_00106	Uncharacterized protein 026R of IIV3	7943	8623	-	64.9	225.0	4.69e-101	0
ORF011R	GJGGGDMN_00107	hypothetical protein	8629	9165	-				1
ORF012L	GJGGGDMN_00108	hypothetical protein	9560	9844	+				0
ORF013L	GJGGGDMN_00109	hypothetical protein	9795	10061	+				0
ORF014R*	GJGGGDMN_00110	Ribonucleoside-diphosphate reductase small chain	9989	11092	-	77.5	351.0	3.06e-204	0
ORF015L	GJGGGDMN_00111	hypothetical protein	11199	13616	+				0
ORF016R	GJGGGDMN_00112	Uncharacterized protein 050L of IIV3	13663	14139	-	60.0	145.0	2.33e-54	0
ORF017L	GJGGGDMN_00113	Uncharacterized protein 023R of IIV3	14190	14486	+	71.3	94.0	5.55e-44	0
ORF018R	GJGGGDMN_00114	Putative Bro-N domain-containing protein 019R of IIV3	14511	15344	-	59.2	267.0	8.72e-100	0
ORF019R	GJGGGDMN_00115	Putative MSV199 domain-containing protein 420R of IIV6	15353	15712	-	46.6	103.0	2.76e-23	0
ORF020R	GJGGGDMN_00116	Uncharacterized protein 107R of IIV3	16017	16952	-	45.5	312.0	3.62e-79	0
ORF021R	GJGGGDMN_00117	Putative MSV199 domain-containing protein 468L of IIV6	17030	18322	-	39.6	356.0	3.40e-77	0
ORF022R	GJGGGDMN_00118	Uncharacterized protein 017R of IIV3	18613	19245	-	58.5	212.0	2.24e-80	0
ORF023R	GJGGGDMN_00119	Uncharacterized protein 017R of IIV3	19242	19529	-	51.7	89.0	8.49e-19	0
ORF024L	GJGGGDMN_00120	Uncharacterized protein 422L of IIV6	19654	20238	+	41.5	195.0	1.33e-35	0
ORF025R	GJGGGDMN_00121	hypothetical protein	20306	20527	-				0
ORF026R	GJGGGDMN_00122	hypothetical protein	20588	20992	-				0
ORF027R	GJGGGDMN_00123	Probable cysteine proteinase 024R of IIV3	21023	22156	-	61.4	370.0	1.24e-169	1
ORF028R	GJGGGDMN_00124	Probable cysteine proteinase 024R of IIV3	22096	22473	-	39.3	117.0	1.03e-17	0
ORF029R	GJGGGDMN_00125	Putative FAS1 domain-containing protein 081L of IIV3	22551	23117	-	33.5	188.0	3.21e-26	0
ORF030R	GJGGGDMN_00126	hypothetical protein	23131	23562	-				0
ORF031L	GJGGGDMN_00127	Uncharacterized protein 115R of IIV3	23697	23954	+	60.3	78.0	5.66e-29	0
ORF032L	GJGGGDMN_00128	hypothetical protein	24039	24431	+				0
ORF033L	GJGGGDMN_00129	Uncharacterized protein 119R of IIV3	24530	25054	+	70.6	51.0	6.19e-20	0
ORF034R	GJGGGDMN_00130	hypothetical protein	25110	25787	-				0
ORF035R	GJGGGDMN_00131	Dual specificity phosphatase	25821	26321	-	45.9	133.0	3.78e-31	0
ORF036L	GJGGGDMN_00132	High mobility group protein homolog 068R of IIV3	26472	27062	+	83.7	202.0	1.46e-116	0

ORF037L	GJGGGDMN_00133	hypothetical protein	27208	27465	+				0
ORF038R	GJGGGDMN_00134	RNA polymerase Rpb1, domain 3	27643	31656	-			**0.0**	0
ORF039R*	GJGGGDMN_00135	XPG I-region	32148	32624	-	70.3	1375.0	0.0	0
ORF040R	GJGGGDMN_00136	XPG I-region	32647	33246	-	50.0	100.0	1.35e-27	0
ORF041R*	GJGGGDMN_00137	hypothetical protein	33560	34087	-	54.7	201.0	5.09e-67	0
ORF042R	GJGGGDMN_00138	hypothetical protein	34204	34422	-				0
ORF043R	GJGGGDMN_00139	hypothetical protein	34455	34682	-			**1.13e-28**	0
ORF044R	GJGGGDMN_00140	hypothetical protein	34646	35044	-				1
ORF045R	GJGGGDMN_00141	Putative MSV199 domain-containing protein 238R of IIV6	35107	36294	-	36.9	409.0	4.79e-74	0
ORF046R	GJGGGDMN_00142	Putative MSV199 domain-containing protein 093L of IIV3	36341	36595	-	48.8	80.0	4.70e-17	0
ORF047L	GJGGGDMN_00143	Uncharacterized protein 113L of IIV3	36712	37932	+	58.6	391.0	3.89e-141	0
ORF048L	GJGGGDMN_00144	Uncharacterized protein 113L of IIV3	37878	39041	+	46.2	405.0	2.83e-113	0
ORF049L	GJGGGDMN_00145	Trypsin Inhibitor like cysteine rich domain	39150	39377	+	49.1	57.0	1.01e-16	0
ORF050R	GJGGGDMN_00146	Uncharacterized protein 112R of IIV3	39420	39773	-	47.8	113.0	3.06e-27	1
ORF051R	GJGGGDMN_00147	phosphatase activity	39823	40275	-	63.2	152.0	2.47e-68	0
ORF052L	GJGGGDMN_00148	Uncharacterized protein 001R of IIV3	40371	40580	+	53.8	65.0	1.20e-17	0
ORF053L	GJGGGDMN_00149	Uncharacterized protein 001R of IIV3	40678	40935	+	44.8	67.0	2.48e-13	0
ORF054R	GJGGGDMN_00150	Uncharacterized protein 092R of IIV3	41107	41622	-	64.2	173.0	1.09e-70	0
ORF055R	GJGGGDMN_00151	kinase activity	41637	42146	-			**3.58e-68**	0
ORF056R	GJGGGDMN_00152	hypothetical protein	42253	43473	-				0
ORF057L	GJGGGDMN_00153	Uncharacterized protein 032R of IIV3	43708	44571	+	39.8	133.0	1.30e-17	0
ORF058L	GJGGGDMN_00154	hypothetical protein	44722	45393	+				0
ORF059L	GJGGGDMN_00155	hypothetical protein	45826	46233	+				0
ORF060L	GJGGGDMN_00156	hypothetical protein	46316	46576	+				0
ORF061L	GJGGGDMN_00157	Putative MSV199 domain-containing protein 420R of IIV6	46585	46713	+	35.7	412.0	2.35e-62	0
ORF062R	GJGGGDMN_00158	DNA binding	46735	48009	-	63.6	286.0	4.20e-123	0
ORF063R*	GJGGGDMN_00159	Uncharacterized protein 016R of IIV3	48082	49413	-	50.1	1150.0	0.0	0
ORF064L	GJGGGDMN_00160	Uncharacterized protein 072L of IIV3	50577	53930	+	59.6	156.0	1.46e-58	0
ORF065R	GJGGGDMN_00161	Uncharacterized protein 073R of IIV3	53960	54424	-	62.3	69.0	6.74e-25	0
ORF066L	GJGGGDMN_00162	hypothetical protein	54515	54721	+	57.6	337.0	1.49e-137	0
ORF067L	GJGGGDMN_00163	hypothetical protein	54905	55075	+	55.9	238.0	0	0
ORF068R*	GJGGGDMN_00164	bis(5'-nucleosyl)-tetraphosphatase (symmetrical) activity	55195	56736	-			**1.50e-139**	0
ORF069L	GJGGGDMN_00165	Matrix metalloproteinase-2	56784	57503	+	53.8	132.0	1.12e-38	0
ORF070L	GJGGGDMN_00166	hypothetical protein	57558	58664	+				0
ORF071R*	GJGGGDMN_00167	extracellular matrix	58693	59349	-	47.2	108.0	5.22e-38	1
ORF072R	GJGGGDMN_00168	Uncharacterized protein 082L of IIV3	59451	59795	-	28.9	152.0	3.06e-13	0
ORF073L	GJGGGDMN_00169	Erv1/Alr family	59875	60342	+			5.24e-37	1
ORF074R	GJGGGDMN_00170	Putative MSV199 domain-containing protein 420R of IIV6	60491	60931	-	32.3	415.0	6.30e-53	0
ORF075R	GJGGGDMN_00171	hypothetical protein	60996	61106	-				0
ORF076R	GJGGGDMN_00172	DNA binding	61137	62429	-	60.9	939.0	2.36e-273	0

ORF077R	GJGGGDMN_00173	hypothetical protein	62461	62955	-			**1.09e-15**	0
ORF078R	GJGGGDMN_00174	OTU-like cysteine protease	63004	65667	-			**1.52e-44**	0
ORF079R	GJGGGDMN_00175	Uncharacterized protein 123L of IIV3	65812	66045	-	38.6	132.0	2.10e-21	0
ORF080L	GJGGGDMN_00176	Uncharacterized protein 124R of IIV3	66068	66214	+	35.8	229.0	7.21e-24	0
ORF081R	GJGGGDMN_00177	Uncharacterized protein 443R of IIV6	66253	66651	-	40.1	818.0	9.66e-105	0
ORF082L	GJGGGDMN_00178	Uncharacterized protein 125R of IIV3	66670	67296	+	49.4	257.0	5.44e-91	0
ORF083L	GJGGGDMN_00179	Uncharacterized protein 126R of IIV3	67372	71346	+	46.7	105.0	1.96e-23	2
ORF084L*	GJGGGDMN_00180	hypothetical protein	71363	72226	+	69.3	942.0	0.0	0
ORF085L	GJGGGDMN_00181	Uncharacterized protein 019R of IIV3	72260	72547	+	40.7	285.0	2.35e-61	0
ORF086L	GJGGGDMN_00182	Transmembrane protein 049L of IIV6	72920	73078	+	63.5	74.0	1.14e-22	3
ORF087R*	GJGGGDMN_00183	D5 N terminal like	73091	75910	-	47.9	1590.0	0.0	0
ORF088L	GJGGGDMN_00184	DNA binding	76225	77088	+			**3.53e-10**	0
ORF089R	GJGGGDMN_00185	Putative MSV199 domain-containing protein 238R of IIV6	77132	77437	-	40.4	423.0	4.90e-90	0
ORF090R	GJGGGDMN_00186	Uncharacterized protein 051L of IIV3	77510	82282	-	32.5	320.0	3.23e-41	0
ORF091R	GJGGGDMN_00187	Uncharacterized protein 404L of IIV6	82375	82650	-	64.7	215.0	1.71e-99	0
ORF092R*	GJGGGDMN_00188	acid-amino acid ligase activity	82864	84210	-	80.3	768.0	0.0	0
ORF093L	GJGGGDMN_00189	hypothetical protein	84326	85792	+	71.0	362.0	1.37e-166	0
ORF094R	GJGGGDMN_00190	Uncharacterized protein 443R of IIV6	85897	86580	-	39.3	349.0	2.55e-37	0
ORF095R	GJGGGDMN_00191	DNA-directed RNA polymerase subunit 2	86640	88946	-			**0.0**	0
ORF096R	GJGGGDMN_00192	Putative KilA-N domain-containing protein 006L of IIV6	88952	90010	-	45.5	165.0	4.04e-38	0
ORF097L	GJGGGDMN_00193	Putative MSV199 domain-containing protein 211L of IIV6	90074	92050	+	48.4	188.0	5.91e-54	0
ORF098L	GJGGGDMN_00194	Uncharacterized protein 007R of IIV3	92063	92293	+	50.1	409.0	3.32e-126	0
ORF099R	GJGGGDMN_00195	Putative MSV199 domain-containing protein 238R of IIV6	92329	92838	-	36.6	443.0	1.64e-77	0
ORF100R	GJGGGDMN_00196	acid-amino acid ligase activity	92849	93436	-	42.1	553.0	4.45e-123	0
ORF101R	GJGGGDMN_00197	hypothetical protein	93525	94802	-				0
ORF102R	GJGGGDMN_00198	Uncharacterized protein 053L of IIV3	94888	96375	-	48.2	141.0	1.05e-39	0
ORF103R	GJGGGDMN_00199	DNA ligase	96485	98317	-			**1.96e-170**	0
ORF104R	GJGGGDMN_00200	Uncharacterized protein 094L of IIV3	98822	99241	-	33.3	730.0	2.44e-96	0
ORF105R	GJGGGDMN_00201	Uncharacterized protein 094L of IIV3	99275	99652	-	64.4	104.0	4.99e-39	0
ORF106R	GJGGGDMN_00202	hypothetical protein	99751	101841	-				0
ORF107R*	GJGGGDMN_00203	Uncharacterized protein 033L of IIV3	101858	102181	-	62.9	178.0	3.80e-85	0
ORF108R	GJGGGDMN_00204	Putative MSV199 domain-containing protein 468L of IIV6	102289	103101	-	41.6	361.0	2.19e-78	0
ORF109L	GJGGGDMN_00205	hypothetical protein	103195	103758	+			**9.47e-97**	0
ORF110R	GJGGGDMN_00206	Uncharacterized protein 032R of IIV3	103789	104883	-	50.7	138.0	9.05e-37	0
ORF111R	GJGGGDMN_00207	protein phosphatase 1, regulatory subunit 15B	104930	105289	-			**5.49e-11**	0
ORF112R	GJGGGDMN_00208	hypothetical protein	105373	106092	-			**4.97e-52**	0
ORF113R	GJGGGDMN_00209	hypothetical protein	106166	106585	-				0
ORF114L	GJGGGDMN_00001	Uncharacterized protein 261R of IIV6	108034	108444	+	52.5	61.0	3.16e-10	0
ORF115L	GJGGGDMN_00002	Uncharacterized protein 219L of IIV6	108420	109262	+	44.2	208.0	1.02e-41	0

ORF116L	GJGGGDMN_00003	uncharacterized protein 030L of IIV3	109275	109544	+	52.6	78.0	5.75e-21	0
ORF117R*	GJGGGDMN_00004	Putative kinase protein 029R of IIV3	109700	110272	-	57.9	190.0	7.12e-79	0
ORF118R	GJGGGDMN_00005	Uncharacterized protein 028R of IIV3	110316	111206	-	34.4	294.0	4.44e-46	0
ORF119R	GJGGGDMN_00006	Thymidylate synthase	111572	112465	-	50.2	297.0	6.10e-106	0
ORF120L	GJGGGDMN_00007	Uncharacterized protein 061R of IIV6	112529	113935	+	34.3	478.0	2.08e-80	0
ORF121L	GJGGGDMN_00008	hypothetical protein	114014	114229	+				0
ORF122R	GJGGGDMN_00009	hypothetical protein	114674	115195	-				0
ORF123L	GJGGGDMN_00010	Uncharacterized protein 063R of IIV6	115280	115954	+	42.3	227.0	2.84e-46	0
ORF124R	GJGGGDMN_00011	hypothetical protein	115994	116635	-				0
ORF125R	GJGGGDMN_00012	DNA topoisomerase 2	116655	118919	-	63.6	748.0	0.0	0
ORF126R	GJGGGDMN_00013	DNA topoisomerase large subunit	118879	120042	-	53.7	389.0	3.56e-128	0
ORF127L	GJGGGDMN_00014	hypothetical protein	120274	120453	+				1
ORF128L	GJGGGDMN_00015	Uncharacterized protein 099R of IIV3	120541	121530	+	59.4	202.0	7.01e-72	0
ORF129R	GJGGGDMN_00016	Uncharacterized protein 378R of IIV6	121561	122244	-	64.5	217.0	3.93e-71	0
ORF130R	GJGGGDMN_00017	Uncharacterized protein 443R of IIV6	122295	124550	-	63.0	119.0	2.52e-34	0
ORF131R	GJGGGDMN_00018	Uncharacterized protein 443R of IIV6	124580	128914	-	34.2	1337.0	4.47e-103	0
ORF132L	GJGGGDMN_00019	Uncharacterized protein 074L of IIV3	129006	131102	+	45.0	805.0	6.18e-214	0
ORF133L	GJGGGDMN_00020	Ribonucleotide reductase, barrel domain	131293	133560	+	57.0	761.0	1.41e-291	0
ORF134L	GJGGGDMN_00021	Putative MSV199 domain-containing protein 211L of IIV6	133657	134433	+	50.9	167.0	5.37e-44	0
ORF135L	GJGGGDMN_00022	Putative KilA-N domain-containing protein 006L of IIV6	134405	134926	+	45.9	157.0	5.06e-32	0
ORF136L	GJGGGDMN_00023	hypothetical protein	134941	135117	+				0
ORF137R	GJGGGDMN_00024	Uncharacterized protein 219L of IIV6	135169	135984	-	30.0	230.0	4.88e-09	0
ORF138R	GJGGGDMN_00025	Uncharacterized protein 042R of IIV3	136113	136589	-	58.5	159.0	1.47e-62	0
ORF139R	GJGGGDMN_00026	Poxvirus Late Transcription Factor VLTF3 like	136821	137885	-	67.6	327.0	1.62e-143	0
ORF140L	GJGGGDMN_00027	mRNA-decapping protein D10	138054	138728	+	40.4	218.0	2.60e-45	0
ORF141L	GJGGGDMN_00028	Uncharacterized protein L5	138977	140368	+	29.2	431.0	8.67e-38	0
ORF142L*	GJGGGDMN_00029	Uncharacterized protein 088R of IIV3	140365	141123	+	76.5	251.0	8.12e-144	0
ORF143R	GJGGGDMN_00030	hypothetical protein	141169	141705	-				0
ORF144L	GJGGGDMN_00031	UPF0213 protein CKO_04549	141826	142209	+	55.6	72.0	1.50e-17	0
ORF145R	GJGGGDMN_00032	Putative serine/threonine-protein kinase 040L of IIV3	142234	143193	-	49.4	330.0	1.42e-105	0
ORF146L	GJGGGDMN_00033	Uncharacterized protein 045R of IIV3	143306	143629	+	68.1	94.0	4.11e-36	0
ORF147R	GJGGGDMN_00034	Putative MSV199 domain-containing protein 238R of IIV6	143733	144386	-	41.8	249.0	0.0	0
ORF148R	GJGGGDMN_00035	Uncharacterized protein 019R of IIV6	144376	145149	-	41.7	230.0	4.03e-51	0
ORF149L	GJGGGDMN_00036	Uncharacterized protein 229L of IIV6	145302	146579	+	49.0	418.0	5.31e-132	0
ORF150L	GJGGGDMN_00037	Uncharacterized protein 443R of IIV6	146623	149310	+	43.8	640.0	2.24e-99	0
ORF151L	GJGGGDMN_00038	Uncharacterized protein 043R of IIV3	149323	149514	+	74.6	63.0	2.09e-32	2
ORF152R*	GJGGGDMN_00039	Uncharacterized protein 038R of IIV3	149725	151377	-	53.7	547.0	1.30e-203	0
ORF153L	GJGGGDMN_00040	hypothetical protein	151441	152322	+				1
ORF154R	GJGGGDMN_00041	Putative thioredoxin-like protein 041R of IIV3	152363	152719	-	55.1	118.0	1.31e-46	0

ORF155R	GJGGGDMN_00042	hypothetical protein	152746	152907	-				0
ORF156R	GJGGGDMN_00043	Double-stranded RNA binding motif	152953	153396	-			**1.20e-48**	0
ORF157R	GJGGGDMN_00044	Immediate-early protein ICP-46 homolog	153494	154828	-	54.4	441.0	5.62e-157	0
ORF158L*	GJGGGDMN_00045	protein serine/threonine kinase activity	154914	156449	+	58.8	512.0	2.47e-209	0
ORF159R	GJGGGDMN_00046	Putative SWIB domain-containing protein 070L of IIV3	156489	157229	-	56.8	229.0	2.19e-73	0
ORF160R	GJGGGDMN_00047	Putative MSV199 domain-containing protein 420R of IIV6	157565	157690	-	62.1	29.0	2.96e-07	0
ORF161L	GJGGGDMN_00048	N-methyltransferase activity	157705	160614	+	62.5	984.0	0.0	0
ORF162R	GJGGGDMN_00049	Putative KilA-N domain-containing protein 006L of IIV6	160643	161065	-	49.6	127.0	9.08e-31	0
ORF163R	GJGGGDMN_00050	Putative MSV199 domain-containing protein 468L of IIV6	161062	161763	-	41.1	224.0	1.78e-52	0
ORF164R	GJGGGDMN_00051	Uncharacterized protein 060L of IIV3	161968	162717	-	53.0	264.0	1.31e-82	0
ORF165L	GJGGGDMN_00052	hypothetical protein	162895	163293	+				0
ORF166L	GJGGGDMN_00053	Uncharacterized protein 058R of IIV3	163358	163789	+	67.4	138.0	3.35e-67	0
ORF167R	GJGGGDMN_00054	hypothetical protein	164021	164518	-				0
ORF168R	GJGGGDMN_00055	XRN 5'-3' exonuclease N-terminus	164628	166319	-			**1.48e-259**	0
ORF169L	GJGGGDMN_00056	hypothetical protein	166492	166755	+	63.6	574.0	2.98e-260	0
ORF170R	GJGGGDMN_00057	Dihydrofolate reductase	166744	167283	-			**2.32e-42**	3
ORF171R	GJGGGDMN_00058	acid-amino acid ligase activity	167325	168464	-	37.6	178.0	1.20e-40	0
ORF172R	GJGGGDMN_00059	Putative MSV199 domain-containing protein 468L of IIV6	168540	169109	-	44.4	356.0	5.67e-83	0
ORF173L	GJGGGDMN_00060	hypothetical protein	169240	169848	+	47.5	141.0	1.06e-34	1
ORF174R	GJGGGDMN_00061	Uncharacterized protein 97L	169888	170385	-	55.2	203.0	2.00e-72	0
ORF175L	GJGGGDMN_00062	Uncharacterized protein 020R of IIV3	170878	171513	+	57.7	163.0	1.16e-62	0
ORF176R	GJGGGDMN_00063	Uncharacterized protein 071L	171550	172986	-	69.5	197.0	5.04e-86	0
ORF177L*	GJGGGDMN_00064	Uncharacterized protein 106R of IIV3	173118	173984	+	61.4	469.0	2.09e-207	0
ORF178L	GJGGGDMN_00065	hypothetical protein	174158	174403	+				0
ORF179L	GJGGGDMN_00066	Uncharacterized protein 159L of IIV6	174524	174874	+	35.5	93.0	1.02e-07	0
ORF180R	GJGGGDMN_00067	Uncharacterized protein 105R of IIV3	174920	175651	-	64.4	247.0	1.35e-109	0
ORF181L*	GJGGGDMN_00068	Putative CTD phosphatase-like protein 355R of IIV3	175772	176332	+	69.2	185.0	8.33e-94	0
ORF182R	GJGGGDMN_00069	Zinc finger, C3HC4 type (RING finger)	176383	177048	-	51.3	228.0	1.04e-62	0
ORF183R	GJGGGDMN_00070	Transmembrane protein 022L of IIV3	177113	177742	-	50.6	172.0	8.99e-55	6
ORF184R*	GJGGGDMN_00071	ribonuclease III activity	177956	178225	-	76.4	140.0	7.28e-69	0
ORF185R	GJGGGDMN_00072	ribonuclease III activity	178368	178829	-			**3.93e-69**	0
ORF186R	GJGGGDMN_00073	Putative MSV199 domain-containing protein 420R of IIV6	178896	179582	-	31.6	414.0	6.90e-57	0
ORF187L*	GJGGGDMN_00074	Putative membrane protein 047R of IIV3	179747	180994	+	85.7	126.0	1.03e-77	2
ORF188R	GJGGGDMN_00075	Putative membrane protein 047R of IIV3	181244	182119	-	64.5	62.0	8.70e-24	0
ORF189R	GJGGGDMN_00076	Uncharacterized protein 102R of IIV3	182311	182562	-	58.3	115.0	1.45e-34	0
ORF190R	GJGGGDMN_00077	Uncharacterized protein 054L of IIV3	182609	182962	-	46.2	253.0	6.21e-72	0
ORF191R	GJGGGDMN_00078	Uncharacterized protein 085L of IIV3	183080	183886	-	65.0	137.0	3.59e-63	1
ORF192L	GJGGGDMN_00079	Hypothetical protein	184054	184524	+			**1.02e-61**	0
ORF193L	GJGGGDMN_00080	Putative zinc finger protein 012R of IIV3	184624	184953	+	43.2	387.0	5.79e-101	0
ORF194R*	GJGGGDMN_00081	Probable serine/threonine-protein kinase 380R of IIV6	184979	186139	-	41.1	545.0	1.12e-114	0

ORF195L*	GJGGGDMN_00082	Uncharacterized protein 056L of IIV3	186326	187894	+	43.8	345.0	3.71e-88	0
ORF196R	GJGGGDMN_00083	hypothetical protein	187953	188978	-			**5.82e-143**	0
ORF197R	GJGGGDMN_00084	hypothetical protein	189117	189554	-				0
ORF198R	GJGGGDMN_00085	Uncharacterized protein 069L of IIV3	189608	190498	-	45.9	425.0	6.52e-117	0
ORF199L	GJGGGDMN_00086	Putative Bro-N domain-containing protein 019R of IIV3	190545	191816	+	50.8	327.0	9.69e-91	0
ORF200R	GJGGGDMN_00087	Putative MSV199 domain-containing protein 200R of IIV6	191979	193202	-	54.8	31.0	9.03e-09	0
ORF201R*	GJGGGDMN_00088	Putative transcription elongation factor S-II-like protein 055R of IIV3	193578	193985	-	49.2	130.0	2.47e-42	0
ORF202R	GJGGGDMN_00089	hypothetical protein	194048	194263	-				0
ORF203R*	GJGGGDMN_00090	Uncharacterized protein 035R of IIV3	194314	197256	-	53.4	979.0	0.0	0
ORF204R	GJGGGDMN_00091	Uncharacterized protein IIV3-013L	197378	197641	-	50.5	91.0	1.49e-20	1
ORF205R	GJGGGDMN_00092	hypothetical protein	197712	198026	-				0
ORF206R	GJGGGDMN_00093	hypothetical protein	198023	198253	-				0
ORF207R	GJGGGDMN_00094	Uncharacterized protein 009R of IIV6	198298	198555	-	57.5	80.0	7.27e-29	0
ORF208L	GJGGGDMN_00095	hypothetical protein	198695	199294	+			**1.66e-48**	0
ORF209L	GJGGGDMN_00096	Uncharacterized protein 273R of IIV6	199242	200108	+	44.0	134.0	4.62e-28	0

E-values: Gene annotation by Blast (normal text) or eggNOG-mapper (bold text).
*ORFs labeled with an asterisk denote core genes recovered and validated with VIGA across all five sequenced IIV genomes from lepidopteran hosts.
TM domain content – transmembrane domains (alpha-helices) detected by using DeepTMHMM.
