# Supplementary material for: Invertebrate Iridescent Viruses (Iridoviridae) from the Fall Armyworm, Spodoptera frugiperda
Source: Viruses. 2025 Dec 24;18(1):31. doi: 10.3390/v18010031 (PMC12846554; doi:10.3390/v18010031)
Supplement: Supplementary file 1 [file viruses-18-00031-s001.zip › Table_S2.pdf]

Table S2. Slope and odds ratio for IIV infection in relation to SfMNPV infection, nematode infection and parasitism by parasitoids in field experiments performed in Chiapas State, Mexico.

Association	β (slope)	SE	Odds ratio	95% CI lower	95% CI upper
Experiment 1					
IIV - SfMNPV	-0.007	0.235	0.993	0.627	1.573
IIV - parasitoids	0.115	0.225	1.122	0.722	1.744
IIV - nematodes	-0.045	0.165	0.956	0.692	1.321
Experiment 2					
IIV - SfMNPV	0.370	0.185	1.448	1.007	2.082
IIV - parasitoids	-0.652	0.301	0.521	0.289	0.940
IIV - nematodes	-0.063	0.222	0.939	0.608	1.450

95% CI: upper and lower limits of 95% confidence interval.
