# Supplementary material for: Invertebrate Iridescent Viruses (Iridoviridae) from the Fall Armyworm, Spodoptera frugiperda
Source: Viruses. 2025 Dec 24;18(1):31. doi: 10.3390/v18010031 (PMC12846554; doi:10.3390/v18010031)
Supplement: Supplementary file 1 [file viruses-18-00031-s001.zip › Table_S3.pdf]

Table S3. Sequencing statistics and assembly metrics of IIV genomes

Isolate	Yield[1]	Percentage mapped	Mean coverage	Read N50 (bp)	Assembler	Contigs[2]	Assembly N50 (bp)	Total length (bp)
SfIIV-Chi	444	97.30	11.07×	12986	Flye	1	197661	197661
SfIIV-Ver	3215	96.77	18.50×	2685	Flye	1	201630	201630
SfIIV-Arg	3428	98.05	23.03×	3091	Flye	1	196053	196053
IIV30C	187	51.34	7.55×	13916	Flye	1	200215	200215
AgIIV	1520	98.22	32.02×	5939	Flye	1	205445	205445

[1] Yield indicates the number of ONT reads retained in the final BAM files. Mapping statistics were obtained using Samtools flagstat, and mean/median coverage was calculated from per-base depth (Samtools depth). Read-length N50 was computed from aligned ONT reads. Final genome assemblies correspond to single-contig Flye assemblies. For the SfIIV-Chi isolate, hybrid assembly did not increase contiguity relative to ONT-only assemblies, which produced a single complete contig spanning the entire genome. Short reads were therefore used only for base-level validation and did not alter the genome structure, length or organization.

[2] All Flye assemblies were obtained as single linear contigs, consistent with the known linear dsDNA architecture of the *Iridoviridae*. No artificial genome closure was introduced into circular maps. Tandem repeats did not generate assembly breaks or collapsed regions. MUMmer self-alignments confirmed structurally consistent assemblies, and Mauve maps reproduced the expected genome organization. Inversions identified relative to reference genomes were validated by long-read evidence, with Nanopore reads spanning both breakpoints without clipping or read splitting.
