# Supplementary material for: Invertebrate Iridescent Viruses (Iridoviridae) from the Fall Armyworm, Spodoptera frugiperda
Source: Viruses. 2025 Dec 24;18(1):31. doi: 10.3390/v18010031 (PMC12846554; doi:10.3390/v18010031)
Supplement: Supplementary file 1 [file viruses-18-00031-s001.zip › Table_S4.pdf]

Table S4. ORF functional categories summary for IIVs from lepidopteran hosts

Virus	COG S (%)	COG L (%)	COG K (%)	COG V (%)	COG H (%)
SfIIV-Chi	34.1	2.2	0.5	0.5	0.5
SfIIV-Ver	33.7	1.6	0.5	0.5	0.5
SfIIV-Arg	33.7	2.0	0.5	0.5	0.5
AgIIV	34.4	2.6	1.0	0.5	0.5
IIV30C	33.5	2.9	0.5	0.5	0.5
