# Supplementary material for: Invertebrate Iridescent Viruses (Iridoviridae) from the Fall Armyworm, Spodoptera frugiperda
Source: Viruses. 2025 Dec 24;18(1):31. doi: 10.3390/v18010031 (PMC12846554; doi:10.3390/v18010031)
Supplement: Supplementary file 1 [file viruses-18-00031-s001.zip › Table_S5.pdf]

Table S5. Hypothetical proteins and transmembrane domains (TM) present in IIV genomes

Genome	Total CDS	CDS with TM	CDS with TM (%)	Hypothetical proteins	Hypotheticals with TM	Hypotheticals with TM (%)
SfIIV-Chi	185	19	10.3%	43	6	14.0%
SfIIV-Ver	184	18	9.8%	44	5	11.4%
SfIIV-Arg	196	20	10.2%	50	7	14.0%
AgIIV	195	19	9.7%	47	7	14.9%
IIV30C	209	19	9.1%	53	6	11.3%

CDS, coding DNA sequence

Hypothetical proteins were detected using the virus genome assembly tool VIGA; TM domains were detected using DeepTMHMM.
