# Supplementary material for: Invertebrate Iridescent Viruses (Iridoviridae) from the Fall Armyworm, Spodoptera frugiperda
Source: Viruses. 2025 Dec 24;18(1):31. doi: 10.3390/v18010031 (PMC12846554; doi:10.3390/v18010031)
Supplement: Supplementary file 1 [file viruses-18-00031-s001.zip › Table_S6.pdf]

Table S6. SfIIV-Chi genome annotation

ORF	Locus tag	Product	Start	End	Strand	Identity (%)	Alignment length (bp)	E-Value	TM domain count
ORF001L*	EAMDCAEI_00003	Major capsid protein	1	1386	+	96.0	454	0.0	0
ORF002R	EAMDCAEI_00004	Uncharacterized protein 273R of IIV6	1485	2765	-	44.4	142	4.97e-27	0
ORF003L	EAMDCAEI_00005	Uncharacterized protein 009R of IIV6	2830	3090	+	59.3	81	7.08e-34	0
ORF004L	EAMDCAEI_00006	hypothetical protein	3138	3362	+				0
ORF005L	EAMDCAEI_00007	hypothetical protein	3359	3676	+				0
ORF006L	EAMDCAEI_00008	Uncharacterized protein IIV3-013L	3728	3991	+	47.3	91	6.98e-19	1
ORF007L*	EAMDCAEI_00009	Uncharacterized protein 035R of IIV3	4095	7301	+	51.1	1103	0.0	0
ORF008L*	EAMDCAEI_00010	Putative transcription elongation factor S-II-like protein 055R of IIV3	7349	7771	+	46.8	139	1.74e-42	0
ORF009L	EAMDCAEI_00011	Putative Bro-N domain-containing protein 019R of IIV3	8032	9423	+	55.1	285	1.13e-89	0
ORF010R	EAMDCAEI_00012	Uncharacterized protein 069L of IIV3	9586	10857	-	46.1	425	4.05e-118	0
ORF011L	EAMDCAEI_00013	hypothetical protein	10904	11794	+			**2.00e-113**	0
ORF012L	EAMDCAEI_00014	hypothetical protein	11847	12284	+				0
ORF013L*	EAMDCAEI_00015	Uncharacterized protein 056L of IIV3	12432	13451	+	43.8	345	3.46e-88	0
ORF014R*	EAMDCAEI_00016	Probable serine/threonine-protein kinase 380R of IIV6	13499	15082	-	42.0	550	9.34e-115	0
ORF015L	EAMDCAEI_00017	Putative zinc finger protein 012R of IIV3	15248	16393	+	43.2	382	1.72e-101	0
ORF016R	EAMDCAEI_00018	Uncharacterized protein 085L of IIV3	16844	17314	-	65.0	137	2.53e-63	1
ORF017L	EAMDCAEI_00019	Uncharacterized protein 054L of IIV3	17483	18313	+	48.2	247	1.12e-74	0
ORF018L	EAMDCAEI_00020	Uncharacterized protein 102R of IIV3	18411	18764	+	60.0	115	6.21e-36	0
ORF019L	EAMDCAEI_00021	Putative membrane protein 047R of IIV3	18825	19076	+	64.5	62	8.70e-24	0
ORF020L*	EAMDCAEI_00022	Putative membrane protein 047R of IIV3	19268	20107	+	85.7	126	9.51e-78	2
ORF021R	EAMDCAEI_00023	hypothetical protein	20478	20606	-				0
ORF022L	EAMDCAEI_00024	hypothetical protein	20762	21580	+				0
ORF023L*	EAMDCAEI_00025	ribonuclease III activity	21674	22510	+	76.4	280	2.67e-148	0
ORF024L	EAMDCAEI_00026	Transmembrane protein 022L of IIV3	22730	23359	+	51.7	172	4.48e-55	6
ORF025L	EAMDCAEI_00027	Zinc finger, C3HC4 type (RING finger)	23422	24087	+	51.3	228	4.22e-62	0
ORF026R*	EAMDCAEI_00028	Putative CTD phosphatase-like protein 355R of IIV3	24114	24674	-	63.8	185	5.09e-81	0
ORF027L	EAMDCAEI_00029	Uncharacterized protein 105R of IIV3	24797	25528	+	65.0	246	2.46e-112	0
ORF028R	EAMDCAEI_00030	Uncharacterized protein 159L of IIV6	25573	27054	-	32.5	231	2.48e-25	0
ORF029R	EAMDCAEI_00031	Uncharacterized protein 159L of IIV6	27139	28488	-	33.0	233	1.78e-28	0
ORF030R	EAMDCAEI_00032	hypothetical protein	28503	28610	-				0
ORF031L*	EAMDCAEI_00033	Uncharacterized protein 106R of IIV3	28742	30175	+	61.9	465	8.17e-207	0
ORF032R	EAMDCAEI_00034	Uncharacterized protein 071L of IIV3	30283	30927	-	69.6	194	2.29e-85	0
ORF033L	EAMDCAEI_00035	Uncharacterized protein 020R of IIV3	32237	32734	+	57.7	163	2.34e-62	0
ORF034R	EAMDCAEI_00036	Putative MSV199 domain-containing protein 420R of IIV6	32804	34120	-	39.3	448	2.01e-94	0
ORF035R	EAMDCAEI_00037	Uncharacterized protein 97L of IIV3	34147	34596	-	57.6	139	8.12e-53	0
ORF036R	EAMDCAEI_00038	Uncharacterized protein 97L of IIV3	34605	34748	-	62.2	45	2.33e-10	0
ORF037L	EAMDCAEI_00039	dUTPase	34879	35448	+	46.8	141	9.43e-36	1

ORF038L	EAMDCAEI_00040	Putative MSV199 domain-containing protein 468L of IIV6	35547	36842	+	42.3	355	4.73e-90	0
ORF039L	EAMDCAEI_00041	Dihydrofolate reductase	36875	37417	+	37.6	178	5.67e-39	0
ORF040R	EAMDCAEI_00042	hypothetical protein	37406	37669	-				3
ORF041L	EAMDCAEI_00043	XRN 5'-3' exonuclease N-terminus	37844	39538	+	63.4	571	1.33e-261	0
ORF042L	EAMDCAEI_00044	hypothetical protein	39653	40150	+				0
ORF043L	EAMDCAEI_00045	hypothetical protein	40256	40396	+				0
ORF044R	EAMDCAEI_00046	Uncharacterized protein 058R of IIV3	40437	40868	-	67.4	138	6.76e-67	0
ORF045R	EAMDCAEI_00047	hypothetical protein	40929	41327	-				0
ORF046L	EAMDCAEI_00048	Uncharacterized protein 060L of IIV3	41494	42192	+	51.4	247	7.38e-71	0
ORF047L	EAMDCAEI_00049	Putative MSV199 domain-containing protein 238R of IIV6	42588	44045	+	42.3	442	2.31e-98	0
ORF048R	EAMDCAEI_00050	N-methyltransferase activity	44085	46994	-	62.6	984	0.0	0
ORF049L	EAMDCAEI_00051	Putative Bro-N domain-containing protein 019R of IIV3	47215	48570	+	49.7	342	1.77e-94	0
ORF050L	EAMDCAEI_00052	Putative SWIB domain-containing protein 070L of IIV3	48705	49442	+	55.9	229	6.01e-73	0
ORF051R*	EAMDCAEI_00053	protein serine/threonine kinase activity	49481	51016	-	59.0	512	4.97e-209	0
ORF052L	EAMDCAEI_00054	Immediate-early protein ICP-46 homolog	51118	51585	+	45.1	153	1.28e-36	0
ORF053L	EAMDCAEI_00055	Immediate-early protein ICP-46 homolog	51654	51929	+	53.9	89	1.19e-25	0
ORF054L*	EAMDCAEI_00056	Immediate-early protein ICP-46 homolog	51895	52452	+	52.8	178	6.70e-50	0
ORF055L	EAMDCAEI_00057	Double-stranded RNA binding motif	52598	52987	+			**7.27e-43**	0
ORF056L	EAMDCAEI_00058	hypothetical protein	53049	53201	+				0
ORF057L	EAMDCAEI_00059	Putative thioredoxin-like protein 041R of IIV3	53229	53585	+	55.9	118	4.59e-47	0
ORF058R	EAMDCAEI_00060	hypothetical protein	53623	54249	-				1
ORF059L*	EAMDCAEI_00061	Uncharacterized protein 038R of IIV3	54314	55966	+	54.3	549	4.89e-206	0
ORF060R	EAMDCAEI_00062	Uncharacterized protein 043R of IIV3	56175	56366	-	74.6	63	2.09e-32	2
ORF061R	EAMDCAEI_00063	Uncharacterized protein 443R of IIV6	56381	63232	-	31.5	1848	1.96e-118	0
ORF062L	EAMDCAEI_00064	Uncharacterized protein 074L of IIV3	63335	65431	+	45.4	808	1.36e-215	0
ORF063L	EAMDCAEI_00065	Ribonucleotide reductase	65549	67888	+	57.1	785	5.36e-300	0
ORF064L	EAMDCAEI_00066	hypothetical protein	68002	68487	+			**5.96e-10**	0
ORF065R	EAMDCAEI_00067	Uncharacterized protein 042R of IIV3	68516	68995	-	59.1	159	1.08e-62	0
ORF066R	EAMDCAEI_00068	Poxvirus Late Transcription Factor VLTF3 like	69025	70197	-	69.1	388	5.91e-182	0
ORF067L	EAMDCAEI_00069	mRNA-decapping protein D10	70366	71034	+	40.3	216	6.86e-45	0
ORF068L	EAMDCAEI_00070	Uncharacterized protein L5	71537	72913	+	33.8	337	1.58e-38	0
ORF069L*	EAMDCAEI_00071	Uncharacterized protein 088R of IIV3	72919	73668	+	76.5	251	8.12e-144	0
ORF070R	EAMDCAEI_00072	hypothetical protein	73714	74250	-				0
ORF071L	EAMDCAEI_00073	UPF0213 protein CKO_04549	74315	74698	+	55.6	72	1.50e-17	0
ORF072R	EAMDCAEI_00074	Putative serine/threonine-protein kinase 040L of IIV3	74733	75692	-	49.9	335	6.63e-107	0
ORF073L	EAMDCAEI_00075	Uncharacterized protein 045R of IIV3	75843	76127	+	68.1	94	4.11e-36	0
ORF074R	EAMDCAEI_00076	Putative MSV199 domain-containing protein 468L of IIV6	76176	77423	-	43.7	366	1.75e-79	0
ORF075L	EAMDCAEI_00077	Uncharacterized protein 229L of IIV6	77512	78789	+	49.0	418	4.64e-133	0
ORF076L	EAMDCAEI_00078	Uncharacterized protein 378R of IIV6	79217	79921	+	64.3	224	5.84e-70	0
ORF077R	EAMDCAEI_00079	Uncharacterized protein 099R of IIV3	79952	80941	-	59.4	202	7.01e-72	0

ORF078R	EAMDCAEI_00080	Uncharacterized protein 396L of IIV6	80959	83454	-	35.7	931	1.14e-142	0
ORF079R	EAMDCAEI_00081	hypothetical protein	83497	83676	-				1
ORF080L	EAMDCAEI_00082	DNA topoisomerase 2	83912	87304	+	61.2	1129	0.0	0
ORF081L	EAMDCAEI_00083	hypothetical protein	87325	87966	+				0
ORF082R	EAMDCAEI_00084	Uncharacterized protein 063R of IIV3	88005	88679	-	42.7	227	2.51e-47	0
ORF083L	EAMDCAEI_00085	hypothetical protein	88777	89298	+				0
ORF084L	EAMDCAEI_00086	Putative MSV199 domain-containing protein 420R of IIV6	89352	90605	+	35.4	412	1.33e-67	0
ORF085R	EAMDCAEI_00087	hypothetical protein	90646	90861	-				0
ORF086R	EAMDCAEI_00088	Uncharacterized protein 061R of IIV3	90941	92347	-	37.0	478	8.52e-94	0
ORF087L	EAMDCAEI_00089	Thymidylate synthase	92406	93299	+	49.5	293	6.26e-105	0
ORF088L	EAMDCAEI_00090	hypothetical protein	93425	93607	+				0
ORF089L	EAMDCAEI_00091	Uncharacterized protein 028R of IIV3	93718	94314	+	29.9	174	7.99e-21	0
ORF090L*	EAMDCAEI_00092	Putative kinase protein 029R of IIV3	94358	94930	+	57.4	190	5.82e-78	0
ORF091R	EAMDCAEI_00093	uncharacterized protein 030L of IIV3	94969	95352	-	40.5	126	5.08e-19	0
ORF092L	EAMDCAEI_00094	hypothetical protein	96603	97022	+				0
ORF093L	EAMDCAEI_00095	Uncharacterized protein 032R of IIV3	97096	97746	+	51.4	138	4.44e-37	0
ORF094L	EAMDCAEI_00096	protein phosphatase 1, regulatory subunit 15B	97885	98277	+			**6.91e-09**	0
ORF095R*	EAMDCAEI_00097	Uncharacterized protein 033L of IIV3	98292	98855	-	62.4	178	1.26e-83	0
ORF096L	EAMDCAEI_00098	hypothetical protein	98956	99768	+			**1.83e-55**	0
ORF097L	EAMDCAEI_00099	Uncharacterized protein 094L of IIV3	99886	102315	+	37.5	840	2.51e-150	0
ORF098L	EAMDCAEI_00100	Uncharacterized protein 120L of IIV6	102344	102586	+	61.8	55	3.48e-15	0
ORF099L	EAMDCAEI_00101	Uncharacterized protein 053L of IIV3	102619	103038	+	48.2	141	5.23e-40	0
ORF100R	EAMDCAEI_00102	hypothetical protein	103208	103423	-				0
ORF101L	EAMDCAEI_00103	DNA ligase	103569	105398	+	42.7	553	3.07e-123	0
ORF102L	EAMDCAEI_00104	Putative MSV199 domain-containing protein 468L of IIV6	105772	106659	+	39.5	294	7.06e-59	0
ORF103L	EAMDCAEI_00105	Uncharacterized protein 007R of IIV3	106927	108207	+	47.7	449	1.32e-128	0
ORF104L	EAMDCAEI_00106	Putative MSV199 domain-containing protein 468L of IIV6	108303	109451	+	41.6	382	3.51e-85	0
ORF105R	EAMDCAEI_00107	hypothetical protein	109488	109718	-				0
ORF106R	EAMDCAEI_00108	Uncharacterized protein 443R of IIV6	109731	111710	-	45.7	350	8.17e-54	0
ORF107L*	EAMDCAEI_00109	DNA-directed RNA polymerase subunit 2	111774	115145	+	77.3	1137	0.0	0
ORF108L	EAMDCAEI_00110	Uncharacterized protein 404L of IIV6	115156	115890	+	58.7	225	1.73e-89	0
ORF109R	EAMDCAEI_00111	Uncharacterized protein 051L of IIV3	115978	117444	-	31.6	320	8.81e-40	0
ORF110L	EAMDCAEI_00112	hypothetical protein	117487	117783	+				0
ORF111L	EAMDCAEI_00113	hypothetical protein	117866	118201	+	58.7	92	3.92e-33	0
ORF112L*	EAMDCAEI_00114	DNA polymerase family B	118201	122637	+	47.4	1486	0.0	0
ORF113L	EAMDCAEI_00115	Transmembrane protein 049L of IIV6	122715	123029	+	60.8	74	3.16e-23	3
ORF114L*	EAMDCAEI_00116	D5 N terminal like	123135	125954	+	69.6	942	0.0	0
ORF115L	EAMDCAEI_00117	Putative Bro-N domain-containing protein 289L of IIV6	126648	127655	+	52.0	279	1.52e-89	0
ORF116R	EAMDCAEI_00118	Uncharacterized protein 126R of IIV3	127682	127969	-	46.7	105	2.78e-23	2
ORF117R	EAMDCAEI_00119	Uncharacterized protein 125R of IIV3	128016	128879	-	48.6	257	4.40e-90	0

ORF118R	EAMDCAEI_00120	Uncharacterized protein 443R of IIV6	128951	132724	-	43.8	857	3.25e-130	0
ORF119R	EAMDCAEI_00121	Uncharacterized protein 124R of IIV3	132774	133409	-	36.2	229	5.91e-23	0
ORF120L	EAMDCAEI_00122	Uncharacterized protein 123L of IIV3	133428	133826	+	38.6	132	9.42e-20	0
ORF121R	EAMDCAEI_00123	hypothetical protein	133865	134011	-				0
ORF122L	EAMDCAEI_00124	hypothetical protein	134034	134267	+				0
ORF123L	EAMDCAEI_00125	OTU-like cysteine protease	134378	137041	+	60.5	948	4.54e-268	0
ORF124L	EAMDCAEI_00126	hypothetical protein	137090	137590	+			**1.15e-15**	0
ORF125L	EAMDCAEI_00127	Putative MSV199 domain-containing protein 420R of IIV6	137787	139064	+	33.3	420	1.23e-62	0
ORF126L	EAMDCAEI_00128	hypothetical protein	139095	139202	+				1
ORF127L	EAMDCAEI_00129	Uncharacterized protein 082L of IIV3	139267	139710	+	27.5	153	1.60e-13	0
ORF128R*	EAMDCAEI_00130	Erv1/Alr family	139856	140341	-	46.3	108	5.23e-37	1
ORF129L	EAMDCAEI_00131	Probable matrix metalloproteinase 095L of IIV3	140421	141479	+	33.8	311	3.43e-52	0
ORF130R	EAMDCAEI_00132	Uncharacterized protein 317L of IIV6	141507	142616	-	49.0	51	4.30e-06	0
ORF131R	EAMDCAEI_00133	protein tyrosine/serine/threonine phosphatase activity	142670	143389	-	55.5	238	8.08e-92	0
ORF132L	EAMDCAEI_00134	bis(5'-nucleosyl)-tetraphosphatase (symmetrical) activity	143474	144979	+	57.3	337	3.69e-138	0
ORF133R	EAMDCAEI_00135	Uncharacterized protein 073R of IIV3	145108	145662	-	47.6	170	3.80e-44	1
ORF134L	EAMDCAEI_00136	Uncharacterized protein 072L of IIV3	145733	146197	+	59.6	156	5.12e-59	0
ORF135R*	EAMDCAEI_00137	Uncharacterized protein 016R of IIV3	146227	149589	-	49.5	1150	0.0	0
ORF136L	EAMDCAEI_00138	N-methyltransferase activity	150616	151947	+	63.3	286	4.81e-122	0
ORF137L	EAMDCAEI_00139	Putative MSV199 domain-containing protein 420R of IIV6	152023	153276	+	33.5	412	7.03e-63	0
ORF138R	EAMDCAEI_00140	hypothetical protein	153298	153702	-				0
ORF139R	EAMDCAEI_00141	Uncharacterized protein 018L of IIV3	153795	154322	-	47.4	175	2.56e-47	0
ORF140R	EAMDCAEI_00142	hypothetical protein	154535	155269	-				0
ORF141R	EAMDCAEI_00143	Uncharacterized protein 032R of IIV3	155407	156207	-	40.6	133	6.71e-19	0
ORF142L	EAMDCAEI_00144	hypothetical protein	156455	157615	+				0
ORF143L	EAMDCAEI_00145	kinase activity	157714	158223	+			1.25e-68	0
ORF144L	EAMDCAEI_00146	Uncharacterized protein 092R of IIV3	158238	158753	+	64.7	173	4.68e-72	0
ORF145R	EAMDCAEI_00147	Uncharacterized protein 001R of IIV3	158925	159488	-	36.9	187	9.56e-31	0
ORF146L	EAMDCAEI_00148	phosphatase activity	159575	160027	+	63.2	152	2.02e-67	0
ORF147L	EAMDCAEI_00149	Uncharacterized protein 112R of IIV3	160077	160418	+	48.6	111	1.48e-31	1
ORF148R	EAMDCAEI_00150	Trypsin Inhibitor like cysteine rich domain	160461	160688	-	49.1	57	1.01e-16	0
ORF149R	EAMDCAEI_00151	Uncharacterized protein 113L of IIV3	160747	161547	-	44.6	289	4.54e-73	0
ORF150R	EAMDCAEI_00152	Uncharacterized protein 113L of IIV3	161588	163072	-	55.6	493	4.30e-171	0
ORF151L	EAMDCAEI_00153	Putative MSV199 domain-containing protein 238R of IIV6	163121	164641	+	40.1	451	9.61e-87	0
ORF152L	EAMDCAEI_00154	hypothetical protein	164702	165277	+			**1.81e-35**	2
ORF153L	EAMDCAEI_00155	hypothetical protein	165316	166197	+				0
ORF154L*	EAMDCAEI_00156	XPG I-region	166559	167656	+	52.5	377	6.50e-130	0
ORF155L	EAMDCAEI_00157	DNA-directed RNA polymerase subunit 1	168927	169799	+	80.7	274	6.22e-157	0
ORF156L*	EAMDCAEI_00158	RNA polymerase Rpb1, domain 3	169940	172939	+	66.7	1044	0.0	0
ORF157R	EAMDCAEI_00159	hypothetical protein	173045	173293	-				0

ORF158R	EAMDCAEI_00160	High mobility group protein homolog 068R of IIV3	173497	173961	-	82.6	138	2.60e-71	0
ORF159L	EAMDCAEI_00161	Dual specificity phosphatase	174112	174612	+	48.1	133	3.82e-31	0
ORF160L	EAMDCAEI_00162	hypothetical protein	174646	175314	+				0
ORF161R	EAMDCAEI_00163	Uncharacterized protein 119R of IIV3	175362	175856	-	70.6	51	4.63e-20	0
ORF162R	EAMDCAEI_00164	Uncharacterized protein 115R of IIV3	175981	176238	-	60.3	78	5.66e-29	0
ORF163L	EAMDCAEI_00165	hypothetical protein	176373	176804	+				0
ORF164L	EAMDCAEI_00166	Putative FAS1 domain-containing protein 081L of IIV3	176816	177382	+	34.0	188	3.21e-26	0
ORF165L	EAMDCAEI_00167	Probable cysteine proteinase 024R of IIV3	177461	178906	+	55.9	485	1.88e-196	1
ORF166L	EAMDCAEI_00168	hypothetical protein	178949	179353	+				0
ORF167R	EAMDCAEI_00169	Uncharacterized protein 422L of IIV6	179386	179970	-	42.9	175	1.50e-34	0
ORF168L	EAMDCAEI_00170	Uncharacterized protein 017R of IIV3	180096	180401	+	51.7	89	1.06e-18	0
ORF169L	EAMDCAEI_00171	Uncharacterized protein 017R of IIV3	180398	180919	+	55.4	175	9.33e-59	0
ORF170L	EAMDCAEI_00172	Putative MSV199 domain-containing protein 468L of IIV6	181313	182605	+	39.3	356	6.10e-78	0
ORF171L	EAMDCAEI_00173	Uncharacterized protein 107R of IIV3	182786	183595	+	52.4	231	9.02e-69	0
ORF172L	EAMDCAEI_00174	Putative MSV199 domain-containing protein 420R of IIV6	184047	184316	+	37.6	85	2.55e-12	0
ORF173R	EAMDCAEI_00175	Uncharacterized protein 023R of IIV3	184920	185204	-	72.3	94	9.61e-45	0
ORF174L	EAMDCAEI_00176	Uncharacterized protein 050L of IIV3	185279	185773	+	61.4	140	2.77e-53	0
ORF175R	EAMDCAEI_00177	hypothetical protein	185834	186319	-				0
ORF176L*	EAMDCAEI_00178	Ribonucleoside-diphosphate reductase small chain	188228	189370	+	80.2	328	1.46e-196	0
ORF177L	EAMDCAEI_00179	hypothetical protein	189880	190392	+				1
ORF178L	EAMDCAEI_00180	Uncharacterized protein 026R of IIV3	190370	191104	+	64.9	225	2.67e-100	0
ORF179L	EAMDCAEI_00181	Putative RING finger protein 027R of IIV3	191179	191628	+	30.5	154	5.83e-12	0
ORF180R	EAMDCAEI_00182	hypothetical protein	191691	192095	-				0
ORF181L*	EAMDCAEI_00183	Uncharacterized protein 004R of IIV3	192154	193386	+	56.2	450	6.33e-139	0
ORF182R	EAMDCAEI_00184	hypothetical protein	193409	194242	-				0
ORF183L*	EAMDCAEI_00185	Putative myristoylated protein 006R of IIV3	194400	195941	+	56.2	493	5.98e-192	3
ORF184L	EAMDCAEI_00001	Uncharacterized protein 404L of IIV6	196373	197104	+	37.0	211	1.32e-29	0
ORF185R	EAMDCAEI_00002	Uncharacterized 15.9 kDa protein in MSP 5'region	197136	197549	-	52.8	106	1.99e-28	0

*ORFs labeled with an asterisk denote core genes recovered and validated with VIGA across all five sequenced IIV genomes from lepidopteran hosts.

E-values: Gene annotation by Blast (normal text) or eggNOG-mapper (bold text).

TM domain content – transmembrane domains (alpha-helices) detected by using DeepTMHMM.
