# Supplementary material for: Invertebrate Iridescent Viruses (Iridoviridae) from the Fall Armyworm, Spodoptera frugiperda
Source: Viruses. 2025 Dec 24;18(1):31. doi: 10.3390/v18010031 (PMC12846554; doi:10.3390/v18010031)
Supplement: Supplementary file 1 [file viruses-18-00031-s001.zip › Table_S7.pdf]

Table S7. SfIIV-Ver genome annotation

ORF	Locus tag	Product	Start	End	Strand	Identity (%)	Alignment length (bp)	E-Value	TM domain count
ORF001L*	GLJAONHD_00119	Major capsid protein	1	1386	+	95.8	454	2.12e-318	0
ORF002R	GLJAONHD_00120	Uncharacterized protein 273R of IIV6	1477	2964	-	43.7	142	1.77e-25	0
ORF003L	GLJAONHD_00121	Uncharacterized protein 009R of IIV6	3029	3289	+	59.3	81	7.08e-34	0
ORF004L	GLJAONHD_00122	hypothetical protein	3337	3561	+				0
ORF005L	GLJAONHD_00123	hypothetical protein	3558	3875	+				0
ORF006L	GLJAONHD_00124	Uncharacterized protein IIV3-013L	3927	4190	+	49.5	91	4.26e-20	1
ORF007L*	GLJAONHD_00125	Uncharacterized protein 035R of IIV3	4294	7503	+	51.3	1103	0.0	0
ORF008L*	GLJAONHD_00126	Putative transcription elongation factor S-II-like protein 055R of IIV3	7551	7973	+	46.8	139	1.74e-42	0
ORF009L	GLJAONHD_00127	Putative Bro-N domain-containing protein 019R of IIV3	8234	9625	+	55.1	285	6.32e-89	0
ORF010R	GLJAONHD_00128	Uncharacterized protein 069L of IIV3	9788	11059	-	46.1	425	4.05e-118	0
ORF011L	GLJAONHD_00129	hypothetical protein	11106	11996	+			**2.31e-112**	0
ORF012L	GLJAONHD_00130	hypothetical protein	12049	12486	+				0
ORF013L*	GLJAONHD_00131	Uncharacterized protein 056L of IIV3	12633	13652	+	43.8	345	3.46e-88	0
ORF014R*	GLJAONHD_00132	Probable serine/threonine-protein kinase 380R of IIV6	13699	15267	-	41.3	545	2.01e-115	0
ORF015L*	GLJAONHD_00133	Putative zinc finger protein 012R of IIV3	15433	16578	+	43.2	382	1.72e-101	0
ORF016R	GLJAONHD_00134	Uncharacterized protein 085L of IIV3	17028	17498	-	65.0	137	2.53e-63	1
ORF017L	GLJAONHD_00135	Uncharacterized protein 054L of IIV3	17667	18497	+	49.6	240	5.60e-75	0
ORF018L	GLJAONHD_00136	Uncharacterized protein 102R of IIV3	18595	18948	+	59.1	115	2.52e-35	0
ORF019L*	GLJAONHD_00137	Putative membrane protein 047R of IIV3	19009	20319	+	76.3	274	4.42e-170	2
ORF020R	GLJAONHD_00138	Putative MSV199 domain-containing protein 420R of IIV6	20575	21828	-	31.8	412	1.89e-57	0
ORF021L	GLJAONHD_00139	hypothetical protein	22000	22851	+				0
ORF022L*	GLJAONHD_00140	ribonuclease III activity	22945	23781	+	76.4	280	2.67e-148	0
ORF023L	GLJAONHD_00141	Transmembrane protein 022L of IIV3	23993	24622	+	51.7	172	3.17e-55	6
ORF024L	GLJAONHD_00142	Zinc finger	24685	25350	+	51.3	228	4.22e-62	0
ORF025R*	GLJAONHD_00143	Putative CTD phosphatase-like protein 355R of IIV3	25379	25939	-	63.8	185	5.09e-81	0
ORF026L	GLJAONHD_00144	Uncharacterized protein 105R of IIV3	26062	26793	+	65.0	246	2.46e-112	0
ORF027R	GLJAONHD_00145	Uncharacterized protein 159L of IIV6	26838	28319	-	32.5	231	1.00e-25	0
ORF028R	GLJAONHD_00146	Uncharacterized protein 159L of IIV6	28404	29879	-	33.5	233	1.65e-28	0
ORF029L*	GLJAONHD_00147	Uncharacterized protein 106R of IIV3	30011	31444	+	61.9	465	8.17e-207	0
ORF030R	GLJAONHD_00148	Uncharacterized protein 071L of IIV3	31575	32219	-	69.6	194	2.29e-85	0
ORF031L	GLJAONHD_00149	Uncharacterized protein 020R of IIV3	33131	33628	+	57.7	163	2.34e-62	0
ORF032R	GLJAONHD_00150	Putative MSV199 domain-containing protein 420R of IIV6	33736	33852	-	58.1	31	1.16e-07	0
ORF033R	GLJAONHD_00151	Uncharacterized protein 97L of IIV3	33879	34481	-	55.2	203	9.20e-73	0
ORF034L	GLJAONHD_00152	dUTPase	34613	35182	+	47.9	140	1.06e-34	1
ORF035L	GLJAONHD_00153	Putative MSV199 domain-containing protein 468L of IIV6	35280	36572	+	42.1	356	1.44e-85	0
ORF036L	GLJAONHD_00154	Dihydrofolate reductase	36605	37156	+	37.6	178	7.13e-38	0

ORF037R	GLJAONHD_00155	hypothetical protein	37145	37408	-				3
ORF038L	GLJAONHD_00156	XRN 5'-3' exonuclease N-terminus	37583	39277	+	63.4	571	3.79e-261	0
ORF039L	GLJAONHD_00157	hypothetical protein	39393	39890	+				0
ORF040L	GLJAONHD_00158	hypothetical protein	39996	40136	+				0
ORF041R	GLJAONHD_00159	Uncharacterized protein 058R of IIV3	40177	40608	-	67.4	138	6.76e-67	0
ORF042R	GLJAONHD_00160	hypothetical protein	40669	41067	-				0
ORF043L	GLJAONHD_00161	Uncharacterized protein 060L of IIV3	41234	42004	+	53.4	264	3.48e-83	0
ORF044L	GLJAONHD_00162	hypothetical protein	42176	42382	+				0
ORF045L	GLJAONHD_00163	Putative MSV199 domain-containing protein 238R of IIV6	42431	43888	+	41.9	442	7.26e-97	0
ORF046R	GLJAONHD_00164	N-methyltransferase activity	43928	46837	-	62.6	984	0.0	0
ORF047L	GLJAONHD_00165	Putative Bro-N domain-containing protein 019R of IIV3	47054	48406	+	50.0	342	6.09e-95	0
ORF048L	GLJAONHD_00166	Putative SWIB domain-containing protein 070L of IIV3	48541	49278	+	55.9	229	6.01e-73	0
ORF049R*	GLJAONHD_00167	protein serine/threonine kinase activity	49317	50852	-	58.8	512	4.97e-209	0
ORF050L	GLJAONHD_00168	Immediate-early protein ICP-46 homolog	50955	52289	+	54.0	441	3.21e-156	0
ORF051L	GLJAONHD_00169	Double-stranded RNA binding motif	52437	52832	+			**3.12e-49**	0
ORF052L	GLJAONHD_00170	hypothetical protein	52889	53044	+				0
ORF053L	GLJAONHD_00171	Putative thioredoxin-like protein 041R of IIV3	53072	53428	+	55.1	118	9.24e-47	0
ORF054R	GLJAONHD_00172	hypothetical protein	53469	54032	-				1
ORF055L*	GLJAONHD_00173	Uncharacterized protein 038R of IIV3	54097	55749	+	54.3	549	6.93e-206	0
ORF056R	GLJAONHD_00174	Uncharacterized protein 043R of IIV3	55958	56149	-	74.6	63	2.09e-32	2
ORF057R	GLJAONHD_00175	Uncharacterized protein 443R of IIV6	56164	63018	-	32.3	1742	8.58e-119	0
ORF058L	GLJAONHD_00176	Uncharacterized protein 074L of IIV3	63121	65217	+	45.4	808	1.36e-215	0
ORF059L	GLJAONHD_00177	Ribonucleotide reductase	65336	67675	+	57.1	785	5.36e-300	0
ORF060L	GLJAONHD_00178	hypothetical protein	67787	68269	+			3.00e-09	0
ORF061R	GLJAONHD_00179	Uncharacterized protein 042R of IIV3	68298	68777	-	59.1	159	1.08e-62	0
ORF062R	GLJAONHD_00180	Poxvirus Late Transcription Factor VLTF3 like	68807	69979	-	69.1	388	5.91e-182	0
ORF063L	GLJAONHD_00181	mRNA-decapping protein D10	70149	70817	+	40.3	216	1.37e-44	0
ORF064L	GLJAONHD_00182	Uncharacterized protein L5	71305	72702	+	33.5	337	3.72e-40	0
ORF065L*	GLJAONHD_00183	Uncharacterized protein 088R of IIV3	72699	73457	+	76.3	257	2.25e-144	0
ORF066R	GLJAONHD_00184	hypothetical protein	73503	74039	-				0
ORF067R	GLJAONHD_00001	Putative serine/threonine-protein kinase 040L of IIV3	74511	75470	-	49.7	332	3.79e-106	0
ORF068L	GLJAONHD_00002	Uncharacterized protein 045R of IIV3	75620	75904	+	68.1	94	8.30e-36	0
ORF069R	GLJAONHD_00003	Putative MSV199 domain-containing protein 468L of IIV6	75976	76974	-	42.2	344	1.31e-67	0
ORF070L	GLJAONHD_00004	Uncharacterized protein 229L of IIV6	77138	78415	+	48.6	418	2.65e-132	0
ORF071L	GLJAONHD_00005	Uncharacterized protein 443R of IIV6	78461	82369	+	39.0	1110	2.5e-136	0
ORF072L	GLJAONHD_00006	Uncharacterized protein 378R of IIV6	82435	83139	+	63.8	224	1.66e-69	0
ORF073R	GLJAONHD_00007	Uncharacterized protein 099R of IIV3	83170	84159	-	59.4	202	7.01e-72	0
ORF074R	GLJAONHD_00008	Uncharacterized protein 396L of IIV6	84177	86672	-	35.8	931	3.31e-141	0
ORF075R	GLJAONHD_00009	hypothetical protein	86715	86894	-				1
ORF076L	GLJAONHD_00010	DNA topoisomerase 2	87130	90522	+	61.1	1129	0.0	0

ORF077L	GLJAONHD_00011	hypothetical protein	90543	91184	+				0
ORF078R	GLJAONHD_00012	Uncharacterized protein 063R of IIV3	91223	91897	-	42.7	227	2.51e-47	0
ORF079L	GLJAONHD_00013	hypothetical protein	91995	92516	+				0
ORF080L	GLJAONHD_00014	Putative MSV199 domain-containing protein 420R of IIV6	92563	93816	+	35.4	412	9.48e-68	0
ORF081R	GLJAONHD_00015	hypothetical protein	93857	94072	-				0
ORF082R	GLJAONHD_00016	Uncharacterized protein 061R of IIV3	94151	94510	-	42.4	118	1.17e-24	0
ORF083R	GLJAONHD_00017	Uncharacterized protein 061R of IIV3	95052	95555	-	34.1	173	8.74e-21	0
ORF084L	GLJAONHD_00018	Thymidylate synthase	95615	96508	+	49.3	296	8.87e-105	0
ORF085L	GLJAONHD_00019	hypothetical protein	96634	96816	+				0
ORF086L	GLJAONHD_00020	Uncharacterized protein 028R of IIV3	96927	97523	+	29.9	174	7.99e-21	0
ORF087L*	GLJAONHD_00021	Putative kinase protein 029R of IIV3	97567	98139	+	57.4	190	5.82e-78	0
ORF088R	GLJAONHD_00022	uncharacterized protein 030L of IIV3	98178	98558	-	40.5	126	1.23e-19	0
ORF089R	GLJAONHD_00023	Uncharacterized protein 261R of IIV6	98571	99797	-	55.3	170	3.51e-45	0
ORF090L	GLJAONHD_00024	hypothetical protein	100577	100996	+				0
ORF091L	GLJAONHD_00025	Uncharacterized protein 032R of IIV3	101070	101789	+	51.4	138	9.05e-37	0
ORF092L	GLJAONHD_00026	protein phosphatase 1	101863	102198	+			**5.58e-10**	0
ORF093L	GLJAONHD_00027	Putative MSV199 domain-containing protein 468L of IIV6	102290	103552	+	41.0	361	7.07e-77	0
ORF094R*	GLJAONHD_00028	Uncharacterized protein 033L of IIV3	103583	104146	-	62.4	178	6.27e-84	0
ORF095L	GLJAONHD_00029	hypothetical protein	104247	105044	+			**2.43e-57**	0
ORF096L	GLJAONHD_00030	Uncharacterized protein 094L of IIV6	105156	107585	+	37.6	840	1.78e-150	0
ORF097L	GLJAONHD_00031	Uncharacterized protein 120L of IIV6	107614	107856	+	60.7	56	2.46e-15	0
ORF098L	GLJAONHD_00032	Uncharacterized protein 053L of IIV3	107889	108308	+	48.9	141	1.83e-40	0
ORF099R	GLJAONHD_00033	hypothetical protein	108478	108693	-				0
ORF100L	GLJAONHD_00034	DNA ligase	108833	110662	+	42.5	553	2.18e-123	0
ORF101L	GLJAONHD_00035	Putative MSV199 domain-containing protein 468L of IIV6	110753	112024	+	40.8	373	1.03e-83	0
ORF102L	GLJAONHD_00036	Uncharacterized protein 007R of IIV3	112109	113389	+	47.7	449	1.32e-128	0
ORF103L	GLJAONHD_00037	Putative MSV199 domain-containing protein 468L of IIV6	113485	114657	+	43.5	382	2.55e-87	0
ORF104R	GLJAONHD_00038	hypothetical protein	114694	114924	-				0
ORF105R	GLJAONHD_00039	Uncharacterized protein 443R of IIV6	114937	116916	-	45.1	350	1.48e-53	0
ORF106L*	GLJAONHD_00040	DNA-directed RNA polymerase subunit 2	116980	120351	+	77.3	1137	0.0	0
ORF107L	GLJAONHD_00041	Uncharacterized protein 404L of IIV6	120362	121093	+	63.9	216	1.82e-98	0
ORF108R	GLJAONHD_00042	Uncharacterized protein 051L of IIV3	121178	122644	-	31.6	320	8.81e-40	0
ORF109L	GLJAONHD_00043	hypothetical protein	122689	122985	+				0
ORF110L	GLJAONHD_00044	hypothetical protein	123067	123384	+	60.9	87	1.99e-32	0
ORF111L*	GLJAONHD_00045	DNA polymerase family B	123401	126760	+	62.6	1127	0.0	0
ORF112L	GLJAONHD_00046	Transmembrane protein 049L of IIV6	126838	127152	+	60.8	74	3.17e-23	3
ORF113L*	GLJAONHD_00047	D5 N terminal like	127259	130078	+	69.7	941	0.0	0
ORF114L	GLJAONHD_00048	Putative MSV199 domain-containing protein 420R of IIV6	130530	131798	+	40.3	412	5.26e-90	0
ORF115R	GLJAONHD_00049	Uncharacterized protein 126R of IIV3	131829	132116	-	45.7	105	7.95e-23	2
ORF116R	GLJAONHD_00050	Uncharacterized protein 125R of IIV3	132163	133026	-	49.4	257	1.09e-90	0

ORF117R	GLJAONHD_00051	Uncharacterized protein 443R of IIV6	133065	136823	-	42.7	857	1.98e-124	0
ORF118R	GLJAONHD_00052	Uncharacterized protein 124R of IIV3	136873	137508	-	36.2	224	3.02e-23	0
ORF119L	GLJAONHD_00053	Uncharacterized protein 123L of IIV3	137527	137925	+	38.6	132	1.18e-20	0
ORF120R	GLJAONHD_00054	hypothetical protein	137964	138110	-				0
ORF121L	GLJAONHD_00055	hypothetical protein	138133	138363	+				0
ORF122L	GLJAONHD_00056	OTU-like cysteine protease	138567	141398	+	64.1	802	9.36e-261	0
ORF123L	GLJAONHD_00057	hypothetical protein	141447	141947	+			**1.15e-15**	0
ORF124L	GLJAONHD_00058	Putative MSV199 domain-containing protein 420R of IIV6	142090	143343	+	32.8	412	1.38e-62	0
ORF125L	GLJAONHD_00059	Uncharacterized protein 082L of IIV3	143546	143989	+	27.5	153	6.19e-13	0
ORF126R*	GLJAONHD_00060	Erv1 / Alr family	144135	144596	-	46.3	108	3.93e-37	1
ORF127L	GLJAONHD_00061	Probable matrix metalloproteinase 095L of IIV3	144676	145764	+	33.8	311	3.23e-52	0
ORF128R	GLJAONHD_00062	hypothetical protein	145792	146901	-				0
ORF129R	GLJAONHD_00063	protein tyrosine/serine/threonine phosphatase activity	146956	147675	-	55.5	238	8.08e-92	0
ORF130L	GLJAONHD_00064	bis(5'-nucleosyl)-tetraphosphatase (symmetrical) activity	147759	149264	+	57.6	337	2.61e-138	0
ORF131R	GLJAONHD_00065	Uncharacterized protein 073R of IIV3	149405	149959	-	45.2	177	6.67e-45	1
ORF132L	GLJAONHD_00066	Uncharacterized protein 072L of IIV3	150048	150512	+	59.6	156	2.08e-58	0
ORF133R*	GLJAONHD_00067	Uncharacterized protein 016R of IIV3	150542	153904	-	49.6	1150	0.0	0
ORF134L	GLJAONHD_00068	N-methyltransferase activity	154932	156263	+	63.3	286	4.81e-122	0
ORF135L	GLJAONHD_00069	Putative MSV199 domain-containing protein 420R of IIV6	156337	157587	+	32.5	412	1.63e-59	0
ORF136R	GLJAONHD_00070	hypothetical protein	157609	158013	-				0
ORF137R	GLJAONHD_00071	Uncharacterized protein 018L of IIV3	158108	158635	-	47.4	175	2.56e-47	0
ORF138R	GLJAONHD_00072	hypothetical protein	158847	159584	-				0
ORF139R	GLJAONHD_00073	Uncharacterized protein 032R of IIV3	159722	160591	-	38.4	146	7.5e-19	0
ORF140L	GLJAONHD_00074	hypothetical protein	160839	162116	+				0
ORF141L	GLJAONHD_00075	kinase activity	162200	162709	+			**1.78e-68**	0
ORF142L	GLJAONHD_00076	Uncharacterized protein 092R of IIV3	162724	163239	+	64.7	173	4.68e-72	0
ORF143R	GLJAONHD_00077	Uncharacterized protein 001R of IIV3	163411	163974	-	36.9	187	9.56e-31	0
ORF144L	GLJAONHD_00078	phosphatase activity	164062	164514	+	63.2	152	1.00e-67	0
ORF145L	GLJAONHD_00079	Uncharacterized protein 112R of IIV3	164564	164926	+	44.3	115	5.57e-26	1
ORF146R	GLJAONHD_00080	Trypsin Inhibitor like cysteine rich domain	164969	165196	-	49.1	57	1.01e-16	0
ORF147L	GLJAONHD_00081	hypothetical protein	165240	165422	+				0
ORF148R	GLJAONHD_00082	Uncharacterized protein 113L of IIV3	165697	168024	-	51.6	803	4.64e-270	0
ORF149L	GLJAONHD_00083	Putative MSV199 domain-containing protein 468L of IIV6	168129	169241	+	39.9	378	3.36e-82	0
ORF150L	GLJAONHD_00084	hypothetical protein	169475	169885	+			**9.20e-37**	0
ORF151L	GLJAONHD_00085	hypothetical protein	169924	170994	+				0
ORF152L*	GLJAONHD_00086	XPG I-region	171153	172250	+	52.5	377	7.47e-129	0
ORF153L*	GLJAONHD_00087	RNA polymerase Rpb1	173128	177138	+	70.5	1375	0.0	0
ORF154L	GLJAONHD_00088	hypothetical protein	177178	177660	+				0
ORF155R	GLJAONHD_00089	hypothetical protein	177726	177974	-				0
ORF156R	GLJAONHD_00090	High mobility group protein homolog 068R of IIV3	178178	178642	-	82.6	138	2.60e-71	0

ORF157L	GLJAONHD_00091	Dual specificity phosphatase	178793	179293	+	47.8	134	1.69e-31	0
ORF158L	GLJAONHD_00092	hypothetical protein	179327	179986	+				0
ORF159R	GLJAONHD_00093	Uncharacterized protein 119R of IIV3	180034	180546	-	70.6	51	5.52e-20	0
ORF160R	GLJAONHD_00094	Uncharacterized protein 115R of IIV3	180660	180917	-	60.3	78	5.66e-29	0
ORF161L	GLJAONHD_00095	hypothetical protein	181052	181483	+				0
ORF162L	GLJAONHD_00096	Putative FAS1 domain-containing protein 081L of IIV3	181495	182061	+	34.4	192	3.21e-26	0
ORF163L	GLJAONHD_00097	Probable cysteine proteinase 024R of IIV3	182140	183585	+	56.1	485	9.34e-197	1
ORF164L	GLJAONHD_00098	hypothetical protein	183628	184032	+				0
ORF165R	GLJAONHD_00099	Uncharacterized protein 422L of IIV6	184070	184654	-	42.9	175	1.5e-34	0
ORF166L	GLJAONHD_00100	Uncharacterized protein 017R of IIV3	184780	185175	+	51.7	89	2.18e-18	0
ORF167L	GLJAONHD_00101	Uncharacterized protein 017R of IIV3	185172	185804	+	59.4	212	9.70e-82	0
ORF168L	GLJAONHD_00102	Putative MSV199 domain-containing protein 468L of IIV6	186094	187383	+	41.4	355	1.38e-82	0
ORF169L	GLJAONHD_00103	Uncharacterized protein 107R of IIV3	187461	188387	+	46.0	309	2.30e-79	0
ORF170L	GLJAONHD_00104	Putative Bro-N domain-containing protein 019R of IIV3	188638	189957	+	53.2	312	4.81e-97	0
ORF171R	GLJAONHD_00105	Uncharacterized protein 023R of IIV3	189984	190268	-	72.3	94	9.61e-45	0
ORF172L	GLJAONHD_00106	Uncharacterized protein 050L of IIV3	190343	190813	+	62.1	145	3.98e-58	0
ORF173R	GLJAONHD_00107	Uncharacterized protein 049R of IIV3	190854	192392	-	42.5	113	1.32e-18	0
ORF174L*	GLJAONHD_00108	Ribonucleoside-diphosphate reductase small chain	192501	193604	+	76.4	351	4.81e-201	0
ORF175L	GLJAONHD_00109	hypothetical protein	193870	194382	+				1
ORF176L	GLJAONHD_00110	Uncharacterized protein 026R of IIV3	194360	195094	+	64.9	225	6.59e-101	0
ORF177L	GLJAONHD_00111	Putative RING finger protein 027R of IIV3	195167	195616	+	29.2	154	1.19e-10	0
ORF178R	GLJAONHD_00112	hypothetical protein	195680	196075	-				0
ORF179L*	GLJAONHD_00113	Uncharacterized protein 004R of IIV3	196134	197351	+	55.8	450	5.28e-139	0
ORF180R	GLJAONHD_00114	hypothetical protein	197374	198210	-				0
ORF181L*	GLJAONHD_00115	Putative myristoylated protein 006R of IIV3	198367	199908	+	56.2	493	5.98e-192	3
ORF182L	GLJAONHD_00116	hypothetical protein	199925	200272	+			**3.12e-18**	2
ORF183L	GLJAONHD_00117	Uncharacterized protein 404L of IIV6	200340	201074	+	36.0	211	6.89e-30	0
ORF184R	GLJAONHD_00118	Uncharacterized 15.9 kDa protein in MSP 5'region	201106	201519	-	52.8	106	5.65e-28	0

*ORFs labeled with an asterisk denote core genes recovered and validated with VIGA across all five sequenced IIV genomes from lepidopteran hosts.
E-values: Gene annotation by Blast (normal text) or eggNOG-mapper (bold text).
TM domain content – transmembrane domains (alpha-helices) detected by using DeepTMHMM.
