# Supplementary material for: Invertebrate Iridescent Viruses (Iridoviridae) from the Fall Armyworm, Spodoptera frugiperda
Source: Viruses. 2025 Dec 24;18(1):31. doi: 10.3390/v18010031 (PMC12846554; doi:10.3390/v18010031)
Supplement: Supplementary file 1 [file viruses-18-00031-s001.zip › Table_S8.pdf]

**Table S8.** SfIIV-Arg genome annotation

| ORF notation | Locus tag | Product | Start | End | Strand | Identity (%) | Alignment length (bp) | E-Value | TM domain count |
|---|---|---|---|---|---|---|---|---|---|
| ORF001R* | CGAJLDHN_00162 | Major capsid protein | 1 | 1386 | - | 95.6 | 454 | 2.12e-317 | 0 |
| ORF002L | CGAJLDHN_00163 | Uncharacterized 15.9 kDa protein in MSP 5'region | 1498 | 1911 | + | 52.8 | 106 | 3.99e-28 | 0 |
| ORF003R | CGAJLDHN_00164 | hypothetical protein | 1943 | 2707 | - | | | | 0 |
| ORF004R | CGAJLDHN_00165 | hypothetical protein | 2776 | 3123 | - | | | **1.55e-18** | 2 |
| ORF005R* | CGAJLDHN_00166 | Putative myristoylated protein 006R of IIV3 | 3140 | 4681 | - | 56.4 | 493 | 7.36e-193 | 3 |
| ORF006L | CGAJLDHN_00167 | hypothetical protein | 4839 | 5687 | + | | | | 0 |
| ORF007R | CGAJLDHN_00168 | Uncharacterized protein 004R of IIV3 | 5709 | 5894 | - | 50.8 | 65 | 3.52e-08 | 0 |
| ORF008R* | CGAJLDHN_00169 | Uncharacterized protein 004R of IIV3 | 6001 | 6669 | - | 59.3 | 221 | 1.86e-66 | 0 |
| ORF009R | CGAJLDHN_00170 | Uncharacterized protein 004R of IIV3 | 6635 | 6976 | - | 54.1 | 74 | 4.20e-17 | 0 |
| ORF010L | CGAJLDHN_00171 | hypothetical protein | 7035 | 7466 | + | | | | 0 |
| ORF011R | CGAJLDHN_00172 | Putative RING finger protein 027R of IIV3 | 7521 | 7922 | - | 27.8 | 144 | 1.15e-08 | 0 |
| ORF012R | CGAJLDHN_00173 | Uncharacterized protein 026R of IIV3 | 8035 | 8334 | - | 68.6 | 86 | 4.59e-34 | 0 |
| ORF013R | CGAJLDHN_00174 | Uncharacterized protein 026R of IIV3 | 8276 | 8710 | - | 57.2 | 138 | 1.41e-46 | 0 |
| ORF014R | CGAJLDHN_00175 | hypothetical protein | 8741 | 9253 | - | | | | 1 |
| ORF015R* | CGAJLDHN_00176 | Ribonucleoside-diphosphate reductase small chain | 9774 | 10802 | - | 79.1 | 340 | 6.28e-202 | 0 |
| ORF016L | CGAJLDHN_00177 | hypothetical protein | 10996 | 11589 | + | | | | 0 |
| ORF017L | CGAJLDHN_00178 | hypothetical protein | 11712 | 11918 | + | | | | 0 |
| ORF018L | CGAJLDHN_00179 | hypothetical protein | 12960 | 13115 | + | | | | 0 |
| ORF019R | CGAJLDHN_00180 | Uncharacterized protein 050L of IIV3 | 13629 | 13772 | - | 60.5 | 43 | 1.31e-12 | 0 |
| ORF020R | CGAJLDHN_00181 | Uncharacterized protein 050L of IIV3 | 13769 | 14026 | - | 58.4 | 77 | 1.63e-23 | 0 |
| ORF021R | CGAJLDHN_00182 | Putative Bro-N domain-containing protein 019R of IIV3 | 14528 | 15085 | - | 62.1 | 161 | 7.48e-65 | 0 |
| ORF022R | CGAJLDHN_00183 | Putative MSV199 domain-containing protein 420R of IIV6 | 15082 | 15330 | - | 47.0 | 66 | 2.67e-12 | 0 |
| ORF023R | CGAJLDHN_00184 | Putative MSV199 domain-containing protein 420R of IIV6 | 15330 | 15572 | - | 44.9 | 49 | 2.34e-06 | 0 |
| ORF024R | CGAJLDHN_00185 | Uncharacterized protein 107R of IIV3 | 15811 | 16239 | - | 52.0 | 102 | 3.72e-21 | 0 |
| ORF025R | CGAJLDHN_00186 | Uncharacterized protein 107R of IIV3 | 16450 | 16731 | - | 46.7 | 60 | 1.17e-10 | 0 |
| ORF026R | CGAJLDHN_00187 | Putative MSV199 domain-containing protein 212L of IIV6 | 17144 | 17596 | - | 37.6 | 149 | 8.84e-21 | 0 |
| ORF027R | CGAJLDHN_00188 | acid-amino acid ligase activity | 17762 | 18100 | - | | | **2.32e-21** | 0 |
| ORF028R | CGAJLDHN_00189 | Uncharacterized protein 017R of IIV3 | 18511 | 18687 | - | 65.5 | 55 | 0 | 0 |
| ORF029R | CGAJLDHN_00190 | hypothetical protein | 18663 | 18782 | - | | | | 0 |
| ORF030R | CGAJLDHN_00191 | Uncharacterized protein 017R of IIV3 | 18815 | 19096 | - | 50.0 | 74 | 2.95e-13 | 0 |
| ORF031R | CGAJLDHN_00192 | hypothetical protein | 19198 | 19290 | - | | | | 0 |
| ORF032L | CGAJLDHN_00193 | Uncharacterized protein 422L of IIV6 | 19416 | 19916 | + | 43.7 | 167 | 6.6e-34 | 0 |
| ORF033R | CGAJLDHN_00194 | hypothetical protein | 20099 | 20440 | - | | | | 0 |
| ORF034R | CGAJLDHN_00195 | Probable cysteine proteinase 024R of IIV3 | 20483 | 21922 | - | 55.0 | 487 | 2.68e-193 | 1 |
| ORF035R | CGAJLDHN_00196 | Putative FAS1 domain-containing protein 081L of IIV3 | 22001 | 22567 | - | 33.5 | 188 | 3.55e-25 | 0 |
| ORF036L | CGAJLDHN_00001 | Uncharacterized protein 115R of IIV3 | 23143 | 23400 | + | 60.3 | 78 | 5.66e-29 | 0 |

| | | | | | | | | | |
|---|---|---|---|---|---|---|---|---|---|
| ORF037L | CGAJLDHN_00002 | Uncharacterized protein 119R of IIV3 | 23525 | 24019 | + | 70.6 | 51 | 4.63e-20 | 0 |
| ORF038R | CGAJLDHN_00003 | hypothetical protein | 24067 | 24741 | - | | | | 0 |
| ORF039R | CGAJLDHN_00004 | hypothetical protein | 24775 | 25275 | - | 46.6 | 133 | 3.38e-32 | 0 |
| ORF040L | CGAJLDHN_00005 | High mobility group protein homolog 068R of IIV3 | 25426 | 25890 | + | 81.3 | 139 | 2.6e-71 | 0 |
| ORF041L | CGAJLDHN_00006 | hypothetical protein | 26093 | 26341 | + | | | | 0 |
| ORF042R* | CGAJLDHN_00007 | RNA polymerase Rpb1, domain 3 | 26447 | 29446 | - | 66.3 | 1044 | 0.0 | 0 |
| ORF043R | CGAJLDHN_00008 | DNA-directed RNA polymerase subunit 1 | 29587 | 30456 | - | 80.7 | 274 | 5.97e-157 | 0 |
| ORF044R* | CGAJLDHN_00009 | XPG I-region | 31344 | 32441 | - | 52.8 | 377 | 8.01e-131 | 0 |
| ORF045R | CGAJLDHN_00010 | hypothetical protein | 32804 | 33667 | - | | | | 0 |
| ORF046R | CGAJLDHN_00011 | hypothetical protein | 33706 | 34281 | - | | | **6.36e-36** | 2 |
| ORF047R | CGAJLDHN_00012 | Putative MSV199 domain-containing protein 468L of IIV6 | 34342 | 35493 | - | 40.1 | 364 | 1.43e-78 | 0 |
| ORF048L | CGAJLDHN_00013 | Uncharacterized protein 113L of IIV3 | 35599 | 37926 | + | 51.4 | 803 | 9.32e-270 | 0 |
| ORF049L | CGAJLDHN_00014 | Trypsin Inhibitor like cysteine rich domain | 38012 | 38239 | + | 49.1 | 57 | 1.01e-16 | 0 |
| ORF050R | CGAJLDHN_00015 | Uncharacterized protein 112R of IIV3 | 38282 | 38623 | - | 48.6 | 111 | 6.00e-31 | 1 |
| ORF051R | CGAJLDHN_00016 | phosphatase activity | 38673 | 39125 | - | 62.5 | 152 | 1.65e-66 | 0 |
| ORF052L | CGAJLDHN_00017 | Uncharacterized protein 001R of IIV3 | 39215 | 39778 | + | 36.4 | 187 | 3.04e-29 | 0 |
| ORF053R | CGAJLDHN_00018 | Uncharacterized protein 092R of IIV3 | 39952 | 40467 | - | 65.3 | 173 | 1.63e-72 | 0 |
| ORF054R | CGAJLDHN_00019 | kinase activity | 40482 | 40991 | - | | | **3.58e-68** | 0 |
| ORF055R | CGAJLDHN_00020 | hypothetical protein | 41089 | 42261 | - | | | | 0 |
| ORF056L | CGAJLDHN_00021 | Uncharacterized protein 032R of IIV3 | 42509 | 43291 | + | 39.8 | 133 | 2.18e-18 | 0 |
| ORF057L | CGAJLDHN_00022 | hypothetical protein | 43430 | 44176 | + | | | | 0 |
| ORF058L | CGAJLDHN_00023 | Uncharacterized protein 018L of IIV3 | 44382 | 44909 | + | 47.4 | 175 | 2.56e-47 | 0 |
| ORF059L | CGAJLDHN_00024 | hypothetical protein | 45005 | 45400 | + | | | | 0 |
| ORF060R | CGAJLDHN_00025 | Putative MSV199 domain-containing protein 420R of IIV6 | 45422 | 46696 | - | 35.7 | 412 | 3.52e-61 | 0 |
| ORF061R | CGAJLDHN_00026 | N-methyltransferase activity | 46771 | 48102 | - | 63.3 | 286 | 6.81e-122 | 0 |
| ORF062L* | CGAJLDHN_00027 | Uncharacterized protein 016R of IIV3 | 49102 | 50799 | + | 46.4 | 550 | 5.15e-158 | 0 |
| ORF063L | CGAJLDHN_00028 | Uncharacterized protein 016R of IIV3 | 50748 | 52463 | + | 53.0 | 592 | 1.11e-211 | 0 |
| ORF064R | CGAJLDHN_00029 | Uncharacterized protein 072L of IIV3 | 52493 | 52957 | - | 59.6 | 156 | 1.46e-58 | 0 |
| ORF065L | CGAJLDHN_00030 | Uncharacterized protein 073R of IIV3 | 53029 | 53586 | + | 45.6 | 169 | 5.57e-44 | 1 |
| ORF066R | CGAJLDHN_00031 | bis(5'-nucleosyl)-tetraphosphatase (symmetrical) activity | 53721 | 55226 | - | 57.3 | 337 | 7.41e-138 | 0 |
| ORF067L | CGAJLDHN_00032 | protein tyrosine/serine/threonine phosphatase activity | 55310 | 56029 | + | 55.5 | 238 | 4.02e-92 | 0 |
| ORF068L | CGAJLDHN_00033 | hypothetical protein | 56084 | 57193 | + | | | | 0 |
| ORF069R | CGAJLDHN_00034 | Probable matrix metalloproteinase 095L of IIV3 | 57222 | 58301 | - | 34.0 | 303 | 8.23e-52 | 0 |
| ORF070L* | CGAJLDHN_00035 | Erv1/Alr family | 58381 | 58848 | + | 46.3 | 108 | 4.23e-37 | 1 |
| ORF071R | CGAJLDHN_00036 | Uncharacterized protein 082L of IIV3 | 58994 | 59437 | - | 27.5 | 153 | 1.60e-13 | 0 |
| ORF072R | CGAJLDHN_00037 | hypothetical protein | 59501 | 59611 | - | | | | 1 |
| ORF073R | CGAJLDHN_00038 | hypothetical protein | 59634 | 59891 | - | | | | 0 |
| ORF074R | CGAJLDHN_00039 | hypothetical protein | 60092 | 60592 | - | | | **2.29e-15** | 0 |
| ORF075R | CGAJLDHN_00040 | OTU-like cysteine protease | 60640 | 62967 | - | 53.1 | 843 | 1.76e-188 | 0 |

| | | | | | | | | | |
|---|---|---|---|---|---|---|---|---|---|
| ORF076R | CGAJLDHN_00041 | hypothetical protein | 63502 | 63735 | - | | | | 0 |
| ORF077L | CGAJLDHN_00042 | hypothetical protein | 63758 | 63904 | + | | | | 0 |
| ORF078R | CGAJLDHN_00043 | Uncharacterized protein 123L of IIV3 | 63944 | 64342 | - | 38.6 | 132 | 8.37e-21 | 0 |
| ORF079L | CGAJLDHN_00044 | Uncharacterized protein 124R of IIV3 | 64361 | 64996 | + | 36.2 | 224 | 3.02e-23 | 0 |
| ORF080L | CGAJLDHN_00045 | hypothetical protein | 65046 | 65252 | + | | | | 0 |
| ORF081L | CGAJLDHN_00046 | Uncharacterized protein 125R of IIV3 | 65291 | 66154 | + | 48.6 | 257 | 4.40e-90 | 0 |
| ORF082L | CGAJLDHN_00047 | Uncharacterized protein 126R of IIV3 | 66196 | 66483 | + | 46.7 | 105 | 2.78e-23 | 2 |
| ORF083R | CGAJLDHN_00048 | Putative MSV199 domain-containing protein 420R of IIV6 | 66514 | 67782 | - | 40.3 | 412 | 5.26e-90 | 0 |
| ORF084R* | CGAJLDHN_00049 | D5 N terminal like | 68236 | 71055 | - | 69.6 | 941 | 0.0 | 0 |
| ORF085R | CGAJLDHN_00050 | Transmembrane protein 049L of IIV6 | 71167 | 71481 | - | 60.8 | 74 | 3.17e-23 | 3 |
| ORF086R | CGAJLDHN_00051 | DNA polymerase family B | 71559 | 73151 | - | 61.2 | 402 | 7.09e-152 | 0 |
| ORF087R* | CGAJLDHN_00052 | DNA polymerase family B | 73152 | 76328 | - | 60.9 | 870 | 0.0 | 0 |
| ORF088R | CGAJLDHN_00053 | hypothetical protein | 76421 | 76717 | - | | | | 0 |
| ORF089L | CGAJLDHN_00054 | Uncharacterized protein 051L of IIV3 | 76760 | 78226 | + | 31.6 | 320 | 8.81e-40 | 0 |
| ORF090R | CGAJLDHN_00055 | Uncharacterized protein 404L of IIV6 | 78314 | 79045 | - | 62.0 | 216 | 1.21e-96 | 0 |
| ORF091R* | CGAJLDHN_00056 | DNA-directed RNA polymerase subunit 2 | 79056 | 82427 | - | 77.4 | 1137 | 0.0 | 0 |
| ORF092L | CGAJLDHN_00057 | Uncharacterized protein 443R of IIV6 | 82491 | 84833 | + | 37.3 | 614.0 | 1.04e-65 | 0 |
| ORF093L | CGAJLDHN_00058 | hypothetical protein | 84846 | 85076 | + | | | | 0 |
| ORF094R | CGAJLDHN_00059 | Putative MSV199 domain-containing protein 468L of IIV6 | 85113 | 86294 | - | 42.6 | 383 | 2.78e-90 | 0 |
| ORF095R | CGAJLDHN_00060 | Uncharacterized protein 007R of IIV3 | 86352 | 87632 | - | 49.2 | 449 | 3.12e-132 | 0 |
| ORF096R | CGAJLDHN_00061 | Putative MSV199 domain-containing protein 468L of IIV6 | 87717 | 89048 | - | 37.4 | 380 | 1.55e-75 | 0 |
| ORF097R | CGAJLDHN_00062 | DNA ligase | 89138 | 90967 | - | 42.7 | 553 | 4.31e-123 | 0 |
| ORF098L | CGAJLDHN_00063 | hypothetical protein | 91122 | 91337 | + | | | | 0 |
| ORF099R | CGAJLDHN_00064 | Uncharacterized protein 053L of IIV3 | 91506 | 91925 | - | 48.2 | 141 | 2.60e-40 | 0 |
| ORF100R | CGAJLDHN_00065 | Uncharacterized protein 120L of IIV6 | 91958 | 92200 | - | 62.5 | 56 | 4.33e-16 | 0 |
| ORF101R | CGAJLDHN_00066 | Uncharacterized protein 094L of IIV3 | 92229 | 94658 | - | 37.3 | 840 | 3.25e-147 | 0 |
| ORF102R | CGAJLDHN_00067 | hypothetical protein | 94776 | 95576 | - | | | **9.06e-55** | 0 |
| ORF103L* | CGAJLDHN_00068 | Uncharacterized protein 033L of IIV3 | 95677 | 96240 | + | 62.9 | 178 | 7.66e-85 | 0 |
| ORF104R | CGAJLDHN_00069 | hypothetical protein | 96256 | 96645 | - | | | | 0 |
| ORF105R | CGAJLDHN_00070 | Uncharacterized protein 032R of IIV3 | 96721 | 97455 | - | 51.4 | 138 | 1.05e-36 | 0 |
| ORF106R | CGAJLDHN_00071 | hypothetical protein | 97529 | 97948 | - | | | | 0 |
| ORF107L | CGAJLDHN_00072 | uncharacterized protein 030L of IIV3 | 98825 | 99223 | + | 50.0 | 78 | 4.24e-19 | 0 |
| ORF108R* | CGAJLDHN_00073 | Putative kinase protein 029R of IIV3 | 99262 | 99834 | - | 57.4 | 190 | 5.82e-78 | 0 |
| ORF109R | CGAJLDHN_00074 | Uncharacterized protein 028R of IIV3 | 99878 | 100474 | - | 29.9 | 174 | 7.99e-21 | 0 |
| ORF110R | CGAJLDHN_00075 | hypothetical protein | 100585 | 100767 | - | | | | 0 |
| ORF111R | CGAJLDHN_00076 | Thymidylate synthase | 100932 | 101825 | - | 49.8 | 293 | 4.41e-105 | 0 |
| ORF112L | CGAJLDHN_00077 | Uncharacterized protein 061R of IIV3 | 101885 | 102406 | + | 32.9 | 173 | 7.69e-21 | 0 |
| ORF113L | CGAJLDHN_00078 | Uncharacterized protein 061R of IIV3 | 102370 | 103287 | + | 40.4 | 297 | 1.12e-64 | 0 |
| ORF114L | CGAJLDHN_00079 | hypothetical protein | 103367 | 103582 | + | | | | 0 |
| ORF115R | CGAJLDHN_00080 | Putative MSV199 domain-containing protein 420R of IIV6 | 103622 | 104851 | - | 32.5 | 412 | 3.95e-58 | 0 |

| | | | | | | | | | | |
|---|---|---|---|---|---|---|---|---|---|---|
| ORF116L | CGAJLDHN_00081 | Uncharacterized protein 063R of IIV3 | 105062 | 105736 | + | 42.7 | 227 | 2.51e-47 | 0 |
| ORF117R | CGAJLDHN_00082 | hypothetical protein | 105775 | 106431 | - | | | | 0 |
| ORF118R | CGAJLDHN_00083 | DNA topoisomerase 2 | 106443 | 109829 | - | 61.0 | 1129 | 0.0 | 0 |
| ORF119L | CGAJLDHN_00084 | hypothetical protein | 110066 | 110245 | + | | | | 1 |
| ORF120L | CGAJLDHN_00085 | Uncharacterized protein 099R of IIV3 | 110459 | 111448 | + | 59.4 | 202 | 7.01e-72 | 0 |
| ORF121R | CGAJLDHN_00086 | Uncharacterized protein 378R of IIV6 | 111479 | 112123 | - | 70.6 | 204 | 2.59e-73 | 0 |
| ORF122R | CGAJLDHN_00087 | Uncharacterized protein 443R of IIV6 | 112188 | 116096 | - | 39.0 | 1114 | 1.35e-133 | 0 |
| ORF123R | CGAJLDHN_00088 | Uncharacterized protein 229L of IIV6 | 116142 | 117419 | - | 48.8 | 418 | 7.51e-132 | 0 |
| ORF124L | CGAJLDHN_00089 | Putative MSV199 domain-containing protein 238R of IIV6 | 117606 | 119039 | + | 42.4 | 446 | 1.15e-86 | 0 |
| ORF125R | CGAJLDHN_00090 | Uncharacterized protein 045R of IIV3 | 119088 | 119372 | - | 68.1 | 94 | 8.30e-36 | 0 |
| ORF126L | CGAJLDHN_00091 | Putative serine/threonine-protein kinase 040L of IIV3 | 119523 | 120482 | + | 49.3 | 335 | 6.63e-107 | 0 |
| ORF127R | CGAJLDHN_00092 | UPF0213 protein CKO_04549 | 120506 | 120889 | - | 55.6 | 72 | 1.5e-17 | 0 |
| ORF128L | CGAJLDHN_00093 | hypothetical protein | 120954 | 121490 | + | | | | 0 |
| ORF129R* | CGAJLDHN_00094 | Uncharacterized protein 088R of IIV3 | 121538 | 122287 | - | 76.5 | 251 | 5.72e-144 | 0 |
| ORF130R | CGAJLDHN_00095 | Uncharacterized protein L5 | 122293 | 123711 | - | 33.8 | 337 | 1.02e-37 | 0 |
| ORF131R | CGAJLDHN_00096 | mRNA-decapping protein D10 | 124191 | 124859 | - | 40.7 | 216 | 6.86e-45 | 0 |
| ORF132L | CGAJLDHN_00097 | Poxvirus Late Transcription Factor VLTF3 like | 125028 | 126200 | + | 69.3 | 388 | 3.59e-183 | 0 |
| ORF133L | CGAJLDHN_00098 | Uncharacterized protein 042R of IIV3 | 126230 | 126706 | + | 58.5 | 159 | 2.97e-62 | 0 |
| ORF134R | CGAJLDHN_00099 | Putative MSV199 domain-containing protein 468L of IIV6 | 126745 | 127863 | - | 46.0 | 352 | 8.02e-84 | 0 |
| ORF135L | CGAJLDHN_00100 | Putative MSV199 domain-containing protein 420R of IIV6 | 128134 | 129366 | + | 33.3 | 414 | 1.93e-64 | 0 |
| ORF136R | CGAJLDHN_00101 | Ribonucleotide reductase, barrel domain | 129479 | 131857 | - | 57.7 | 780 | 1.38e-303 | 0 |
| ORF137R | CGAJLDHN_00102 | Uncharacterized protein 074L of IIV3 | 131938 | 134034 | - | 45.4 | 808 | 7.71e-215 | 0 |
| ORF138L | CGAJLDHN_00103 | Uncharacterized protein 443R of IIV6 | 134137 | 140991 | + | 32.0 | 1781 | 1.64e-119 | 0 |
| ORF139L | CGAJLDHN_00104 | Uncharacterized protein 043R of IIV3 | 141006 | 141197 | + | 74.6 | 63 | 2.09e-32 | 2 |
| ORF140R* | CGAJLDHN_00105 | Uncharacterized protein 038R of IIV3 | 141407 | 143059 | - | 54.3 | 549 | 2.43e-206 | 0 |
| ORF141L | CGAJLDHN_00106 | hypothetical protein | 143124 | 143573 | + | | | | 1 |
| ORF142R | CGAJLDHN_00107 | Putative thioredoxin-like protein 041R of IIV3 | 143611 | 143967 | - | 55.9 | 118 | 4.59e-47 | 0 |
| ORF143R | CGAJLDHN_00108 | hypothetical protein | 143995 | 144165 | - | | | | 0 |
| ORF144R | CGAJLDHN_00109 | Double-stranded RNA binding motif | 144220 | 144615 | - | | | **3.20e-49** | 0 |
| ORF145R | CGAJLDHN_00110 | Immediate-early protein ICP-46 homolog | 144763 | 146097 | - | 54.0 | 441 | 4.55e-156 | 0 |
| ORF146L* | CGAJLDHN_00111 | protein serine/threonine kinase activity | 146200 | 147741 | + | 58.9 | 513 | 0.0 | 0 |
| ORF147R | CGAJLDHN_00112 | Putative SWIB domain-containing protein 070L of IIV3 | 147780 | 148520 | - | 56.3 | 229 | 1.77e-72 | 0 |
| ORF148R | CGAJLDHN_00113 | Putative MSV199 domain-containing protein 420R of IIV6 | 148655 | 148927 | - | 65.5 | 29 | 2.01e-07 | 0 |
| ORF149L | CGAJLDHN_00114 | N-methyltransferase activity | 148951 | 150084 | + | 69.6 | 313 | 3.39e-148 | 0 |
| ORF150L | CGAJLDHN_00115 | N-methyltransferase activity | 150309 | 151859 | + | 57.0 | 528 | 1.44e-203 | 0 |
| ORF151R | CGAJLDHN_00116 | Putative MSV199 domain-containing protein 238R of IIV6 | 151895 | 153403 | - | 40.2 | 463 | 6.62e-93 | 0 |
| ORF152R* | CGAJLDHN_00117 | Uncharacterized protein 060L of IIV3 | 153546 | 154310 | - | 53.4 | 264 | 1.61e-83 | 0 |
| ORF153L | CGAJLDHN_00118 | hypothetical protein | 154477 | 154875 | + | | | | 0 |
| ORF154L | CGAJLDHN_00119 | Uncharacterized protein 058R of IIV3 | 154952 | 155383 | + | 67.4 | 138 | 6.76e-67 | 0 |
| ORF155R | CGAJLDHN_00120 | hypothetical protein | 155424 | 155564 | - | | | | 0 |

| | | | | | | | | | |
|---|---|---|---|---|---|---|---|---|---|
| ORF156R | CGAJLDHN_00121 | hypothetical protein | 155664 | 156161 | - | | | | 0 |
| ORF157R | CGAJLDHN_00122 | XRN 5'-3' exonuclease N-terminus | 156274 | 157968 | - | 63.2 | 571 | 1.88e-261 | 0 |
| ORF158L | CGAJLDHN_00123 | hypothetical protein | 158143 | 158406 | + | | | | 3 |
| ORF159R | CGAJLDHN_00124 | Belongs to the dihydrofolate reductase family | 158395 | 158937 | - | 37.6 | 178 | 1.61e-38 | 0 |
| ORF160R | CGAJLDHN_00125 | Putative MSV199 domain-containing protein 468L of IIV6 | 158970 | 160286 | - | 42.8 | 355 | 1.05e-90 | 0 |
| ORF161R | CGAJLDHN_00126 | dUTPase | 160688 | 161257 | - | 48.6 | 140 | 2.66e-35 | 1 |
| ORF162L | CGAJLDHN_00127 | Uncharacterized protein 97L | 161388 | 161990 | + | 56.2 | 203 | 3.95e-74 | 0 |
| ORF163L | CGAJLDHN_00128 | Putative MSV199 domain-containing protein 420R of IIV6 | 162018 | 163334 | + | 39.5 | 448 | 1.01e-94 | 0 |
| ORF164R | CGAJLDHN_00129 | Uncharacterized protein 020R of IIV3 | 163405 | 163902 | - | 58.3 | 163 | 4.07e-63 | 0 |
| ORF165L | CGAJLDHN_00130 | Uncharacterized protein 071L of IIV3 | 164812 | 165456 | + | 67.3 | 205 | 2.29e-85 | 0 |
| ORF166R* | CGAJLDHN_00131 | Uncharacterized protein 106R of IIV3 | 165608 | 167041 | - | 61.9 | 465 | 7.06e-208 | 0 |
| ORF167L | CGAJLDHN_00132 | Uncharacterized protein 159L of IIV6 | 167173 | 168645 | + | 33.0 | 233 | 5.54e-28 | 0 |
| ORF168L | CGAJLDHN_00133 | Uncharacterized protein 159L of IIV6 | 168730 | 170208 | + | 32.9 | 231 | 7.33e-26 | 0 |
| ORF169R | CGAJLDHN_00134 | Uncharacterized protein 105R of IIV3 | 170253 | 170984 | - | 65.9 | 246 | 1.73e-112 | 0 |
| ORF170L* | CGAJLDHN_00135 | Putative CTD phosphatase-like protein 355R of IIV3 | 171107 | 171667 | + | 63.8 | 185 | 5.09e-81 | 0 |
| ORF171R | CGAJLDHN_00136 | Zinc finger, C3HC4 type (RING finger) | 171693 | 172358 | - | 50.9 | 228 | 8.46e-62 | 0 |
| ORF172R | CGAJLDHN_00137 | Transmembrane protein 022L of IIV3 | 172421 | 173050 | - | 51.7 | 172 | 2.55e-54 | 6 |
| ORF173R* | CGAJLDHN_00138 | ribonuclease III activity | 173263 | 174099 | - | 76.4 | 280 | 2.67e-148 | 0 |
| ORF174R | CGAJLDHN_00139 | hypothetical protein | 174205 | 175020 | - | | | | 0 |
| ORF175R | CGAJLDHN_00140 | Putative MSV199 domain-containing protein 238R of IIV6 | 175092 | 176585 | - | 42.2 | 446 | 2.28e-85 | 0 |
| ORF176R | CGAJLDHN_00141 | hypothetical protein | 176586 | 176780 | - | | | | 0 |
| ORF177L | CGAJLDHN_00142 | hypothetical protein | 176915 | 177217 | + | | | | 0 |
| ORF178R* | CGAJLDHN_00143 | Putative membrane protein 047R of IIV3 | 177340 | 178602 | - | 75.9 | 274 | 1.21e-170 | 2 |
| ORF179R | CGAJLDHN_00144 | Uncharacterized protein 102R of IIV3 | 178651 | 179004 | - | 59.1 | 115 | 2.52e-35 | 0 |
| ORF180R | CGAJLDHN_00145 | Uncharacterized protein 054L of IIV3 | 179102 | 179935 | - | 48.2 | 247 | 2.33e-74 | 0 |
| ORF181L | CGAJLDHN_00146 | Uncharacterized protein 085L of IIV3 | 180091 | 180561 | + | 65.0 | 137 | 3.59e-63 | 1 |
| ORF182R | CGAJLDHN_00147 | Putative zinc finger protein 012R of IIV3 | 181015 | 182163 | - | 43.2 | 382 | 2.52e-101 | 0 |
| ORF183L* | CGAJLDHN_00148 | Probable serine/threonine-protein kinase 380R of IIV6 | 182360 | 183925 | + | 41.9 | 544 | 1.38e-115 | 0 |
| ORF184R* | CGAJLDHN_00149 | Uncharacterized protein 056L of IIV3 | 183989 | 185014 | - | 43.5 | 345 | 3.71e-88 | 0 |
| ORF185R | CGAJLDHN_00150 | hypothetical protein | 185152 | 185589 | - | | | | 0 |
| ORF186R | CGAJLDHN_00151 | hypothetical protein | 185642 | 186532 | - | | | **2.31e-112** | 0 |
| ORF187L | CGAJLDHN_00152 | Uncharacterized protein 069L of IIV3 | 186579 | 187850 | + | 46.1 | 425 | 2.86e-118 | 0 |
| ORF188R | CGAJLDHN_00153 | Putative Bro-N domain-containing protein 019R of IIV3 | 188012 | 189403 | - | 55.1 | 285 | 2.25e-89 | 0 |
| ORF189R* | CGAJLDHN_00154 | Putative transcription elongation factor S-II-like protein 055R of IIV3 | 189664 | 190086 | - | 46.8 | 139 | 1.74e-42 | 0 |
| ORF190R* | CGAJLDHN_00155 | Uncharacterized protein 035R of IIV3 | 190133 | 192577 | - | 48.9 | 841 | 2.98e-272 | 0 |
| ORF191R | CGAJLDHN_00156 | Uncharacterized protein 035R of IIV3 | 192670 | 193341 | - | 57.7 | 222 | 2.25e-78 | 0 |
| ORF192R | CGAJLDHN_00157 | Uncharacterized protein IIV3-013L | 193455 | 193718 | - | 49.5 | 91 | 4.26e-20 | 1 |
| ORF193R | CGAJLDHN_00158 | hypothetical protein | 193780 | 194097 | - | | | | 0 |
| ORF194R | CGAJLDHN_00159 | hypothetical protein | 194094 | 194318 | - | | | | 0 |

| | | | | | | | | | |
|---|---|---|---|---|---|---|---|---|---|
| ORF195R | CGAJLDHN_00160 | Uncharacterized protein 009R of IIV6 | 194366 | 194626 | - | 60.5 | 81 | 1.22e-34 | 0 |
| ORF196L | CGAJLDHN_00161 | Uncharacterized protein 273R of IIV6 | 194689 | 195969 | + | 43.7 | 142 | 9.42e-26 | 0 |

*ORFs labeled with an asterisk denote core genes recovered and validated with VIGA across all five sequenced IIV genomes from lepidopteran hosts.

E-values: Gene annotation by Blast (normal text) or eggNOG-mapper (bold text).

TM domain content – transmembrane domains (alpha-helices) detected by using DeepTMHMM.
