# Supplementary material for: Invertebrate Iridescent Viruses (Iridoviridae) from the Fall Armyworm, Spodoptera frugiperda
Source: Viruses. 2025 Dec 24;18(1):31. doi: 10.3390/v18010031 (PMC12846554; doi:10.3390/v18010031)
Supplement: Supplementary file 1 [file viruses-18-00031-s001.zip › Table_S9.pdf]

Table S9. AgIIV genome annotation

ORF notation	Locus tag	Product	Start	End	Strand	Identity (%)	Alignment length (bp)	E-Value	TM domain count
ORF001R*	NHMPMIMB_00172	Major capsid protein	1	1383	-	95.8	454	4.24e-317	0
ORF002L	NHMPMIMB_00173	Uncharacterized 15.9 kDa protein in MSP 5'region	1495	1908	+	52.8	106	3.99e-28	0
ORF003R	NHMPMIMB_00174	Uncharacterized protein 404L of IIV6	1930	2670	-	36.0	189	1.54e-28	0
ORF004R	NHMPMIMB_00175	hypothetical protein	2739	3086	-			**1.55e-18**	2
ORF005R*	NHMPMIMB_00176	Putative myristoylated protein 006R of IIV3	3129	4655	-	56.0	493	2.48e-192	3
ORF006L	NHMPMIMB_00177	hypothetical protein	4813	5646	+				0
ORF007R	NHMPMIMB_00178	Uncharacterized protein 004R of IIV3	5668	6918	-	55.6	450	1.58e-138	0
ORF008L	NHMPMIMB_00179	hypothetical protein	6977	7393	+				0
ORF009R	NHMPMIMB_00180	Putative RING finger protein 027R of IIV3	7454	7903	-	30.0	150	2.13e-12	0
ORF010R	NHMPMIMB_00181	Uncharacterized protein 026R of IIV3	7977	8657	-	64.9	225	4.69e-101	0
ORF011R	NHMPMIMB_00182	hypothetical protein	8688	9197	-				1
ORF012L	NHMPMIMB_00183	hypothetical protein	9506	9790	+				0
ORF013R*	NHMPMIMB_00184	Ribonucleoside-diphosphate reductase small chain	9937	11040	-	77.5	351	1.24e-203	0
ORF014R	NHMPMIMB_00185	Uncharacterized protein 050L of IIV3	13518	13988	-	60.7	145	6.55e-57	0
ORF015L	NHMPMIMB_00186	Uncharacterized protein 023R of IIV3	14063	14347	+	72.3	94	1.36e-44	0
ORF016R	NHMPMIMB_00187	Putative Bro-N domain-containing protein 019R of IIV3	14373	15704	-	51.6	316	1.09e-93	0
ORF017R	NHMPMIMB_00188	Uncharacterized protein 107R of IIV3	16145	17071	-	46.0	309	2.30e-79	0
ORF018R	NHMPMIMB_00189	Putative MSV199 domain-containing protein 468L of IIV6	17149	18438	-	41.4	355	3.11e-84	0
ORF019R	NHMPMIMB_00190	Uncharacterized protein 017R of IIV3	18729	19361	-	59.4	212	1.19e-82	0
ORF020R	NHMPMIMB_00191	Uncharacterized protein 017R of IIV3	19358	19645	-	52.8	89	8.49e-19	0
ORF021L	NHMPMIMB_00192	Uncharacterized protein 422L of IIV6	19771	20355	+	43.1	174	2.11e-34	0
ORF022R	NHMPMIMB_00193	hypothetical protein	20393	20797	-				0
ORF023R	NHMPMIMB_00194	Probable cysteine proteinase 024R of IIV3	20841	22286	-	56.3	487	5.7e-198	1
ORF024R	NHMPMIMB_00195	Putative FAS1 domain-containing protein 081L of IIV3	22365	22619	-	40.2	82	1.35e-09	0
ORF025R	NHMPMIMB_00001	Putative KilA-N domain-containing protein 006L of IIV6	23309	23536	-	42.2	64	2.60e-06	0
ORF026R	NHMPMIMB_00002	Putative MSV199 domain-containing protein 468L of IIV6	23672	24061	-	44.6	112	1.33e-20	0
ORF027R	NHMPMIMB_00003	Uncharacterized protein 017R of IIV3	24722	25204	-	50.0	148	2.73e-39	0
ORF028L	NHMPMIMB_00004	Uncharacterized protein 422L of IIV6	25619	25960	+	42.2	116	1.34e-19	0
ORF029R	NHMPMIMB_00005	hypothetical protein	26307	26639	-				0
ORF030R	NHMPMIMB_00006	Probable cysteine proteinase 024R of IIV3	27015	27329	-	67.3	104	5.56e-50	0
ORF031R	NHMPMIMB_00007	Probable cysteine proteinase 024R of IIV3	27751	28125	-	39.5	119	2.09e-18	0
ORF032R	NHMPMIMB_00008	Putative FAS1 domain-containing protein 081L of IIV3	28288	28605	-	36.5	85	7.49e-09	0
ORF033R	NHMPMIMB_00009	hypothetical protein	28617	28766	-				0
ORF034R	NHMPMIMB_00010	hypothetical protein	28995	29216	-				0
ORF035L	NHMPMIMB_00011	Uncharacterized protein 115R of IIV3	29351	29608	+	60.3	78.0	5.66e-29	0
ORF036L	NHMPMIMB_00012	Uncharacterized protein 119R of IIV3	29722	30234	+	70.6	51.0	5.52e-20	0

ORF037R	NHMPMIMB_00013	hypothetical protein	30282	30980	-				0
ORF038R	NHMPMIMB_00014	Belongs to the protein-tyrosine phosphatase family. Non-receptor class dual specificity subfamily	31044	31514	-	46.6	133	2.39e-32	0
ORF039L*	NHMPMIMB_00015	High mobility group protein homolog 068R of IIV3	31665	32255	+	83.7	202	1.46e-116	0
ORF040L	NHMPMIMB_00016	hypothetical protein	32333	32581	+				0
ORF041R	NHMPMIMB_00017	RNA polymerase Rpb1, domain 5	32646	33005	-	71.9	135	1.09e-54	0
ORF042R*	NHMPMIMB_00018	DNA-directed RNA polymerase subunit 1	33448	36648	-	71.8	1085	0.0	0
ORF043R*	NHMPMIMB_00019	XPG I-region	37137	38234	-	52.8	377	1.14e-130	0
ORF044R	NHMPMIMB_00020	hypothetical protein	38351	39439	-				0
ORF045R	NHMPMIMB_00021	hypothetical protein	39478	40053	-			**6.36e-36**	2
ORF046R	NHMPMIMB_00022	Putative MSV199 domain-containing protein 468L of IIV6	40113	41267	-	41.1	382	3.71e-88	0
ORF047L	NHMPMIMB_00023	Uncharacterized protein 113L of IIV3	41373	43700	+	51.4	803	1.87e-269	0
ORF048L	NHMPMIMB_00024	Trypsin Inhibitor like cysteine rich domain	43771	43998	+	49.1	57	1.01e-16	0
ORF049R	NHMPMIMB_00025	Uncharacterized protein 112R of IIV3	44041	44382	-	48.6	111	2.98e-31	1
ORF050R	NHMPMIMB_00026	phosphatase activity	44458	44700	-	64.1	78	1.16e-30	0
ORF051R	NHMPMIMB_00027	phosphatase activity	44652	44882	-			**1.81e-16**	0
ORF052L	NHMPMIMB_00028	Uncharacterized protein 001R of IIV3	44968	45528	+	34.4	154	5.43e-20	0
ORF053R	NHMPMIMB_00029	Uncharacterized protein 092R of IIV3	45702	46217	-	65.3	173	3.82e-71	0
ORF054R	NHMPMIMB_00030	kinase activity	46232	46741	-			**8.81e-69**	0
ORF055R	NHMPMIMB_00031	hypothetical protein	46839	48260	-				0
ORF056L	NHMPMIMB_00032	Uncharacterized protein 032R of IIV3	48509	49378	+	39.8	133	5.16e-18	0
ORF057L	NHMPMIMB_00033	hypothetical protein	49538	50269	+				0
ORF058L	NHMPMIMB_00034	Uncharacterized protein 018L of IIV3	50488	51015	+	47.4	175	2.56e-47	0
ORF059L	NHMPMIMB_00035	hypothetical protein	51109	51513	+				0
ORF060R	NHMPMIMB_00036	Putative MSV199 domain-containing protein 420R of IIV6	51696	52787	-	33.5	385	6.27e-57	0
ORF061R	NHMPMIMB_00037	N-methyltransferase activity	52862	54193	-	63.6	286	5.95e-123	0
ORF062L*	NHMPMIMB_00038	Uncharacterized protein 016R of IIV3	55174	58536	+	50.4	1150	0.0	0
ORF063R	NHMPMIMB_00039	Uncharacterized protein 072L of IIV3	58566	59030	-	59.6	156	1.46e-58	0
ORF064L	NHMPMIMB_00040	Uncharacterized protein 073R of IIV3	59109	59666	+	46.2	169	2.24e-43	1
ORF065R	NHMPMIMB_00041	bis(5'-nucleosyl)-tetraphosphatase (symmetrical) activity	59793	61298	-	57.6	337	9.16e-139	0
ORF066L	NHMPMIMB_00042	protein tyrosine/serine/threonine phosphatase activity	61382	62101	+	55.5	238	8.08e-92	0
ORF067L	NHMPMIMB_00043	hypothetical protein	62156	63265	+				0
ORF068R	NHMPMIMB_00044	Probable matrix metalloproteinase 095L of IIV3	63295	64392	-	34.0	303	9.72e-52	0
ORF069L*	NHMPMIMB_00045	Erv1 / Alr family	64473	64829	+	47.5	101	2.87e-35	0
ORF070R	NHMPMIMB_00046	Uncharacterized protein 082L of IIV3	65085	65528	-	27.5	153	1.60e-13	0
ORF071R	NHMPMIMB_00047	hypothetical protein	65592	65702	-				1
ORF072R	NHMPMIMB_00048	Putative MSV199 domain-containing protein 420R of IIV6	65733	66971	-	31.1	412	5.06e-55	0
ORF073R	NHMPMIMB_00049	hypothetical protein	67183	67683	-			**2.29e-15**	0
ORF074R	NHMPMIMB_00050	OTU-like cysteine protease	67732	69207	-	57.3	386	6.76e-83	0
ORF075R	NHMPMIMB_00051	OTU-like cysteine protease	69176	70555	-	64.8	469	1.20e-171	0
ORF076R	NHMPMIMB_00052	hypothetical protein	70683	70916	-				0

ORF077L	NHMPMIMB_00053	hypothetical protein	70939	71085	+				0
ORF078R	NHMPMIMB_00054	Uncharacterized protein 123L of IIV3	71124	71522	-	38.6	132	8.37e-21	0
ORF079L	NHMPMIMB_00055	Uncharacterized protein 124R of IIV3	71541	72176	+	36.2	224	3.02e-23	0
ORF080L	NHMPMIMB_00056	Uncharacterized protein 443R of IIV6	72226	76101	+	42.4	857	1.44e-122	0
ORF081L	NHMPMIMB_00057	Uncharacterized protein 125R of IIV3	76140	77003	+	48.6	257	4.4e-90	0
ORF082L	NHMPMIMB_00058	Uncharacterized protein 126R of IIV3	77045	77332	+	46.7	105	2.78e-23	2
ORF083R	NHMPMIMB_00059	Putative MSV199 domain-containing protein 420R of IIV6	77363	78631	-	40.0	412	3.73e-90	0
ORF084R	NHMPMIMB_00060	hypothetical protein	79248	79469	-				0
ORF085R	NHMPMIMB_00061	hypothetical protein	79482	79874	-	75.8	128	3.26e-63	0
ORF086R*	NHMPMIMB_00062	D5 N terminal like	79889	82300	-	67.9	803	0.0	0
ORF087R	NHMPMIMB_00063	Transmembrane protein 049L of IIV6	82398	82706	-	58.7	75	8.37e-23	3
ORF088R*	NHMPMIMB_00064	DNA polymerase family B	82783	86478	-	62.3	1232	0.0	0
ORF089R	NHMPMIMB_00065	hypothetical protein	86646	86867	-				0
ORF090L	NHMPMIMB_00066	Uncharacterized protein 051L of IIV6	86910	88376	+	31.6	320	8.81e-40	0
ORF091R	NHMPMIMB_00067	Uncharacterized protein 404L of IIV6	88462	89193	-	63.9	216	1.28e-98	0
ORF092R*	NHMPMIMB_00068	DNA-directed RNA polymerase subunit 2	89204	92575	-	77.3	1137	0.0	0
ORF093L	NHMPMIMB_00069	Uncharacterized protein 443R of IIV6	92639	94618	+	44.3	350	4.82e-53	0
ORF094L	NHMPMIMB_00070	hypothetical protein	94631	94861	+				0
ORF095R	NHMPMIMB_00071	Putative MSV199 domain-containing protein 468L of IIV6	94898	96040	-	42.3	381	9.05e-91	0
ORF096R	NHMPMIMB_00072	Uncharacterized protein 007R of IIV3	96136	97416	-	49.2	449	3.12e-132	0
ORF097R	NHMPMIMB_00073	Putative MSV199 domain-containing protein 468L of IIV6	97501	98835	-	39.5	380	1.35e-80	0
ORF098R	NHMPMIMB_00074	DNA ligase	98925	100754	-	42.5	553	8.54e-123	0
ORF099L	NHMPMIMB_00075	hypothetical protein	100909	101124	+				0
ORF100R	NHMPMIMB_00076	Uncharacterized protein 053L of IIV3	101293	101712	-	48.2	141	3.69e-40	0
ORF101R	NHMPMIMB_00077	Uncharacterized protein 120L of IIV6	101745	101987	-	62.5	56	4.33e-16	0
ORF102R	NHMPMIMB_00078	Uncharacterized protein 094L of IIV3	102016	104445	-	37.3	840	2.12e-148	0
ORF103R	NHMPMIMB_00079	hypothetical protein	104557	105369	-			**2.29e-56**	0
ORF104L*	NHMPMIMB_00080	Uncharacterized protein 033L of IIV3	105470	106033	+	62.9	178	7.66e-85	0
ORF105R	NHMPMIMB_00081	hypothetical protein	106049	106354	-				0
ORF106R	NHMPMIMB_00082	Uncharacterized protein 032R of IIV3	106440	107159	-	51.4	138	9.05e-37	0
ORF107R	NHMPMIMB_00083	hypothetical protein	107233	107652	-				0
ORF108L	NHMPMIMB_00084	uncharacterized protein 030L of IIV3	108511	108909	+	48.7	78	1.19e-18	0
ORF109R*	NHMPMIMB_00085	Putative kinase protein 029R of IIV3	108948	109520	-	57.4	190	4.10e-78	0
ORF110R	NHMPMIMB_00086	Uncharacterized protein 028R of IIV3	109564	110454	-	32.6	282	1.98e-38	0
ORF111R	NHMPMIMB_00087	Thymidylate synthase	110607	111500	-	49.1	293	7.20e-104	0
ORF112L	NHMPMIMB_00088	Uncharacterized protein 061R of IIV3	111559	112965	+	37.2	478	5.45e-95	0
ORF113L	NHMPMIMB_00089	hypothetical protein	113045	113260	+				0
ORF114R	NHMPMIMB_00090	Putative MSV199 domain-containing protein 420R of IIV6	113301	114530	-	32.8	412	7.31e-59	0
ORF115R	NHMPMIMB_00091	hypothetical protein	114597	115118	-				0
ORF116L	NHMPMIMB_00092	Uncharacterized protein 063R of IIV3	115215	115889	+	42.7	227	2.51e-47	0
ORF117R	NHMPMIMB_00093	hypothetical protein	115942	116583	-				0
ORF118R	NHMPMIMB_00094	DNA topoisomerase 2	116604	119996	-	61.2	1129	0.0	0

ORF119L	NHMPMIMB_00095	hypothetical protein	120233	120412	+				1
ORF120L	NHMPMIMB_00096	Uncharacterized protein 396L	120452	122944	+	35.8	930	4.26e-142	0
ORF121L	NHMPMIMB_00097	Uncharacterized protein 099R of IIV3	122962	123951	+	59.4	202	7.01e-72	0
ORF122R	NHMPMIMB_00098	Uncharacterized protein 378R of IIV6	123982	124629	-	69.3	205	1.90e-73	0
ORF123R	NHMPMIMB_00099	Uncharacterized protein 443R of IIV6	124677	131285	-	33.3	1404	3.54e-100	0
ORF124L	NHMPMIMB_00100	Uncharacterized protein 074L of IIV3	131388	133481	+	44.7	812	9.29e-216	0
ORF125L	NHMPMIMB_00101	Ribonucleotide reductase, barrel domain	133601	135940	+	57.9	780	4.94e-303	0
ORF126L	NHMPMIMB_00102	Putative MSV199 domain-containing protein 238R of IIV6	136010	137473	+	41.6	442	9.74e-81	0
ORF127R	NHMPMIMB_00103	Uncharacterized protein 042R of IIV3	137566	138042	-	58.8	160	2.09e-62	0
ORF128R	NHMPMIMB_00104	Poxvirus Late Transcription Factor VLTF3 like	138072	139244	-	69.3	388	7.24e-183	0
ORF129L	NHMPMIMB_00105	mRNA-decapping protein D10	139413	140081	+	40.3	216	2.73e-44	0
ORF130L*	NHMPMIMB_00106	Putative Bro-N domain-containing protein 069L of IIV6	140326	141483	+	53.8	80	5.59e-15	0
ORF131L*	NHMPMIMB_00107	Uncharacterized protein 088R of IIV3	141489	142238	+	76.1	251	8.12e-144	0
ORF132R	NHMPMIMB_00108	hypothetical protein	142284	142820	-				0
ORF133L	NHMPMIMB_00109	UPF0213 protein CKO_04549	142885	143268	+	55.6	72	1.5e-17	0
ORF134R	NHMPMIMB_00110	Putative serine/threonine-protein kinase 040L of IIV3	143292	144251	-	49.3	335	6.63e-107	0
ORF135L	NHMPMIMB_00111	Uncharacterized protein 045R of IIV3	144403	144687	+	68.1	94	8.30e-36	0
ORF136R	NHMPMIMB_00112	Putative MSV199 domain-containing protein 468L of IIV6	144759	145862	-	41.2	376	8.34e-73	0
ORF137L	NHMPMIMB_00113	Uncharacterized protein 229L of IIV6	145961	147238	+	48.6	418	7.51e-132	0
ORF138L	NHMPMIMB_00114	Uncharacterized protein 443R of IIV6	147284	150043	+	48.8	640	3.29e-123	0
ORF139L	NHMPMIMB_00115	Uncharacterized protein 043R of IIV3	150216	150407	+	74.6	63	2.09e-32	2
ORF140R*	NHMPMIMB_00116	Uncharacterized protein 038R of IIV3	150616	152268	-	54.3	549	3.97e-205	0
ORF141L	NHMPMIMB_00117	hypothetical protein	152333	153034	+				1
ORF142R	NHMPMIMB_00118	Putative thioredoxin-like protein 041R of IIV3	153075	153431	-	55.1	118	2.64e-46	0
ORF143R	NHMPMIMB_00119	hypothetical protein	153459	153611	-				0
ORF144R	NHMPMIMB_00120	Double-stranded RNA binding motif	153682	154077	-			**3.19e-49**	0
ORF145R	NHMPMIMB_00121	Immediate-early protein ICP-46 homolog	154224	155558	-	54.2	441	6.92e-158	0
ORF146L*	NHMPMIMB_00122	protein serine/threonine kinase activity	155661	157064	+	59.4	461	6.24e-191	0
ORF147R	NHMPMIMB_00123	Putative SWIB domain-containing protein 070L of IIV3	157242	157982	-	56.3	229	6.23e-73	0
ORF148R	NHMPMIMB_00124	Putative MSV199 domain-containing protein 420R of IIV6	158261	158545	-	65.5	29	2.32e-07	0
ORF149L	NHMPMIMB_00125	N-methyltransferase activity	158569	161478	+	62.4	984	0.0	0
ORF150R	NHMPMIMB_00126	Putative MSV199 domain-containing protein 468L of IIV6	161514	162683	-	38.4	383	2.47e-81	0
ORF151R	NHMPMIMB_00127	Uncharacterized protein 060L of IIV6	162831	163595	-	53.4	264	1.61e-83	0
ORF152L	NHMPMIMB_00128	hypothetical protein	163762	164160	+				0
ORF153L	NHMPMIMB_00129	Uncharacterized protein 058R of IIV3	164240	164671	+	67.4	138	6.76e-67	0
ORF154R	NHMPMIMB_00130	hypothetical protein	164712	164852	-				0
ORF155R	NHMPMIMB_00131	hypothetical protein	164949	165446	-				0
ORF156R	NHMPMIMB_00132	XRN 5'-3' exonuclease N-terminus	165561	167252	-	63.1	572	9.01e-262	0
ORF157L	NHMPMIMB_00133	hypothetical protein	167427	167690	+				3
ORF158R	NHMPMIMB_00134	dihydrofolate reductase activity	168035	168229	-	52.1	48	6.27e-12	0
ORF159R	NHMPMIMB_00135	Putative MSV199 domain-containing protein 468L of IIV6	168262	169575	-	42.1	356	1.41e-84	0
ORF160R	NHMPMIMB_00136	dUTPase	169674	170243	-	46.8	141	1.33e-35	1

ORF161L	NHMPMIMB_00137	Uncharacterized protein 97L of IIV3	170375	170977	+	56.2	203	2.79e-74	0
ORF162L	NHMPMIMB_00138	Putative MSV199 domain-containing protein 420R of IIV6	171005	172330	+	39.5	448	1.11e-94	0
ORF163R	NHMPMIMB_00139	Uncharacterized protein 020R of IIV3	172384	172881	-	57.7	163	2.34e-62	0
ORF164L	NHMPMIMB_00140	Uncharacterized protein 071L of IIV3	173792	174427	+	69.5	197	5.04e-86	0
ORF165R	NHMPMIMB_00141	hypothetical protein	174641	174763	-				0
ORF166R*	NHMPMIMB_00142	Uncharacterized protein 106R of IIV3	174810	176243	-	62.2	465	2.02e-207	0
ORF167L	NHMPMIMB_00143	Uncharacterized protein 159L of IIV6	176375	177850	+	33.9	233	1.21e-28	0
ORF168L	NHMPMIMB_00144	Uncharacterized protein 159L of IIV6	177927	179408	+	32.0	231	4.98e-24	0
ORF169R	NHMPMIMB_00145	Uncharacterized protein 105R of IIV3	179453	180184	-	65.9	246	6.06e-113	0
ORF170L*	NHMPMIMB_00146	Putative CTD phosphatase-like protein 355R of IIV3	180310	180873	+	64.0	186	5.29e-81	0
ORF171R	NHMPMIMB_00147	Zinc finger, C3HC4 type (RING finger)	180895	181560	-	50.9	228	4.22e-62	0
ORF172R	NHMPMIMB_00148	Transmembrane protein 022L of IIV3	181623	182252	-	51.7	172	4.48e-55	6
ORF173R*	NHMPMIMB_00149	ribonuclease III activity	182475	183311	-	76.8	280	3.27e-149	0
ORF174R	NHMPMIMB_00150	hypothetical protein	183426	184295	-				0
ORF175R	NHMPMIMB_00151	Putative MSV199 domain-containing protein 238R of IIV6	184367	186055	-	42.2	446	1.40e-84	0
ORF176L	NHMPMIMB_00152	hypothetical protein	186277	186456	+				0
ORF177R*	NHMPMIMB_00153	Putative membrane protein 047R of IIV3	186578	187888	-	76.6	274	2.20e-170	2
ORF178R	NHMPMIMB_00154	Uncharacterized protein 102R of IIV3	187937	188290	-	57.6	118	3.57e-35	0
ORF179R	NHMPMIMB_00155	Uncharacterized protein 054L of IIV3	188388	189221	-	48.2	247	2.33e-74	0
ORF180L	NHMPMIMB_00156	Uncharacterized protein 085L of IIV3	189378	189848	+	65.0	137	3.59e-63	1
ORF181R	NHMPMIMB_00157	Putative zinc finger protein 012R of IIV3	190317	191462	-	42.9	382	6.91e-101	0
ORF182L*	NHMPMIMB_00158	Probable serine/threonine-protein kinase 380R of IIV6	191662	193221	+	41.7	545	9.03e-113	0
ORF183R*	NHMPMIMB_00159	Uncharacterized protein 056L of IIV3	193292	194311	-	43.8	345	3.46e-88	0
ORF184R	NHMPMIMB_00160	hypothetical protein	194453	194890	-				0
ORF185R	NHMPMIMB_00161	hypothetical protein	194943	195833	-			**1.15e-112**	0
ORF186L	NHMPMIMB_00162	Uncharacterized protein 069L of IIV3	195880	197151	+	46.4	425	2.51e-119	0
ORF187R	NHMPMIMB_00163	Putative MSV199 domain-containing protein 420R of IIV6	197814	198548	-	46.4	151	1.32e-31	0
ORF188R*	NHMPMIMB_00164	Putative transcription elongation factor S-II-like protein 055R of IIV3	198964	199386	-	46.8	139	1.74e-42	0
ORF189R*	NHMPMIMB_00165	Uncharacterized protein 035R of IIV3	199433	202045	-	49.4	899	6.49e-298	0
ORF190R	NHMPMIMB_00166	Uncharacterized protein 035R of IIV3	202206	202640	-	60.4	149	6.70e-51	0
ORF191R	NHMPMIMB_00167	Uncharacterized protein IIV3-013L	202754	203017	-	49.5	91	4.26e-20	1
ORF192R	NHMPMIMB_00168	hypothetical protein	203079	203396	-				0
ORF193R	NHMPMIMB_00169	hypothetical protein	203393	203617	-				0
ORF194R	NHMPMIMB_00170	Uncharacterized protein 009R of IIV6	203665	203925	-	61.7	81	1.74e-34	0
ORF195L	NHMPMIMB_00171	Uncharacterized protein 273R of IIV6	203990	205339	+	44.4	142	6.28e-26	0

*ORFs labeled with an asterisk denote core genes recovered and validated with VIGA across all five sequenced IIV genomes from lepidopteran hosts.
E-values: Gene annotation by Blast (normal text) or eggNOG-mapper (bold text).
TM domain content – transmembrane domains (alpha-helices) detected by using DeepTMHMM.
