# Supplementary figures and images for: Invertebrate Iridescent Viruses (Iridoviridae) from the Fall Armyworm, Spodoptera frugiperda
Source: Viruses. 2025 Dec 24;18(1):31. doi: 10.3390/v18010031 (PMC12846554; doi:10.3390/v18010031)

**Figure S5.** Annotation of linear genome of SfIIV-Chi

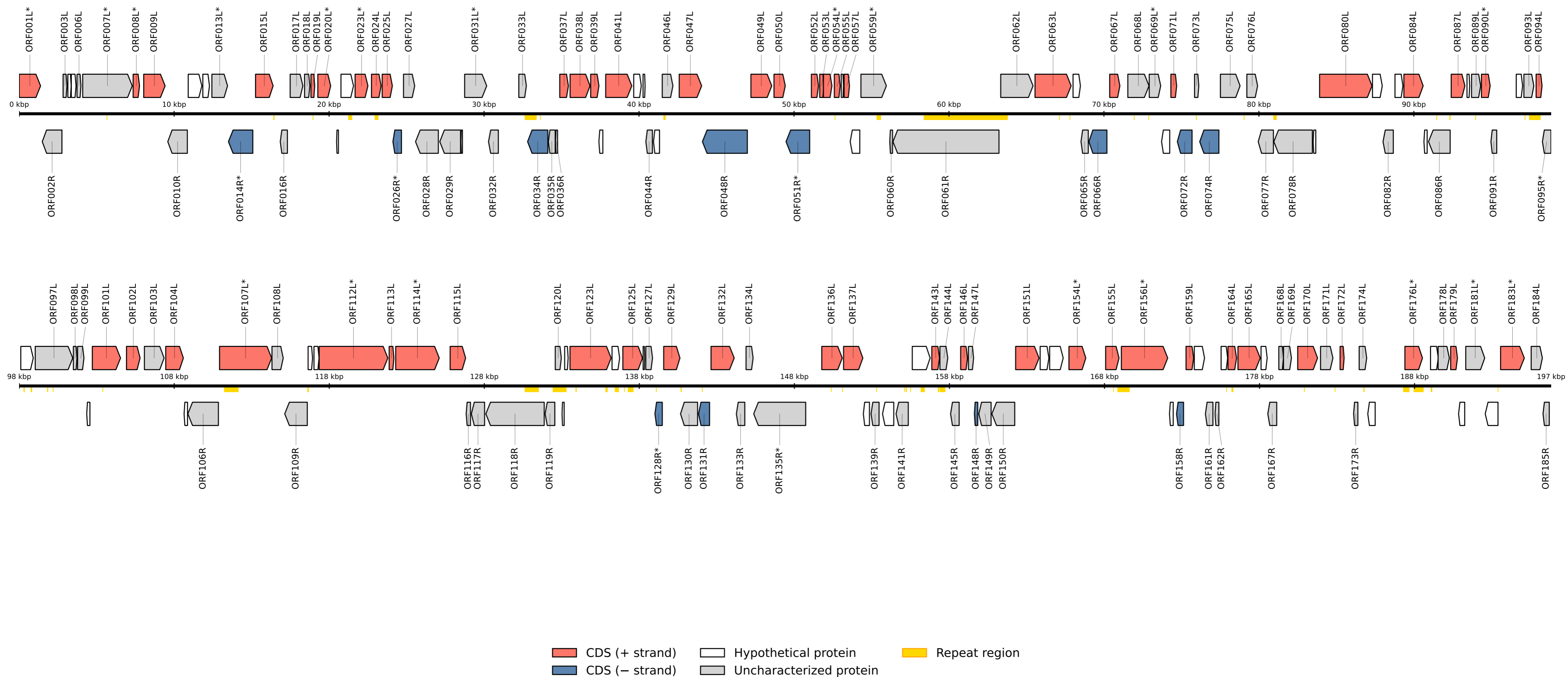

Supplement: Supplementary file 1 [file viruses-18-00031-s001.zip › Fig_S5.pdf]

**Figure S6.** Annotation of linear genome of SfIIV-Ver

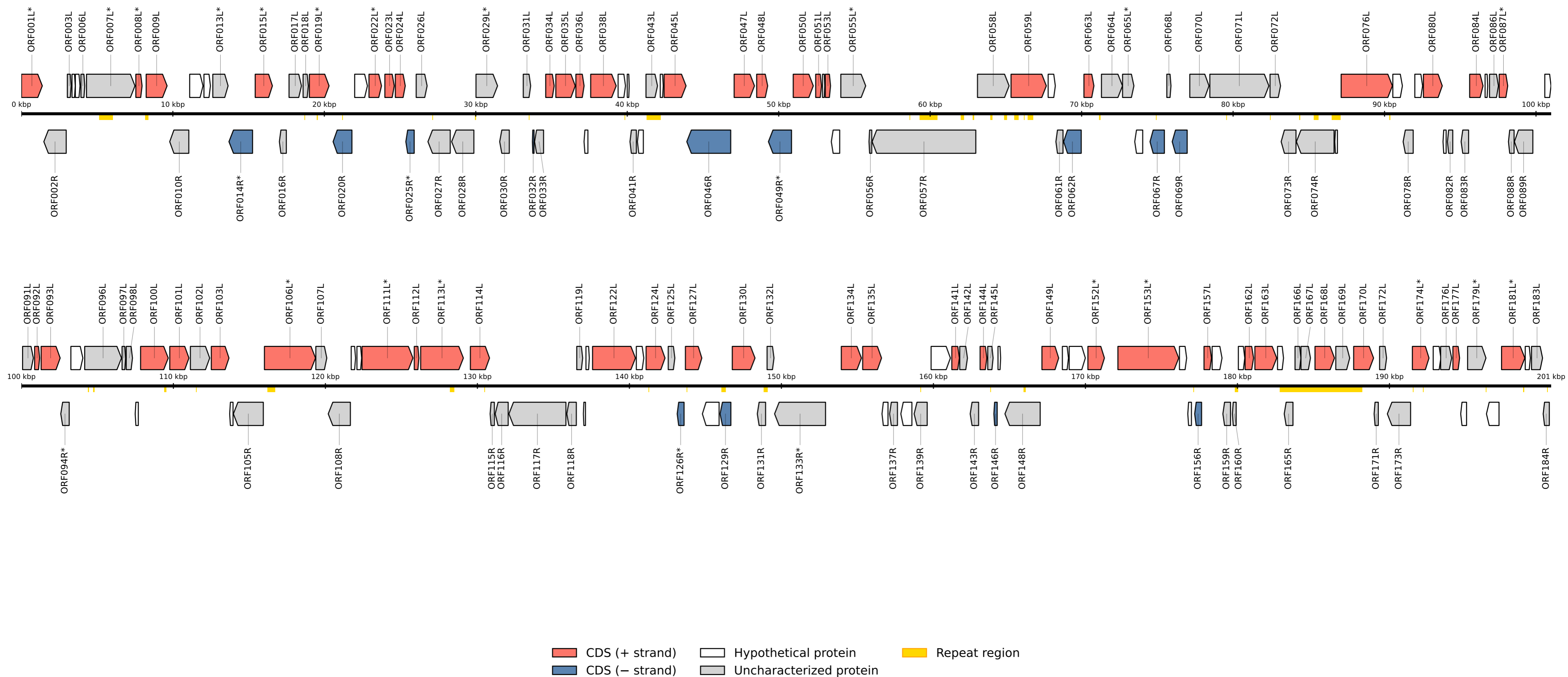

Supplement: Supplementary file 1 [file viruses-18-00031-s001.zip › Fig_S6.pdf]

**Figure S7.** Annotation of linear genome of SfIIV-Arg

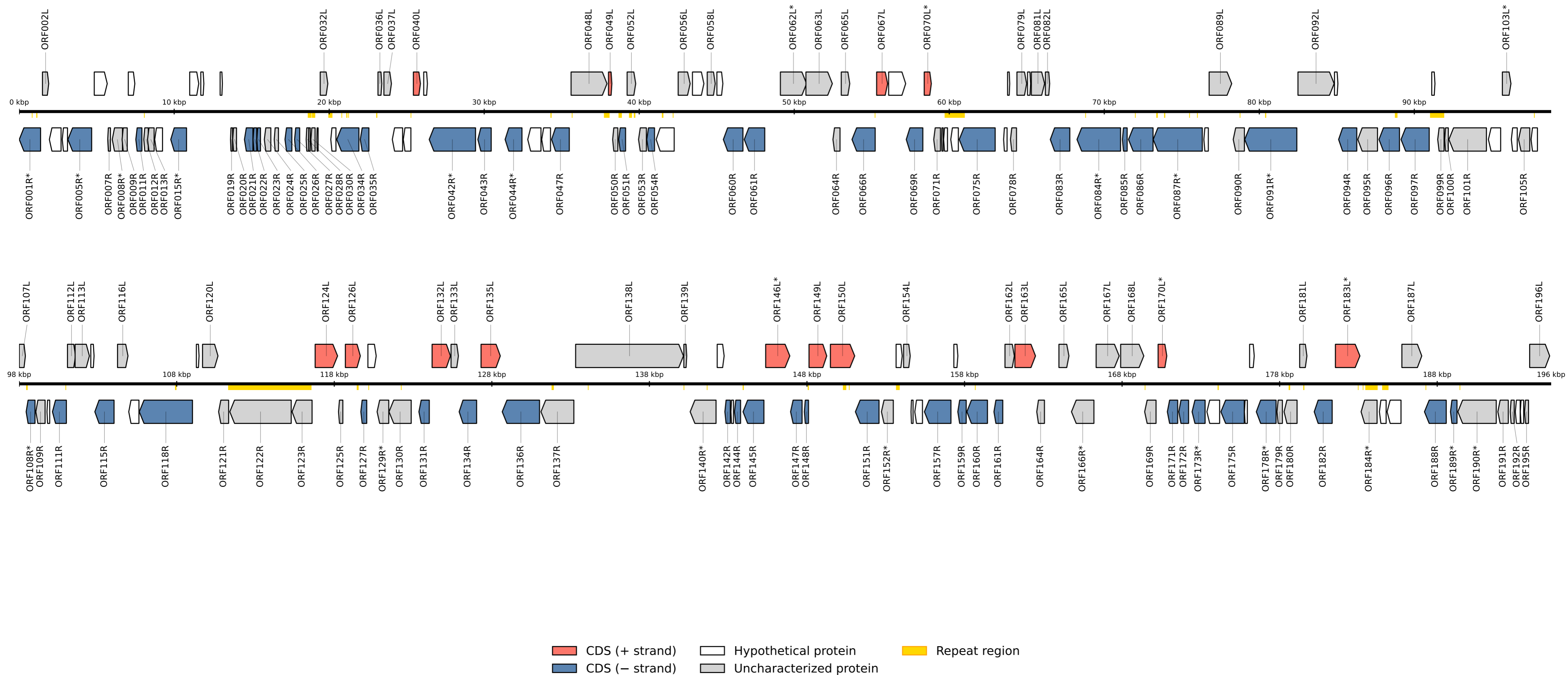

Supplement: Supplementary file 1 [file viruses-18-00031-s001.zip › Fig_S7.pdf]

**Figure S8.** Annotation of linear genome of AgIIIV

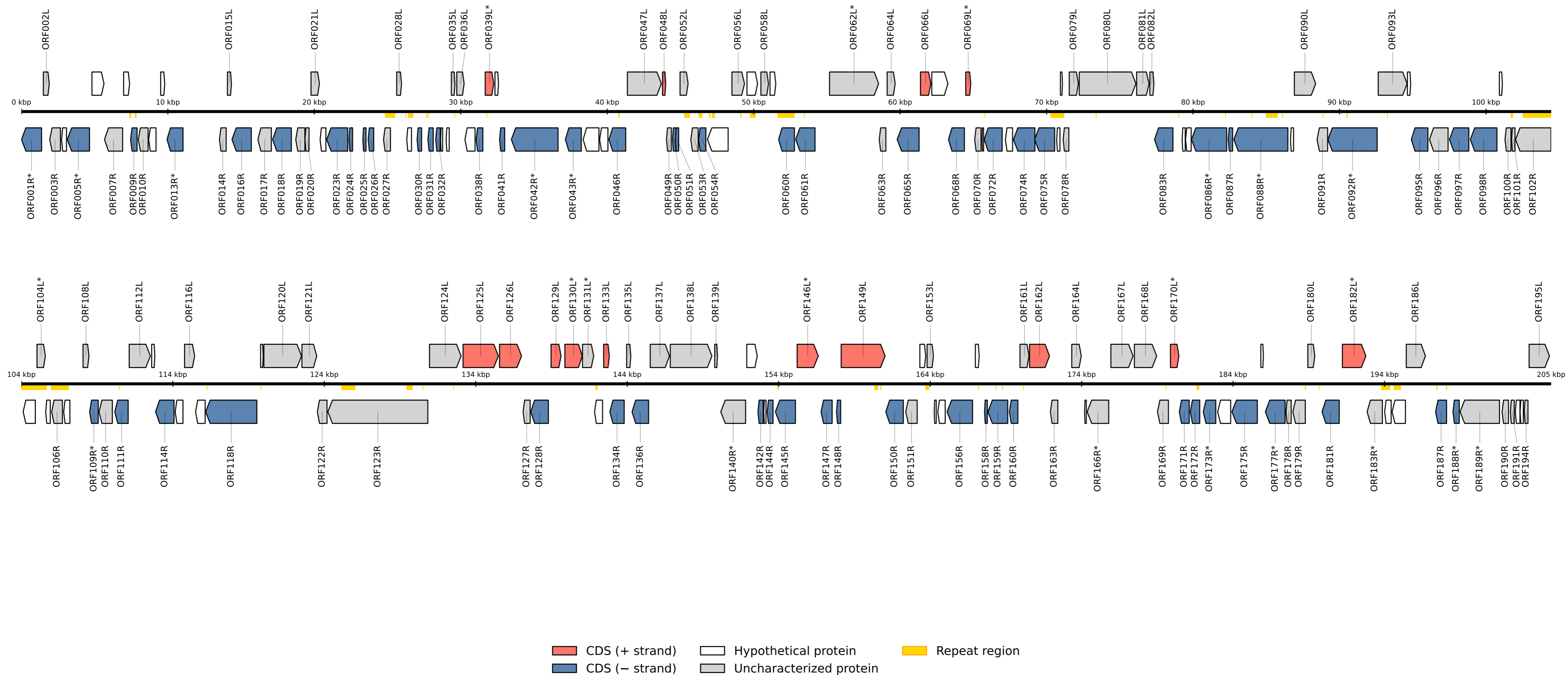

Supplement: Supplementary file 1 [file viruses-18-00031-s001.zip › Fig_S8.pdf]

**Figure S9.** Annotation of linear genome of IIV30C

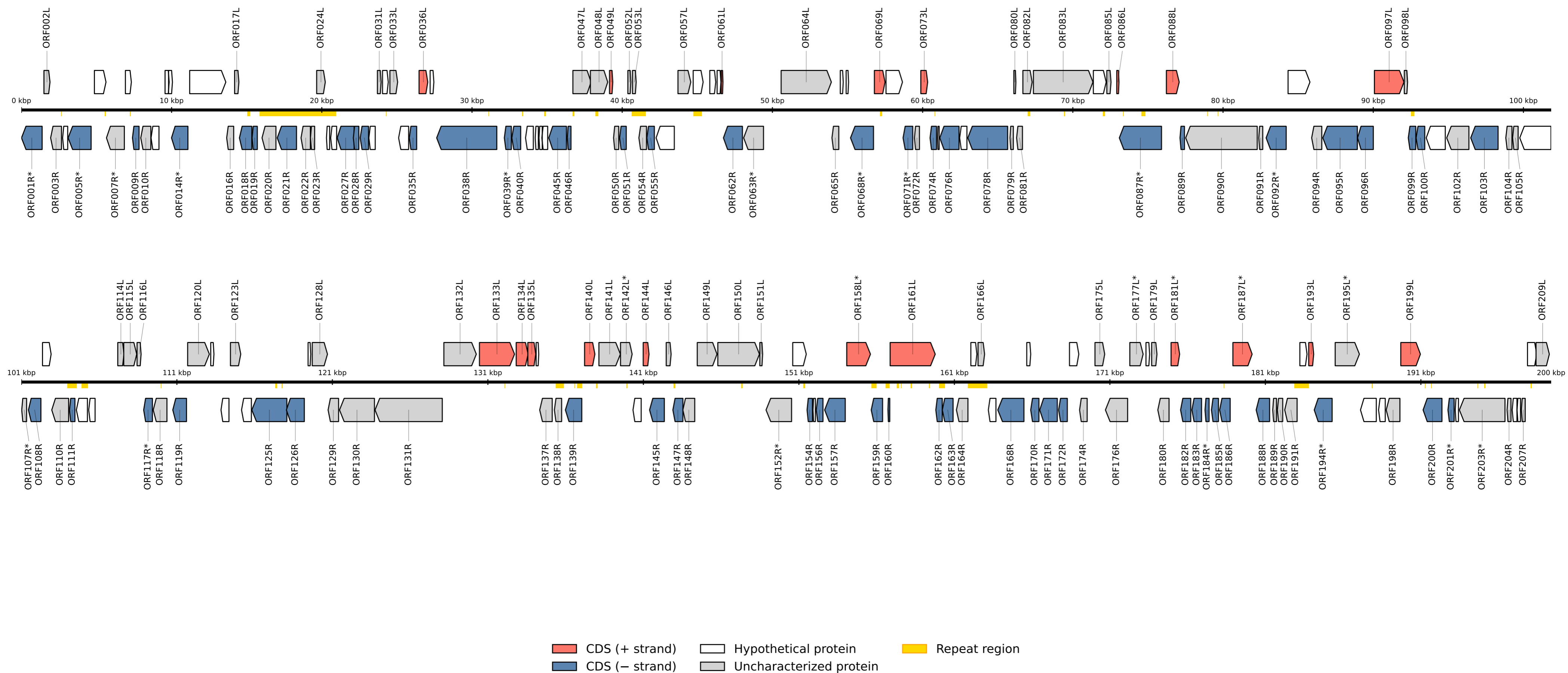

Supplement: Supplementary file 1 [file viruses-18-00031-s001.zip › Fig_S9.pdf]
